# Protective Effects of Polydatin from Grapes and *Reynoutria japonica* Houtt. on Damaged Macrophages Treated with Acetaminophen

**DOI:** 10.3390/nu14102077

**Published:** 2022-05-16

**Authors:** Can Liu, Wenyi Wang, Kaixin Zhang, Qiudi Liu, Tongyao Ma, Li Tan, Lanqing Ma

**Affiliations:** 1 Key Laboratory for Northern Urban Agriculture of Ministry of Agriculture and Rural Affairs, Beijing University of Agriculture, Beijing 102206, China; liucan808@163.com (C.L.); wangwenyi0712@163.com (W.W.); zhangkaixinxinaini@163.com (K.Z.); liuqiudi2022@126.com (Q.L.); 18813007029@163.com (T.M.); tanli0825@163.com (L.T.); 2 Beijing Advanced Innovation Center for Tree Breeding by Molecular Design, Beijing University of Agriculture, Beijing 102206, China

**Keywords:** acetaminophen, macrophages, in vitro, polydatin, protective effects

## Abstract

The unregulated use of acetaminophen (APAP), an antipyretic and analgesic drug, harms hepatocytes and kidney cells, leading to liver failure and acute kidney injury. Herein, we investigate whether APAP damages macrophages in the immune system by observing its effects on macrophage proliferation and apoptosis. Using proteomics, we analyzed the effects of APAP on macrophage protein expression profiles and evaluated whether polydatin, the active ingredient in grapes and wine, can repair the damaged cells. The results showed that APAP alters the morphology and physiological processes of macrophages, inhibits macrophage proliferation, and promotes apoptosis. We observed 528 differentially expressed proteins when 500 µg/mL APAP was administered to the cells. These proteins are involved in biological processes including cell division, apoptosis, and acute phase response. Overall, our findings demonstrate that APAP harms the immune system by damaging macrophages and that polydatin can repair this damage.

## 1. Introduction

**Figure 1:**
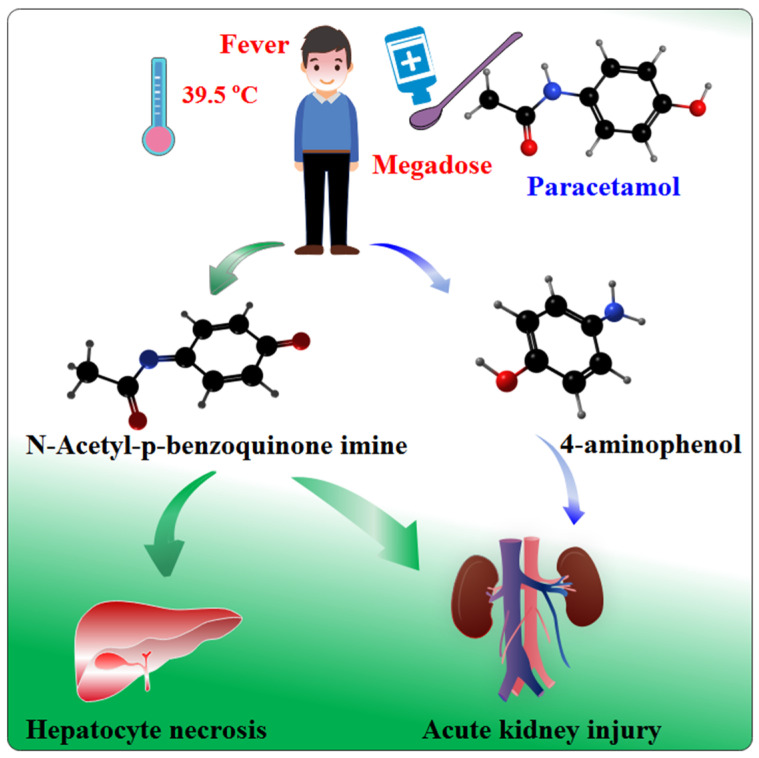
Pathways of liver and kidney damage caused by excessive acetaminophen (APAP) administration.

Acetaminophen (APAP), also known as paracetamol, is a widely used over the counter (OTC) antipyretic and analgesic drug [1,2]. APAP is known to be toxic to cells and can cause liver damage when the maximum daily dose exceeds 4 g [3]. Acute liver failure or even death can occur at doses greater than 10 g [4]. Liver damage due to APAP is becoming increasingly concerning worldwide [5,6]. When APAP enters hepatocytes, most of the drug is converted into nontoxic substances through the glucuronide or sulfonation pathways [7]. Excess APAP is catalyzed by the enzyme cytochrome P450 2E1 (CYP2E1) to form the highly electrophile-active intermediate N-acetyl-p-benzoquinoneimine (NAPQI) [8], which binds to mitochondrial proteins in hepatocytes and triggers a series of damaging reactions, such as mitochondrial dysfunction, which can cause apoptotic damage and eventually hepatocyte necrosis [9]. In addition to hepatic damage caused by acetamides, some studies have shown that overdose or long-term use of APAP may cause acute kidney injury (AKI) [10], and the incidence of AKI after APAP overdose is 2–10%. The mechanism of AKI damage caused by APAP is complex and primarily involves the production of toxic metabolic substances in vivo, oxidative stress, inflammation, mitochondrial dysfunction, and endoplasmic reticulum stress [11,12]. Jaeschke Murray et al. showed that APAP is metabolized in the kidney to produce NAPQI but is also deacetylated to produce a nephrotoxic product, 4-aminophenol (PAP), which is one of the mechanisms underlying the resulting nephrotoxicity [13] (Figure 1).

Immune cells are an important class of cells that participate in the body′s inflammatory response and help maintain homeostasis [14]. Macrophages are immune cells that differentiate from monocytes in the blood after they have penetrated the blood vessels [15]; the main functions of macrophages are to phagocytose and digest cellular debris and pathogens [16] and to activate lymphocytes or other immune cells in response to pathogens [17]. During inflammation, macrophages are transported and recruited through the bloodstream to the site of inflammation, where they phagocytose foreign substances, release antibacterial substances, and clear pathogens [18]. As such, macrophages play an important role in the body′s immune defense, inflammation regulation, and immune surveillance [19]. Currently, studies regarding APAP toxicity focus on liver damage; however, reports on whether APAP disrupts the immune system, especially immune cells, are still lacking.

Polydatin, a secondary metabolite of grapes and *Reynoutria japonica* Houtt [20], is an antitoxin secreted in response to an adverse event or pathogen attack. For example, polydatin synthesis increases sharply upon UV irradiation, mechanical damage, and fungal infection, and, thus, it is considered a phytoalexin. Grapes are known to have anti-aging effects, and resveratrol is an important active ingredient in grape juice. The amounts of polydatin and resveratrol in different grape juices are comparable [21]. Furthermore, polydatin is the main active ingredient in a Chinese medicine derived from *R. japonica* [22]. Polydatin is a stilbene compound that is produced through the glycosylation of resveratrol during the growth of grapes and *R. japonica* [23]. Polydatin has several medicinal properties, such as scavenging free radicals [24], regulating lipid regulation [25], lowering cholesterol [26], and anti-Parkinsonism [27] and anti-inflammatory [28] activity.

The immune system plays an important defensive role, such as suppressing viruses and reducing fevers. Current studies on cellular damage by APAP have focused on hepatocytes and kidney; however, whether APAP negatively affects the immune system and whether it damages the immune cells are still unclear. Therefore, we studied the effects of APAP on the physiology of macrophages and analyzed the effects of polydatin on the damaged macrophages.

## 2. Materials and Methods

### 2.1. Materials

RAW264.7 mouse macrophage leukemia cells were purchased from the Cell Resource Center, Peking Union Medical College. We used acetaminophen (D1909159, Aladdin, Shanghai, China); polydatin (SP8420, Solarbio, Beijing, China); Dulbecco′s Modified Eagle Medium (DMEM) without sodium pyruvate (high glucose, with penicillin and streptomycin) (12100, Solarbio, Beijing, China), fetal bovine serum (11012-8611, EVERY GREEN), and the Annexin V-FITC Apoptosis Detection Kit (BA00101) (Beijing BoaoSen Biotechnology Co., Ltd., Beijing, China) in the in vitro experiments.

### 2.2. Methods

#### 2.2.1. Cell Culture

RAW264.7 mouse mononuclear macrophages were cultured in a culture dish containing fetal bovine serum and DMEM medium (volume ratio of serum to DMEM medium 1:9) at a CO_2_ saturation of 5% at 37 °C in an incubator (SANYO MCO-18AIC UV). The cells were subsequently processed when they reached 80–90% confluency.

#### 2.2.2. Cell Injury Model Construction and Drug Treatment

In a 96-well plate, 200 µL of medium and 2 × 10^4^ cells were added to each well and incubated at 37 °C with 5% CO_2_ until the cells adhered to the plate. Thereafter, the medium was discarded, and cells were cultured in medium with APAP added for 24 h at final concentrations of 0, 100, and 500 μg/mL. Three replicate wells were used for each concentration. Next, the cells were exposed to polydatin in a drug concentration of 5 μg/mL and cultured at 37 °C for 24 h. The grouping of the different administered cells is defined as follows: APAP100 (low-dose, 100 μg/mL APAP), APAP500 (high-dose, 500 μg/mL APAP), and APAP500 + HU (500 μg/mL APAP + 5 μg/mL polydatin).

#### 2.2.3. Cell Morphology Observation

Morphological changes were observed during cell culture. The cells were cultured in complete medium with final APAP concentrations of 0, 50, 100, 200, and 500 μg/mL at 37 °C for 24 h, respectively. Then, the cells were exposed to polydatin in a drug concentration of 5 μg/mL for 24 h. Morphological changes were observed using an inverted microscope (400×) and photographed for analysis.

#### 2.2.4. MTT Assay for Cell Viability

Cells were cultured in a medium containing a final APAP concentration of 500 μg/mL for 24 h. Subsequently, polydatin was added to the medium so that the final concentrations of polydatin were 0, 1, 5, 10, and 20 μg/mL, successively, and the cells were further cultured. After 24 h of incubation, 20 μL of MTT solution was added to each well for 4 h at 37 °C, and the MTT was reacted with succinate dehydrogenase in the living cells to produce blue-violet crystalline Formazan. The next step was to aspirate the medium, add 150 μL of DMSO, mix it in a shaker for 20 min, and measure the absorbance at 490 nm with an enzyme-labeled instrument (xMark, BIO-RAD, Hercules, CA, USA) to calculate the cell survival rate.

#### 2.2.5. Apoptosis Assay

Cells were cultured in complete medium with final APAP concentrations of 0, 50, 100, and 500 μg/mL for 24 h. Then, the cells were exposed to polydatin in a drug con-centration of 5 μg/mL for 24 h. The cells were collected and centrifuged at 1000 r/min for 5 min. Thereafter, the supernatant was discarded, and the cells were resuspended in 0.01 M PBS. The cells were counted, and 5 µL of Annexin V-FITC and 5 µL of propidium iodide were added to 2 × 10^5^ cells in a tube suitable for flow cytometry. Each tube was shaken gently to mix the contents and incubated in the dark at 25 °C for 15 min. Next, 400 µL of 1× binding buffer was added and mixed, and flow cytometry was performed (ACEA NovoCyte D313).

#### 2.2.6. Preparation and Digestion of Proteins

Liquid nitrogen was added to the samples, which were then ground thoroughly. The ground samples were then transferred to 1.5 mL centrifuge tubes. Sample lysate (250 μL) was transferred to each centrifuge tube, and protease inhibitor PMSF was added to reach a final concentration of 1 mM. An ultrasonicator was used to rupture the cells, followed by centrifugation at 12,000× *g* for 10 min. Thereafter, the supernatant was collected, and five times the volume of acetone was added for protein precipitation. We then collected the precipitate after centrifugation and isolated the protein. Protein (50 μg) was taken from each sample, and all samples were adjusted to the same concentration and volume by dilution with lysis solution. To this protein solution dithiothreitol (DTT) was added at a final concentration of 5 mM and incubated at 55 °C for 30 min. The solutions were cooled on ice until they reached room temperature. Iodoacetamide was then added to the solution to reach a final concentration of 10 mM, and the samples were placed in the dark at room temperature for 15 min. Acetone was added to the protein solution at a volume ratio of 6:1 (acetonitrile:protein solution, *v*/*v*) to precipitate the protein and incubated overnight at −20 °C. The protein precipitate was collected by centrifugation at 8000× *g* for 10 min (high-speed low-temperature centrifuge (Eppendorf Centrifuge); 100 μL of TEAB (200 mM) was added to re-solubilize the precipitate; 1 mg/mL trypsin-TPCK was added to the protein solution at a volume ratio of 1:50 (enzyme solution:protein solution, *v*/*v*); and the protein solutions were digested at 37 °C overnight. After digestion, the samples were lyophilized, dissolved in 100 mM TEAB buffer, and labeled with TMTpro reagent.

#### 2.2.7. LC-MS/MS Analysis

The peptides were separated and identified using liquid chromatography and mass spectrometry. Peptide separation was performed using an RP-C18 chromatographic column (75 μm × 15 cm, Thermo Fisher, Waltham, MA, USA) with mobile phase A: H_2_O-formic acid (0.1%, *v*/*v*); mobile phase B: acetonitrile-formic acid (0.1%, *v*/*v*); mobile phase flow rate of 300 nL/min; detection wavelengths UV 210 nm and 280 nm; and gradient elution conditions of 0–3 min, 0–5% B; 3–51 min, 5–22% B; 51–63 min, 22–32% B; 63–64 min, 32–90% B; 64–70 min, 90% B; 70–71 min, 90–2% B; and 71–80 min, 2% B. Molecular weight identification of the peptides was performed using mass spectrometry. The resolution of the primary mass spectrum was 70,000, the automatic gain control value was 1 × 10^6^, and the maximum ion injection time was 50 ms. Scanning was performed in the 300–1600 charge-to-mass ratio (*m*/*z*) range, and secondary spectra were scanned for the 10 highest peaks. Secondary scanning was performed in positive ion mode, and the collision energy was set to 32 eV. The resolution of the secondary mass spectrum was 35,000, the automatic gain control was 2 × 10^5^, the maximum ion injection time was 80 ms, and the dynamic exclusion time was 30 s.

#### 2.2.8. Data Analysis

Qualitative protein search and analysis were performed using Proteome Discover 2.4 software (Thermo Fisher Scientific). The parameters were set as follows: trypsin was used to digest the protein, carbamidomethyl modification of cysteine was set as static, the TMT modifications on the N-terminal of the peptide and lysine were also set as static; the oxidation modification on the methionine and acetylation modification of the N-terminal of the peptide were set as dynamic; and the missed cleavages value was set to 2. Analysis of protein functions was conducted with reference to protein information annotated in Uniprot, the Kyoto Encyclopedia of Genes and Genomes (KEGG), Gene Ontology (GO), and Eukaryotic/Clusters of Orthologous Groups (KOG/COG) databases. Credible proteins were screened using the criteria Score Sequest HT > 0 and unique peptide ≥ 1, and differentially expressed proteins were screened using the criteria *p*-value < 0.05 and fold change (FC) = 1.5. A hierarchical clustering dendrogram of sample Euclidean distance was drawn based on the Euclidean distances between the samples. Protein interaction analysis of differential proteins was performed using the online tools STRING (https://cn.string-db.org/) and Cytoscape 3.6.1. All data analysis was performed using SPSS 20 software (IBM Corp., Armonk, NY, USA). Results are expressed as t-test values for comparison between two groups, and one-way ANOVA was used for comparison between multiple groups to analyze the differences between groups. In all cases, *p* < 0.05 is considered significant and *p* < 0.01 highly significant.

## 3. Results and Discussion

### 3.1. Effect of Drugs on Cell Proliferation

**Figure 2:**
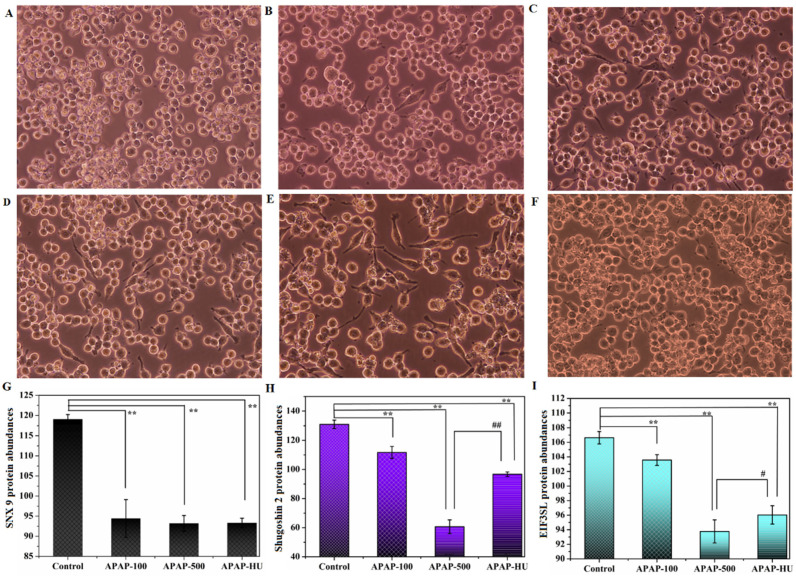
Acetaminophen (APAP) inhibits cell proliferation and regulates the expression of proteins involved in cell division. (**A**) Control group, (**B**) 50 μg/mL APAP group, (**C**) 100 μg/mL APAP group, (**D**) 200 μg/mL APAP group, (**E**) 500 μg/mL APAP group, and (**F**) 500 μg/mL APAP + 5 μg/mL polydatin group. (**G**) Differentially expressed proteins of sorting nexin-9 protein (SNX9). (**H**) Differentially expressed proteins of Shugoshin 2. (**I**) Differentially expressed proteins of eukaryotic translation initiation factor 3 subunit L (EIF3SL) in the control group vs. APAP100 vs. APAP500 vs. APAP500-HU (** *p* < 0.01 vs. Control; # *p* < 0.05 vs. APAP-500; ## *p* < 0.01 vs. APAP-500).

As shown in Figure 2A–E, APAP inhibited cell growth (Figure S1). Some cells were observed to differentiate and extend pseudopods, a small number of spindle-shaped cells appeared, and the cell survival rate increased when polydatin was added (Figure 2F). The results indicate that APAP is cytotoxic and that the addition of low-dose polydatin to the culture medium can antagonize the inhibition of cell growth by APAP. These results indicate that APAP can stimulate cell differentiation and inhibit cell growth; the addition of polydatin to the cell culture medium reduced the number of spindle-shaped cells in the microscopic field and increased the cell density, indicating that polydatin has a certain effect of protecting macrophages.

The function of sorting nexin-9 protein (SNX9) is to participate in cytokinesis and intracellular vesicle transport at the end of mitosis [29], and its low expression inhibits cell division and suppresses proliferation. As shown in Figure 2G, APAP decreased the expression of SNX9 in cells compared with the controls. The effect of APAP500 + HU vs. APAP500 on SNX9 expression was not significant.

Proteomics results showed that the downregulation of Shugoshin 2, a protein involved in cell division and proliferation, inhibited cell proliferation [30] (Figure 2H) after APAP administration. Shugoshin 2 appeared to be downregulated in the cells after APAP administration but appeared upregulated in cells damaged by high-dose APAP with the addition of polydatin, indicating that polydatin may repair cellular damage.

Eukaryotic translation initiation factor 3 subunit L (EIF3SL) is encoded by the *Eif3l* gene and is a component of the eukaryotic translation initiation factor 3 complex. Low expression of this protein inhibits cell division and suppresses cell proliferation [31]. As shown in Figure 2I, APAP downregulated the expression of EIF3SL in macrophages relative to the control group, while partial restoration of protein expression occurred after the addition of polydatin to the APAP-injured cells.

These results showing differential protein expression appeared with our cell morphology observations, which indicated that APAP inhibits cell division and reduces cell proliferation. In contrast, adding polydatin appears to repair the damaged cells and promote cell proliferation.

### 3.2. Effect of Drugs on Apoptosis

**Figure 3:**
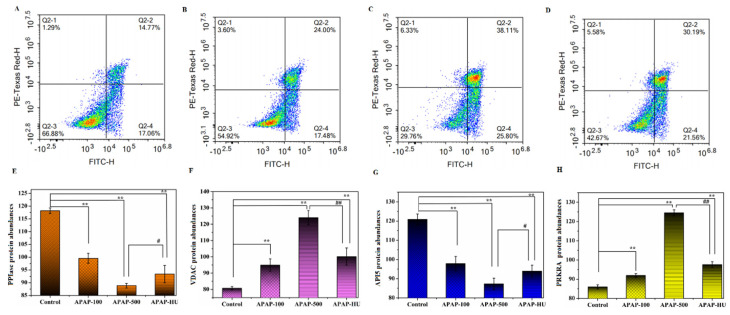
Acetaminophen (APAP) induces apoptosis and participates in the regulation of apoptotic protein expression in cells. (**A**) 50 μg/mL APAP group, (**B**) 100 μg/mL APAP group, (**C**) 500 μg/mL APAP group, and (**D**) 500 μg/mL APAP + 5 μg/mL polydatin group. (**E**) Differentially expressed proteins of peptidyl-prolyl cis-trans isomerase (PPIase). (**F**) Differentially expressed proteins of voltage-dependent anion-selective channel protein (VDAC). (**G**) Differentially expressed proteins of apoptosis inhibitor 5 protein (API5). (**H**) Differentially expressed proteins of interferon-inducible double-stranded RNA-dependent protein kinase activator A (PRKRA) in the control group vs. APAP100 vs. APAP500 vs. APAP500-HU. (** *p* < 0.01 vs. Control; # *p* < 0.05 vs. APAP-500; ## *p* < 0.01 vs. APAP-500).

The apoptosis assay revealed apoptosis and necrosis after APAP administration, indicating that APAP is cytotoxic, inhibiting the growth of macrophages and promoting apoptosis. Cells apoptosis is shown in Figure 3, and the number of necrotic and apoptotic cells increased with APAP concentration (Figure 3A–C). These results suggest that APAP induces necrosis and apoptosis, which is consistent with the results of toxicological studies of acetaminophen in liver cells. The apoptosis rate was significantly reduced after polydatin addition relative to the APAP group (Figure 3D), suggesting that polydatin can repair APAP-induced macrophage apoptosis.

Peptidyl-prolyl cis-trans isomerase (PPIase) protects the mitochondria against oxidative stress [32,33]; however, downregulation of this protein induces oxidative damage in mitochondria, thus promoting apoptosis. APAP downregulated PPIase expression in test cells, with the greatest downregulation observed in the high-dose APAP-treated cells (Figure 3E). However, PPIase was upregulated in the high-dose group after the addition of polydatin.

The function of voltage-dependent anion-selective channel protein (VDAC) is to promote mitochondrial autophagy [34], and high expression of this protein promotes apoptosis [35,36]. As shown in Figure 3F, APAP upregulated VDAC in the test cells, with the greatest upregulation in the high-dose APAP group. In comparison, the addition of polydatin decreased the expression level of this protein.

Apoptosis inhibitor 5 protein (API5), an anti-apoptotic factor with inhibitory effects on apoptosis [37], was downregulated by APAP, thus promoting apoptosis (Figure 3G). However, the addition of polydatin to APAP-injured cells upregulated API5 expression.

The high expression of interferon-inducible double-stranded RNA-dependent protein kinase activator A (PRKRA) promotes apoptosis [38]. APAP exposure upregulated this protein in the test cells, while polydatin downregulated the expression (Figure 3H).

Collectively, these results show that the addition of polydatin to APAP-damaged macrophages resulted in the partial repair of cellular protein expression levels (Table S1).

### 3.3. Differential Protein Screening

**Figure 4:**
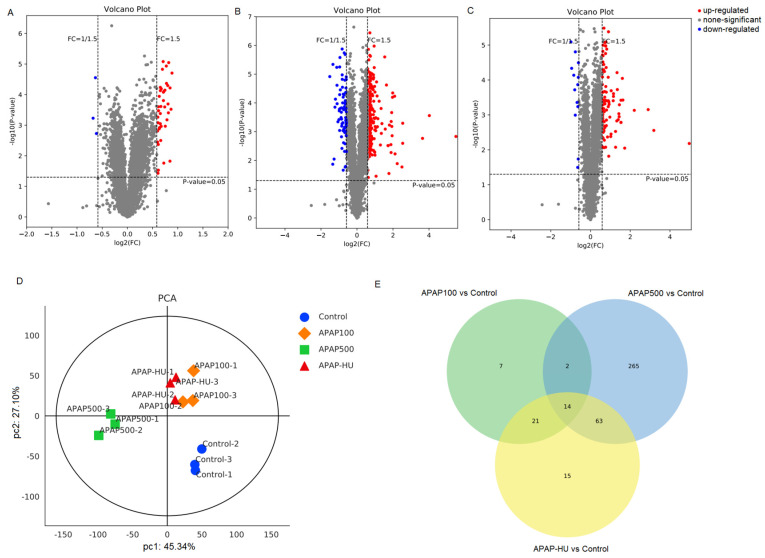
Volcano plot, principal component analysis (PCA), and Venn diagram for 200 differentially expressed proteins of macrophages treated with acetaminophen (APAP) in the presence or absence of polydatin (HU). (**A**) APAP100 vs. control group, (**B**) APAP500 vs. control group, (**C**) APAP500-HU vs. control group. Gray represents proteins without significant differences; blue represents differential proteins significantly downregulated; and red represents differential proteins significantly upregulated. (**D**) PCA plot in which each point in the coordinate system represents a single replicate in a grouped experiment, and different colors distinguish different groups. (**E**) Venn diagram.

A total of 5772 plausible proteins were identified by screening, and 528 differentially expressed proteins were significantly expressed (Table S1). Figure 4 shows a volcano plot of all differential proteins in the various drug treatment groups. Compared with the control group, low-dose APAP upregulated 38 proteins and downregulated 3 proteins in the test cells (Figure 4A), whereas high-dose APAP upregulated 211 proteins and downregulated 67 proteins (Figure 4B). The addition of polydatin to the cells after APAP injury upregulated 47 proteins and significantly downregulated 13 proteins compared to the controls (Figure 4C).

### 3.4. Principal Component Analysis (PCA)

PCA score plots were drawn using standardized quantitative protein expression data from the control, APAP100 (low-dose), APAP500 (high-dose), and APAP500 + HU (high-dose + polydatin) groups (Figure 4D). The PCA plot shows that the control group is located in the fourth quadrant and each dosing group is located in different quadrants, indicating that the protein expression profiles of the cells were affected in all dosing groups. The APAP100 group is distributed in the first quadrant of the PCA plot, and the protein expression profile of the APAP500 high-dose group is located in the third quadrant; however, the protein expression profiles of the macrophages in the high-dose group (APAP-HU) were similar to those in the APAP100 group and were all distributed in the first quadrant of the PCA plot. This indicates that the administration of polydatin reduced the effect of high-dose APAP on the protein expression profiles of the cells. In summary, we found that polydatin plays a role in the repair of cells damaged by high-dose APAP.

### 3.5. Venn Diagram Analysis

We used Venn analysis to determine differential protein characteristics and commonalities in each test group (Figure 4E). There were 44 differentially expressed proteins between the cells in the low-dose APAP administration group (APAP100) and the control group, indicating that APAP can regulate the protein expression profiles in macrophages. When the APAP dose was elevated, the number of differentially expressed proteins between the cells in the high-dose APAP group (APAP500 group) and the control group increased substantially to 344, indicating the greatest change in protein expression profile regulation. When high-dose APAP was administered, the addition of polydatin (APAP-HU) reduced the number of differentially expressed proteins from 334 (APAP500) to 113.

### 3.6. Clustering Analysis of Differential Protein Expression Levels

**Figure 5:**
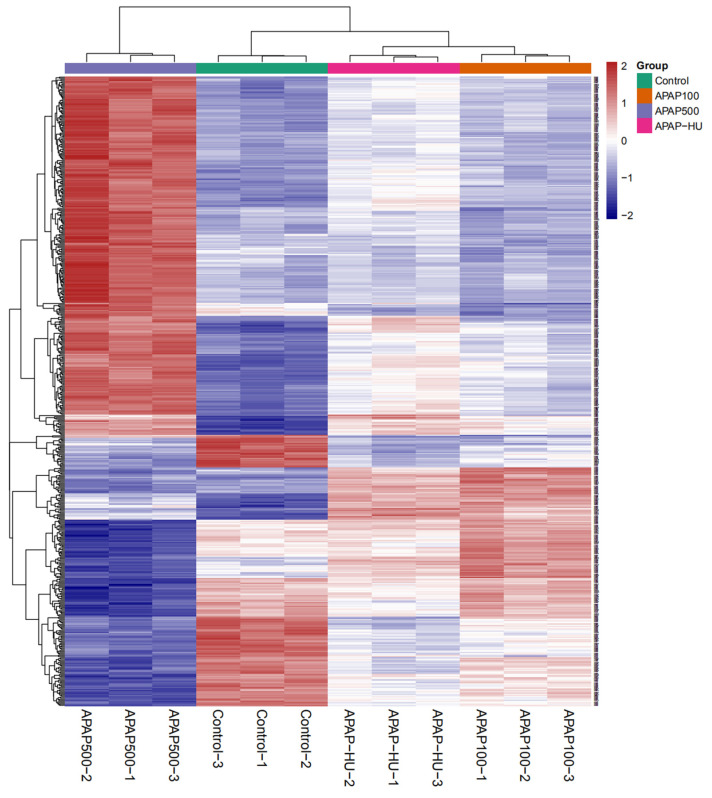
Clustering analysis of differentially expressed proteins of macrophages treated with acetaminophen (APAP) in the presence or absence of polydatin (HU).

Based on the abundance of differential proteins in the various groups, we performed hierarchical clustering analysis at the minimum clustering distance (Figure 5). The parallel samples could be clustered into one class (clusters appeared in the same class), indicating that the results were reproducible. Longitudinal and transverse clustering indicates the similarity of the abundance of different biochemical indicators among samples; the closer the distance between the two groups and the shorter the branch length, the more similar the abundance of these two groups. The APAP-HU and APAP100 groups had shorter branch lengths compared with the APAP-HU and APAP500 groups, suggesting that the addition of polydatin in the high-dose APAP group reduced cell damage caused by APAP. When the clustering distance was further expanded, the control, APAP-HU, and APAP100 samples clustered into one group, indicating that APAP500 caused the greatest damage to cells and HU produced a protective effect.

### 3.7. Protein Interaction Network Diagram

**Figure 6:**
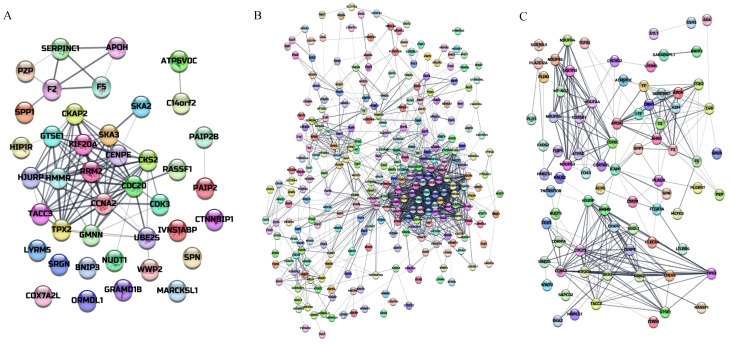
A protein interaction network diagram of differentially expressed proteins of macrophages treated with acetaminophen (APAP) in the presence or absence of polydatin (HU). (**A**) APAP100 vs. control group, (**B**) APAP500 vs. control group, and (**C**) APAP500-HU vs. control group.

Based on the identified differentially expressed proteins, we generated a differential protein interaction network map in which the more lines associated with a protein, the more critical that protein is in the network [39,40]. As shown in Figure 6A, 44 interacting differential proteins were obtained for the low-dose APAP group compared with in the control group, with a total of 110 interactions (*p* < 0.001) in which the largest interaction module included 21 differential proteins. Among the interaction networks, most proteins (17) interacted with the cell division cycle protein 20 homolog, which is encoded by the *Cdc20* gene and is involved in the protein ubiquitination pathway and protein modification.

As shown in Figure 6B, 338 interacting differential proteins were obtained for the high-dose APAP group compared with those in the control group, with a total of 1470 interactions (*p* < 0.001), of which the largest interaction module included 277 differential proteins. Among the interaction networks, the largest number of proteins (54) interacted with cytochrome c oxidase polypeptide IV. This is encoded by the *Cox4i1* gene and is a component of cytochrome c oxidase, the last enzyme of the mitochondrial electron transport chain that drives oxidative phosphorylation.

As shown in Figure 6C, the addition of polydatin after high-dose APAP injury yielded 113 interacting differential proteins compared with that in the control group, with a total of 281 interactions (*p* < 0.001). Among these, the largest interaction module included 76 differential proteins. The largest number of proteins, 17, interacted with cyclin A2, which is encoded by the *Ccna2* gene and is a cellular protein that controls the G1/S and G2/M transition phases of the cell cycle.

### 3.8. Gene Ontology (GO) Analysis

**Figure 7:**
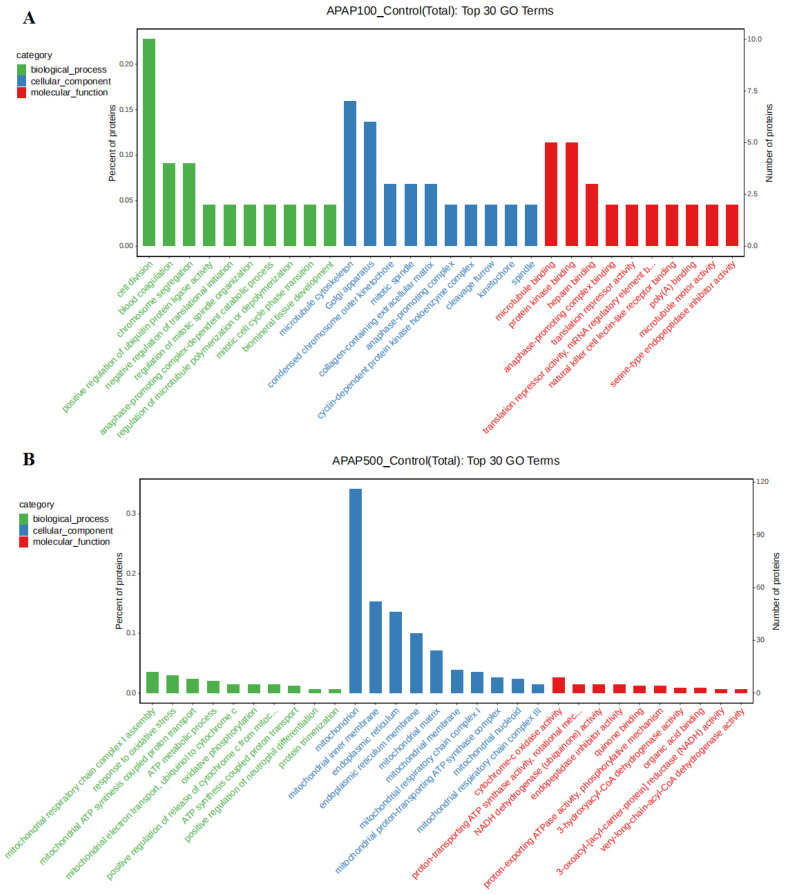
Gene ontology (GO) analysis of differentially expressed proteins of macrophages treated with acetaminophen (APAP). (**A**) APAP100 vs. control group, (**B**) APAP500 vs. control group.

The GO functional classification system was used to analyze differential proteins involved in biological processes, cellular components, and molecular functions in cells at three levels [41] (Figure 7 and Figure S2). The GO analysis revealed that the administration of APAP to cells resulted in cell division, chromosome segregation, acute-phase response, response to oxidative stress, cellular oxidant detoxification, hydrogen peroxide catabolic processes, hydrogen peroxide catabolism, and mitotic cell division. The expression of proteins in the biological processes such as mitotic cell cycle phase transition appeared altered. The cellular components of the differential proteins are mainly mitochondrion, cytoplasm, endoplasmic reticulum, microtubule cytoskeleton, nucleus, nucleolus, and Golgi apparatus (Table S2). The molecular function is mainly enriched in DNA binding, chromatin binding, microtubule binding, endopeptidase inhibitor activity, and serine-type endopeptidase activity. This suggests that the addition of APAP affects apoptosis, division, and oxidative stress functions.

### 3.9. Polydatin Can Repair the Damage Caused by Acetaminophen

**Figure 8:**
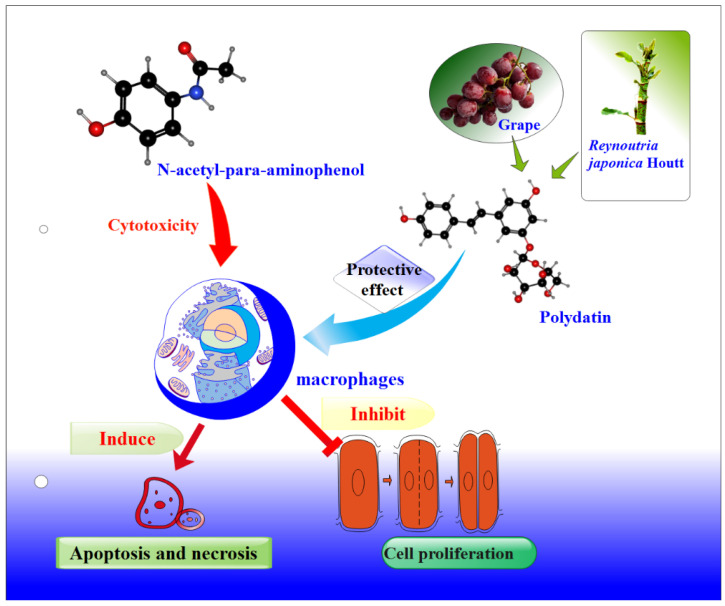
A schematic of the repair of APAP-damaged macrophages by polydatin. Acetaminophen is cytotoxic to macrophages, induces apoptosis, and inhibits cell proliferation, while polydatin, the active ingredient in grapes and *R. japonica*, has the ability to repair damaged cells.

Whether the test cells were damaged was evaluated by analyzing the morphological changes, survival rates, apoptosis, and differential protein expression of macrophages. This showed that culturing macrophages in medium containing APAP caused cell damage. When polydatin was added to the culture medium, a repair effect on macrophages was observed. However, after adding polydatin to the culture medium, microscopic observations of cell morphology revealed that cell differentiation was reduced and cell density increased (Figure 1). The MTT results (Figure S1) showed that the survival rate of the cells increased after adding polydatin to the culture medium, indicating that polydatin can reduce the necrosis and apoptosis rates of macrophages caused by APAP (Figure 2). Based on our results, we propose that polydatin can repair the damage caused by acetaminophen, as illustrated in Figure 8.

## 4. Conclusions

We investigated the effects of APAP on macrophages to determine whether exposure resulted in cell damage. We found that APAP was cytotoxic, promoted macrophage apoptosis, and inhibited cell proliferation. We also determined the mechanism underlying APAP-mediated damage to macrophages using differential protein screening and differential protein bioinformatics analysis. Our proteomics results showed that APAP alters the protein expression profile of macrophages, and these differentially expressed proteins are involved in the immune response, metabolic processes, apoptosis, and acute phase response. APAP has been reported to cause kidney and liver damage, and our work further suggests that exposure to excessive APAP may damage macrophages. At the same time, polydatin could repair macrophage damage caused by APAP, inhibit macrophage differentiation, increase cell density, inhibit cell necrosis and apoptosis, and exert a protective effect on macrophages. Our findings suggest that the potential risk of damage to macrophages in the body in association with the irregular administration of the antipyretic drug APAP should be further considered and that polydatin, the active ingredient in grapes and *R. japonica*, can repair APAP-induced macrophage damage.

## Data Availability

Data available on request.

## Supplementary Materials

The following are available online at https://www.mdpi.com/article/10.3390/nu14102077/s1, Figure S1: MTT assay for cell viability. Effect of acetaminophen (APAP) and polydatin on the survival rate of RAW264.7 cells; Figure S2: Gene ontology classification (ANOVA) analysis of differentially expressed proteins of macrophages treated with acetaminophen (APAP). (A) APAP100 vs. control group, (B) APAP500 vs. control group; Table S1: Differentially expressed proteins of macrophages treated with acetaminophen (APAP) in the presence or absence of polydatin (HU); Table S2: Localization of differentially expressed proteins in macrophages.
