# Supplementary material for: Protective Effects of Polydatin from Grapes and Reynoutria japonica Houtt. on Damaged Macrophages Treated with Acetaminophen
Source: Nutrients. 2022 May 16;14(10):2077. doi: 10.3390/nu14102077 (PMC9147135; doi:10.3390/nu14102077)
Supplement: Supplementary file 1 [file nutrients-14-02077-s001.zip › nutrients-1707909-supplementary.pdf]

## Supporting Information

**Table S1** Differentially expressed proteins of macrophages treated with acetaminophen (APAP) in the presence or absence of polydatin (HU).

**Table S2** Localization of differentially expressed proteins in macrophages

### Figure captions

**Figure S1 MTT assay for cell viability.** Effect of acetaminophen (APAP) and polydatin on the survival rate of RAW264.7 cells.

**Figure S2** Gene ontology classification (ANOVA) analysis of differentially expressed proteins of macrophages treated with acetaminophen (APAP). (A) APAP100 vs control group, (B) APAP500 vs control group.

Table S1

**Table S1** Differentially expressed proteins of macrophages treated with acetaminophen (APAP) or polydatin

| Protein Accession | Gene Name | Control-1 | Control-2 | Control-3 | APAP 100-1 | APAP 100-2 | APAP 100-3 | APAP 500-1 | APAP 500-2 | APAP 500-3 | APAP-HU-1 | APAP-HU-2 | APAP-HU-3 | Adjust pvalue |
|---|---|---|---|---|---|---|---|---|---|---|---|---|---|---|
| Q8BTM8 | Flna | 117.1 | 111.4 | 116 | 98.5 | 103.3 | 103 | 87.3 | 87.2 | 85.9 | 97.4 | 98.1 | 94.6 | 5.15026E-05 |
| E9PVX6 | Mki67 | 110.7 | 106.5 | 108.3 | 130.7 | 126.6 | 128.8 | 61.1 | 59.4 | 63.6 | 98.6 | 109 | 96.7 | 1.70253E-05 |
| P16546 | Sptan1 | 99.8 | 102 | 105.5 | 109.3 | 106.9 | 107.8 | 89.3 | 87.2 | 90.2 | 100.5 | 100.1 | 101.5 | 8.70273E-05 |
| P09103 | P4hb | 98.4 | 103.2 | 104.5 | 86.4 | 92.1 | 91.6 | 116.8 | 116.1 | 113.5 | 92.6 | 90.9 | 94 | 8.47959E-05 |
| P23116 | Eif3a | 105.8 | 103.7 | 107.7 | 106.1 | 106.6 | 106.9 | 87.4 | 84 | 90.4 | 100.7 | 100.2 | 100.2 | 7.48501E-05 |
| Q6P5E4 | Uggt1 | 85.7 | 83.9 | 88.2 | 93.3 | 97.4 | 91.5 | 120.8 | 127.3 | 123.8 | 95.8 | 94.9 | 97.5 | 2.47248E-05 |
| Q8CAQ8 | Immt | 86.1 | 87.8 | 92 | 94.5 | 97.4 | 95.1 | 117.1 | 117.2 | 119.1 | 98.4 | 94 | 101.3 | 6.57115E-05 |
| Q8VHX6 | Flnc | 101.5 | 100 | 101.2 | 104.7 | 106.2 | 106.9 | 87.1 | 88.7 | 88.6 | 105.1 | 104.7 | 105.3 | 5.14414E-06 |
| Q03265 | Atp5f1a | 82.8 | 84 | 85.5 | 97.2 | 96.9 | 91.2 | 120.5 | 123.7 | 120.3 | 101.5 | 96.1 | 100.2 | 2.61375E-05 |
| Q01320 | Top2a | 86.3 | 79.3 | 83.4 | 119.3 | 128.2 | 124.1 | 83.9 | 88.5 | 87.6 | 104 | 111.8 | 103.7 | 6.33677E-05 |
| Q8BFR5 | Tufm | 94 | 92.2 | 96.5 | 90 | 92.3 | 90 | 118.5 | 126.4 | 117.7 | 94.5 | 93.7 | 94.2 | 5.24129E-05 |
| Q91YQ5 | Rpn1 | 90.1 | 86.5 | 90.6 | 97.2 | 99.3 | 93.9 | 112.7 | 119.7 | 116.9 | 97.9 | 96 | 99.3 | 9.6261E-05 |
| Q3U0V1 | Khsrp | 119.3 | 113.6 | 115.8 | 101.9 | 105.1 | 105.4 | 86.8 | 82 | 85.1 | 95.1 | 97.5 | 92.5 | 4.29071E-05 |
| Q8CGK3 | Lonp1 | 91.9 | 86.3 | 90.9 | 88.2 | 94.8 | 88.1 | 125.1 | 130.7 | 124.9 | 92.4 | 93.1 | 93.5 | 2.91578E-05 |
| P49710 | Hcls1 | 99.5 | 98.5 | 98.6 | 122.2 | 111.7 | 116.2 | 76.8 | 70.4 | 80.5 | 108.7 | 107.9 | 109.1 | 7.07414E-05 |
| P26443 | Glud1 | 92.7 | 86.3 | 92.3 | 88.4 | 93.4 | 91.2 | 124.1 | 128.1 | 118.3 | 97.1 | 94.7 | 93.2 | 8.13629E-05 |
| Q9CQN1 | Trap1 | 91.5 | 87.7 | 92.9 | 87.2 | 97.1 | 90.7 | 122.7 | 129.4 | 122.4 | 91.2 | 94.1 | 93.1 | 7.48501E-05 |
| Q7TMK9 | Syncrip | 111.7 | 111.8 | 118.7 | 99 | 101.5 | 100.5 | 88.6 | 89.1 | 88 | 97.9 | 97.1 | 96.1 | 6.46683E-05 |
| Q922J3 | Clip1 | 102.1 | 100.4 | 102.8 | 108.4 | 105.1 | 106.3 | 90.1 | 89.8 | 92.5 | 100.3 | 101.4 | 100.8 | 5.49417E-05 |

| Protein Accession | Gene Name | Control-1 | Control-2 | Control-3 | APAP 100-1 | APAP 100-2 | APAP 100-3 | APAP 500-1 | APAP 500-2 | APAP 500-3 | APAP-HU-1 | APAP-HU-2 | APAP-HU-3 | Adjust pvalue |
|---|---|---|---|---|---|---|---|---|---|---|---|---|---|---|
| Q9QZQ1 | Afdn | 109.9 | 106.6 | 109.6 | 102.6 | 100.8 | 103.6 | 93.3 | 89.9 | 93 | 96 | 98.2 | 96.5 | 9.07385E-05 |
| P30416 | Fkbp4 | 118.6 | 117 | 119 | 97.4 | 100 | 101.2 | 88.4 | 89.9 | 88.3 | 91.2 | 97.3 | 91.6 | 2.76658E-05 |
| P61979 | Hnrnpk | 107 | 112.6 | 110.4 | 113.5 | 105.3 | 106.8 | 79.2 | 77.7 | 82.7 | 101.5 | 103.4 | 99.7 | 8.29845E-05 |
| P18242 | Ctsd | 77.6 | 81.7 | 78.7 | 101.2 | 93.8 | 90.5 | 115.5 | 121.3 | 116.1 | 110.9 | 104.7 | 108.1 | 8.86508E-05 |
| Q8BG05 | Hnrnpa3 | 110.1 | 109.2 | 109.1 | 104.4 | 101.1 | 101.1 | 87.2 | 90 | 93.2 | 97.8 | 99 | 97.7 | 9.92874E-05 |
| Q99MR6 | Srrt | 100.9 | 104.9 | 104.5 | 105.6 | 106.7 | 109.8 | 87.3 | 85.9 | 88.3 | 102.9 | 101.8 | 101.3 | 5.13311E-05 |
| P08249 | Mdh2 | 91 | 89.1 | 87.4 | 85.4 | 86.2 | 89.5 | 131.5 | 133.2 | 122.6 | 93.8 | 94.9 | 95.3 | 2.83047E-05 |
| Q9EQP2 | Ehd4 | 104.4 | 108.8 | 108.7 | 106.7 | 104.2 | 106.1 | 84.8 | 84.9 | 85.7 | 100.7 | 101.6 | 103.4 | 2.56761E-05 |
| Q9Z2I8 | Suclg2 | 86 | 91 | 91.6 | 94.8 | 89.3 | 89.1 | 120.1 | 124.3 | 118.9 | 99.9 | 94.4 | 100.7 | 7.93899E-05 |
| O70318 | Epb41l2 | 105.8 | 106.6 | 107.5 | 103.3 | 103 | 104.4 | 92.4 | 93.4 | 95.5 | 96.2 | 97.2 | 94.7 | 4.82617E-05 |
| Q8VIJ6 | Sfpq | 107.6 | 105.9 | 108.8 | 108.1 | 108.1 | 107.8 | 83.2 | 81 | 86.8 | 101.2 | 99.9 | 101.7 | 2.24605E-05 |
| Q8BHN3 | Ganab | 93.8 | 90.8 | 94.8 | 90.1 | 92.9 | 91.5 | 117.9 | 123 | 115.1 | 95 | 96.6 | 98.5 | 5.91773E-05 |
| Q60597 | Ogdh | 93.4 | 87.2 | 86.7 | 79.4 | 88.1 | 88.3 | 136.9 | 138.3 | 125.9 | 89.3 | 94.8 | 91.5 | 7.46422E-05 |
| Q04447 | Ckb | 111.6 | 118.4 | 117.3 | 98.4 | 97.2 | 100.6 | 87.8 | 91.1 | 90.3 | 93.7 | 97.7 | 95.7 | 7.91683E-05 |
| Q9Z1Z2 | Strap | 111.3 | 110.6 | 110.3 | 100.5 | 100.2 | 98.6 | 89.9 | 94.3 | 90.4 | 96.9 | 100.4 | 96.6 | 7.39442E-05 |
| Q6A068 | Cdc5l | 100.7 | 102.4 | 105.5 | 110.7 | 105.2 | 107.5 | 86.1 | 86 | 87.4 | 103.4 | 103.5 | 101.6 | 7.1825E-05 |
| P10605 | Ctsb | 108.1 | 109.5 | 108.3 | 98.4 | 95.9 | 98.2 | 93.4 | 92.4 | 91.8 | 99.7 | 101.6 | 102.6 | 3.27424E-05 |
| Q99L45 | Eif2s2 | 106.5 | 106.7 | 108.1 | 98.6 | 95.7 | 99.4 | 93.9 | 94.7 | 93 | 102.4 | 100.5 | 100.7 | 9.52528E-05 |
| P30681 | Hmgb2 | 106.8 | 109.2 | 108.6 | 123.7 | 117.7 | 122.2 | 63.8 | 59.5 | 70.3 | 107.5 | 107.9 | 102.9 | 1.96502E-05 |
| P47738 | Aldh2 | 91.1 | 85.4 | 89.8 | 82.9 | 88.1 | 88.9 | 131.1 | 135.5 | 124.7 | 93.1 | 95.2 | 94.2 | 3.49561E-05 |
| Q99LC5 | Etfa | 87.8 | 90.8 | 88.6 | 93.5 | 89.5 | 88.5 | 118.2 | 126.9 | 121.4 | 99.8 | 95.2 | 99.9 | 5.38289E-05 |
| Q8QZT1 | Acat1 | 90.7 | 84.9 | 91.2 | 85.9 | 91.9 | 90.3 | 127.8 | 132.7 | 123.1 | 93.4 | 93.8 | 94.3 | 4.08553E-05 |
| Q8BWT1 | Acaa2 | 90.3 | 86.2 | 92.1 | 84.9 | 87.2 | 87 | 129.8 | 137.9 | 124.2 | 92.4 | 92.4 | 95.7 | 4.8877E-05 |

| Protein Accession | Gene Name | Control-1 | Control-2 | Control-3 | APAP 100-1 | APAP 100-2 | APAP 100-3 | APAP 500-1 | APAP 500-2 | APAP 500-3 | APAP-HU-1 | APAP-HU-2 | APAP-HU-3 | Adjust pvalue |
|---|---|---|---|---|---|---|---|---|---|---|---|---|---|---|
| Q8K1B8 | Fermt3 | 116.4 | 111 | 114.8 | 99.8 | 104.5 | 100.3 | 85 | 86.1 | 85.7 | 96.4 | 101.9 | 98.1 | 6.4123E-05 |
| Q60932 | Vdac1 | 80 | 80.3 | 81.9 | 99.3 | 93.7 | 91.9 | 122.8 | 129 | 120.4 | 103.9 | 94.1 | 102.6 | 8.76837E-05 |
| Q6A028 | Swap70 | 112.4 | 116.7 | 115.3 | 102 | 105 | 101.8 | 85.4 | 84.3 | 87.4 | 94.3 | 99.9 | 95.5 | 4.09455E-05 |
| Q9CVB6 | Arpc2 | 106 | 108.5 | 107.5 | 103.5 | 105 | 100.5 | 88.3 | 88.5 | 90.1 | 99.7 | 102.3 | 100.1 | 5.85888E-05 |
| Q6GQT9 | Nomo1 | 88.3 | 85.3 | 89.9 | 89.8 | 94.4 | 90.7 | 123.5 | 129.8 | 120 | 97.4 | 94.1 | 96.8 | 4.65608E-05 |
| Q91WQ3 | Yars1 | 118.6 | 114.6 | 118.6 | 93.4 | 97.9 | 96.9 | 95.3 | 94.2 | 91.6 | 91.3 | 96.3 | 91.5 | 6.56428E-05 |
| P63158 | Hmgb1 | 105.7 | 109.7 | 107.6 | 116.7 | 110.4 | 116.4 | 75 | 66.6 | 76.5 | 107.7 | 105.7 | 102 | 5.43714E-05 |
| Q8QZY1 | Eif3l | 108.8 | 105.8 | 108.3 | 101 | 103.2 | 102.5 | 90.6 | 92.2 | 89.7 | 99.7 | 100.2 | 98 | 4.47401E-05 |
| P17918 | Pcna | 116.1 | 119.2 | 112.9 | 105 | 102.9 | 103.1 | 81.2 | 82.4 | 80.9 | 98.9 | 101.6 | 95.8 | 2.58974E-05 |
| Q7TSC1 | Prrc2a | 95.3 | 95.1 | 94 | 115 | 109 | 110.4 | 87.3 | 83.3 | 89.9 | 108.7 | 105.2 | 107 | 8.44592E-05 |
| P05202 | Got2 | 88.9 | 88.6 | 83.6 | 75.5 | 81.1 | 82.5 | 129.8 | 140.8 | 126.7 | 102.7 | 95.9 | 103.9 | 7.15723E-05 |
| Q99K85 | Psat1 | 123 | 122.4 | 122.1 | 99.2 | 98.1 | 98.8 | 83.1 | 86.3 | 83.1 | 94.4 | 96 | 93.4 | 1.9039E-06 |
| P54071 | Idh2 | 98 | 99.3 | 96.7 | 87.5 | 87.6 | 89.7 | 118.5 | 127.2 | 117.7 | 92.4 | 92.3 | 93.1 | 4.8877E-05 |
| P67778 | Phb | 82.5 | 84.4 | 85.1 | 100.1 | 95.5 | 94.4 | 115.5 | 118.2 | 118.6 | 102.5 | 97.2 | 105.8 | 6.7414E-05 |
| P68510 | Ywhah | 111.7 | 113.7 | 114.2 | 98.6 | 101.8 | 102.1 | 88.8 | 85.4 | 86.1 | 99 | 101.6 | 97 | 3.52341E-05 |
| P62814 | Atp6v1b 2 | 99.6 | 99.6 | 99.7 | 90.4 | 91.8 | 93.1 | 113.5 | 114.8 | 108.4 | 96.9 | 96.4 | 95.8 | 7.15723E-05 |
| Q9CZU6 | Cs | 92.5 | 89.1 | 92.2 | 91.2 | 93.2 | 93.9 | 118.3 | 124.5 | 118.7 | 94.2 | 94.7 | 97.6 | 2.96388E-05 |
| P51881 | Slc25a5 | 85.5 | 81.7 | 79.8 | 91.2 | 91.1 | 89.2 | 124.3 | 134.2 | 126.7 | 99.1 | 97.4 | 99.8 | 2.38593E-05 |
| Q9R1T2 | Sae1 | 116.4 | 117 | 120 | 97.3 | 99.4 | 100.4 | 88.3 | 90.3 | 85.7 | 94.7 | 97.4 | 92.9 | 2.76301E-05 |
| P11157 | Rrm2 | 69.3 | 71 | 69.1 | 119.2 | 109.9 | 114.8 | 96.1 | 99 | 95.8 | 121.5 | 118.6 | 115.7 | 1.70253E-05 |
| P21550 | Eno3 | 119 | 118 | 123.9 | 98.4 | 101.6 | 104.3 | 85.5 | 84.7 | 82.4 | 94.1 | 94.8 | 93.2 | 2.38593E-05 |
| Q8BH59 | Slc25a12 | 90.4 | 87.9 | 90.5 | 85.8 | 91.1 | 87.4 | 118.8 | 131.1 | 122.5 | 97.9 | 97.9 | 98.6 | 7.24382E-05 |

| Protein Accession | Gene Name | Control-1 | Control-2 | Control-3 | APAP 100-1 | APAP 100-2 | APAP 100-3 | APAP 500-1 | APAP 500-2 | APAP 500-3 | APAP-HU-1 | APAP-HU-2 | APAP-HU-3 | Adjust pvalue |
|---|---|---|---|---|---|---|---|---|---|---|---|---|---|---|
| Q99L47 | St13 | 105.9 | 110.4 | 111.7 | 107.6 | 103.6 | 105.6 | 84 | 81.8 | 85.3 | 102.2 | 100.4 | 101.4 | 4.29071E-05 |
| O35857 | Timm44 | 85.3 | 88.7 | 90.5 | 94 | 94.2 | 91.3 | 118.7 | 122.7 | 121.6 | 99.3 | 96.1 | 97.8 | 1.81807E-05 |
| Q8BH04 | Pck2 | 102.4 | 103.3 | 103.3 | 83.1 | 88.4 | 88.1 | 118.8 | 123.2 | 116.3 | 87.8 | 93.4 | 91.8 | 4.47401E-05 |
| P50544 | Acadvl | 74.5 | 73.5 | 78.4 | 85.4 | 89.6 | 85.3 | 142.9 | 147.3 | 139.9 | 93.7 | 94.5 | 95.2 | 2.35904E-06 |
| Q8BK64 | Ahsa1 | 102.3 | 102.8 | 101.1 | 107.2 | 106.4 | 102.4 | 89.4 | 89.3 | 89.3 | 104 | 104.6 | 101.2 | 9.57349E-05 |
| Q9WVA4 | Tagln2 | 112 | 117.3 | 116 | 107.6 | 100.6 | 106.6 | 82 | 76.4 | 77.1 | 105.3 | 100 | 99.1 | 7.24382E-05 |
| Q91VH2 | Snx9 | 120.4 | 118.4 | 118.4 | 89.1 | 97.9 | 96.3 | 94.1 | 93.6 | 91.9 | 92.3 | 95.6 | 92.1 | 7.15723E-05 |
| Q922U1 | Prpf3 | 100.7 | 100.8 | 102.6 | 110.4 | 108.7 | 107.9 | 88.3 | 85 | 88 | 100.5 | 103.4 | 103.8 | 2.95731E-05 |
| P25976 | Ubtf | 106.5 | 109.9 | 107.7 | 103.8 | 101.7 | 102.9 | 90.1 | 89.8 | 91.1 | 99.6 | 99.8 | 97.1 | 3.65166E-05 |
| Q8R180 | Ero1a | 103 | 100.7 | 100.7 | 78.5 | 84.5 | 86.7 | 127.7 | 130.8 | 119.2 | 87.9 | 90.8 | 89.5 | 6.20583E-05 |
| Q60668 | Hnrnpd | 117.6 | 121.9 | 113.2 | 101 | 100.8 | 98.7 | 84.5 | 83.1 | 86 | 97.3 | 100.2 | 95.8 | 5.15026E-05 |
| D3Z7P3 | Gls | 87.4 | 86.3 | 90.2 | 83.3 | 87.6 | 87 | 128.6 | 140.9 | 131.7 | 90.6 | 94 | 92.4 | 2.76658E-05 |
| Q9DCN2 | Cyb5r3 | 83.1 | 86.3 | 84.6 | 90.7 | 85.7 | 87.9 | 128.9 | 138.3 | 126.6 | 96.1 | 96.1 | 95.8 | 2.76658E-05 |
| Q8BI84 | Mia3 | 86.9 | 86.6 | 87.4 | 104.2 | 98.4 | 99.8 | 108.3 | 109.6 | 109.4 | 103.7 | 101.7 | 103.8 | 4.06613E-05 |
| Q6A065 | Cep170 | 108.5 | 107.1 | 105.5 | 114.6 | 107.8 | 111.3 | 80.9 | 76.5 | 80.2 | 100 | 105.9 | 101.6 | 4.61936E-05 |
| O35841 | Api5 | 119.6 | 118.9 | 124.1 | 93.6 | 99.7 | 100.3 | 90.5 | 86.9 | 84.6 | 91.3 | 97.4 | 93 | 8.24006E-05 |
| Q60865 | Caprin1 | 107.5 | 111.1 | 108.2 | 117.5 | 107.4 | 113.9 | 75 | 70.8 | 75.8 | 108.6 | 103 | 101.3 | 6.21969E-05 |
| P48722 | Hspa4l | 93.4 | 95.5 | 92.9 | 87.2 | 92.3 | 94 | 124.6 | 120.6 | 116.3 | 92.1 | 96.5 | 94.7 | 8.51028E-05 |
| Q07076 | Anxa7 | 91.1 | 92.6 | 92.2 | 94.3 | 92.5 | 93.8 | 109.8 | 115.6 | 111.3 | 102.7 | 100.5 | 103.5 | 5.07121E-05 |
| Q6P9P6 | Kif11 | 82.6 | 79.2 | 83.3 | 109.5 | 113.9 | 113.7 | 93.5 | 100.8 | 98.4 | 105.6 | 111.3 | 108.3 | 7.07414E-05 |
| Q925I1 | Atad3 | 89.7 | 90.9 | 92.9 | 95.3 | 95.3 | 90.8 | 114.9 | 118.7 | 116.8 | 100.9 | 94.4 | 99.4 | 7.51165E-05 |
| P29758 | Oat | 93.8 | 91.8 | 97.4 | 90.9 | 92.5 | 91.2 | 121.5 | 129.9 | 118.6 | 89.6 | 92.2 | 90.6 | 7.55784E-05 |
| Q8K297 | Colgalt1 | 87.9 | 87.6 | 91.3 | 91.9 | 92 | 88.8 | 119.8 | 131.9 | 122.7 | 96.5 | 94.5 | 95.2 | 6.94212E-05 |

| Protein Accession | Gene Name | Control-1 | Control-2 | Control-3 | APAP 100-1 | APAP 100-2 | APAP 100-3 | APAP 500-1 | APAP 500-2 | APAP 500-3 | APAP-HU-1 | APAP-HU-2 | APAP-HU-3 | Adjust pvalue |
|---|---|---|---|---|---|---|---|---|---|---|---|---|---|---|
| Q810A7 | Ddx42 | 100.8 | 100.9 | 103.5 | 111.4 | 106.3 | 105.8 | 84.7 | 83.8 | 86.5 | 106.3 | 104.6 | 105.3 | 4.47401E-05 |
| P10711 | Tcea1 | 112.6 | 110.5 | 115.9 | 101.5 | 100.4 | 103.4 | 88.3 | 84.4 | 85.8 | 96 | 102.2 | 99 | 8.29845E-05 |
| Q8C1B7 | Septin11 | 107 | 108 | 111.2 | 99.4 | 100.6 | 101.8 | 92.3 | 91.7 | 89.8 | 99 | 101 | 98 | 9.3097E-05 |
| Q8K298 | Anln | 88.3 | 86.8 | 85.5 | 127.4 | 123.3 | 130.5 | 70.5 | 75.2 | 76.9 | 109.3 | 117.1 | 109.1 | 1.96502E-05 |
| Q91VX2 | Ubap2 | 102.5 | 100.4 | 100.8 | 124.9 | 110.6 | 119.3 | 77.2 | 72.2 | 78.1 | 104.4 | 105.8 | 103.9 | 9.65963E-05 |
| Q8BNW9 | Kbtbd11 | 115.9 | 117.4 | 110.2 | 85.1 | 84.8 | 89.2 | 104.8 | 105.7 | 103.4 | 92.3 | 94.3 | 96.8 | 7.33727E-05 |
| O08808 | Diaph1 | 109.4 | 109.6 | 112.7 | 100.6 | 100.3 | 101.2 | 90 | 91.1 | 92.3 | 97.2 | 98.3 | 97.3 | 1.96502E-05 |
| Q9DCX2 | Atp5pd | 79.5 | 82.3 | 81.5 | 101.2 | 96 | 95 | 119 | 119.3 | 118.3 | 105.3 | 97.7 | 105 | 4.19587E-05 |
| Q99KQ4 | Nampt | 113 | 110.2 | 113.7 | 93.2 | 96.2 | 96.9 | 96.8 | 97.1 | 93.3 | 95.7 | 98 | 96 | 8.86508E-05 |
| Q99JY0 | Hadhb | 82.9 | 86 | 85 | 100 | 95.9 | 91.2 | 117.8 | 123.2 | 121.7 | 98.8 | 97.3 | 100.1 | 4.47401E-05 |
| Q922Q8 | Lrrc59 | 108.9 | 112.3 | 112.2 | 98.6 | 95.8 | 100.3 | 95.1 | 96.7 | 96.2 | 94.2 | 95 | 94.7 | 6.73372E-05 |
| P46664 | Adss2 | 122.3 | 116.9 | 120.7 | 94.8 | 101.6 | 101.1 | 86.1 | 88.4 | 84.6 | 93.4 | 96.6 | 93.6 | 4.63182E-05 |
| P11440 | Cdk1 | 94 | 102.3 | 101.7 | 125.4 | 117.8 | 116 | 71.5 | 73.4 | 76.5 | 108.9 | 105.9 | 106.8 | 4.9195E-05 |
| Q9D051 | Pdhb | 88.3 | 88.4 | 93.3 | 91.3 | 91.2 | 91.8 | 120.7 | 127.4 | 118.6 | 95 | 94 | 99.8 | 6.87947E-05 |
| Q5SSZ5 | Tns3 | 115.6 | 111.1 | 114.7 | 99.3 | 99.3 | 99.1 | 85.7 | 91 | 87.1 | 97.8 | 100.5 | 98.9 | 4.29071E-05 |
| Q9CQW2 | Arl8b | 94.4 | 95.6 | 96.4 | 94 | 93 | 91.6 | 114.3 | 120.5 | 113.1 | 93.8 | 95.9 | 97.4 | 7.93899E-05 |
| A2APB8 | Tpx2 | 81.1 | 74.8 | 76.4 | 130.8 | 132.7 | 133.5 | 69.1 | 68.6 | 70.9 | 118.7 | 127.3 | 116 | 8.54234E-06 |
| Q5SXY1 | Specc1 | 109.2 | 106.3 | 105.6 | 105.7 | 102.7 | 107.6 | 86.3 | 86.4 | 86.4 | 99 | 103.8 | 101 | 7.15068E-05 |
| P51174 | Acadl | 86.8 | 83.1 | 85.9 | 91 | 95.3 | 91 | 120.4 | 134.1 | 127.4 | 93.3 | 96.4 | 95.3 | 6.7414E-05 |
| Q9ESZ8 | Gtf2i | 111.6 | 116.5 | 116.4 | 101.6 | 101.7 | 100.5 | 86.8 | 88.6 | 89.5 | 93.9 | 97 | 96 | 2.61375E-05 |
| P23198 | Cbx3 | 103.9 | 102.9 | 107.7 | 108.1 | 108.5 | 108.6 | 85 | 83.4 | 87.1 | 103 | 103.3 | 98.5 | 6.20315E-05 |
| Q9DCW4 | Etfb | 89.1 | 90.8 | 94.5 | 90.6 | 91.8 | 91.7 | 120.9 | 124 | 119.1 | 95.7 | 94.5 | 97.3 | 1.83092E-05 |
| Q9D0F3 | Lman1 | 86 | 86.5 | 89.3 | 90 | 94 | 91 | 118.6 | 128.8 | 118.8 | 100.4 | 95.9 | 100.7 | 9.52528E-05 |

| Protein Accession | Gene Name | Control-1 | Control-2 | Control-3 | APAP 100-1 | APAP 100-2 | APAP 100-3 | APAP 500-1 | APAP 500-2 | APAP 500-3 | APAP-HU-1 | APAP-HU-2 | APAP-HU-3 | Adjust pvalue |
|---|---|---|---|---|---|---|---|---|---|---|---|---|---|---|
| Q6NZF1 | Zc3h11a | 95.5 | 90.3 | 91.9 | 116.5 | 112.4 | 110.3 | 89.4 | 87.6 | 89.6 | 105.7 | 107.3 | 103.3 | 7.15723E-05 |
| P10404 | | 91.5 | 90.7 | 87.2 | 87.6 | 89.3 | 93.3 | 123.5 | 124.6 | 115.4 | 101.2 | 99.2 | 96.5 | 9.71942E-05 |
| O08795 | Prkcsh | 82.2 | 85.6 | 87.5 | 93.5 | 92.2 | 92.4 | 121.5 | 125.4 | 120.7 | 100.8 | 96.9 | 101.3 | 1.76997E-05 |
| Q9Z2I9 | Sucla2 | 93.1 | 94.5 | 91.4 | 87.8 | 88.1 | 87.3 | 119 | 131.7 | 121.9 | 94.4 | 93.4 | 97.4 | 8.20987E-05 |
| Q9DBH5 | Lman2 | 89.1 | 90.3 | 93.6 | 92.6 | 95.2 | 90.9 | 114.4 | 120.3 | 116.2 | 100.3 | 96.2 | 100.8 | 7.48501E-05 |
| Q3TIV5 | Zc3h15 | 107.2 | 112.2 | 114.1 | 105.7 | 101.4 | 106.5 | 87.1 | 84.1 | 84.5 | 99.2 | 98.9 | 99 | 8.09981E-05 |
| P98078 | Dab2 | 102.1 | 105.4 | 98.3 | 131.9 | 116.6 | 133.1 | 58.9 | 55.3 | 61.8 | 114.1 | 111.3 | 111.2 | 4.05129E-05 |
| Q8BVE3 | Atp6v1h | 93.8 | 88.9 | 93 | 89.6 | 94.8 | 92.3 | 117.7 | 122.4 | 116.9 | 95.7 | 98 | 97 | 4.82617E-05 |
| Q9DC61 | Pmpca | 93.1 | 94.5 | 97.8 | 88 | 93.4 | 89.4 | 118.5 | 125.8 | 118.4 | 94 | 91.2 | 95.9 | 8.02617E-05 |
| Q60930 | Vdac2 | 93 | 91.3 | 89 | 85.1 | 86.3 | 89.1 | 127.5 | 131.2 | 118.8 | 95.2 | 96 | 97.3 | 5.97094E-05 |
| Q9CYN2 | Spcs2 | 89.3 | 90.1 | 92.9 | 95.5 | 95.4 | 96.2 | 113.1 | 114.9 | 110.6 | 101.9 | 97.4 | 102.6 | 7.45517E-05 |
| P32020 | Scp2 | 100.6 | 100.4 | 101.7 | 105.7 | 104.5 | 105.8 | 92.5 | 87.8 | 91.8 | 102.8 | 102.6 | 103.6 | 7.8241E-05 |
| Q8C129 | Lnpep | 83.1 | 88.5 | 87.7 | 95.5 | 95.5 | 90.2 | 122.7 | 120.9 | 117.4 | 101.4 | 97.6 | 99.6 | 4.39456E-05 |
| Q6PGB6 | Naa50 | 106.9 | 111.4 | 108.2 | 102.1 | 100.2 | 101.1 | 88.6 | 88.1 | 86.7 | 103.5 | 101.3 | 101.9 | 2.95731E-05 |
| P35979 | Rpl12 | 103.8 | 105 | 103.6 | 99.8 | 102.3 | 99.9 | 93.5 | 94.6 | 95.2 | 100.5 | 101 | 100.7 | 9.92272E-05 |
| Q3UKJ7 | Smu1 | 109.6 | 107 | 106 | 101.4 | 99.6 | 100.1 | 91.4 | 92.4 | 90 | 100.2 | 101 | 101.4 | 4.47401E-05 |
| P59328 | Wdhd1 | 108.1 | 104.3 | 102.4 | 126.7 | 120.3 | 123.2 | 69.6 | 70.9 | 71.4 | 98 | 108.5 | 96.6 | 4.08553E-05 |
| Q60715 | P4ha1 | 112.7 | 114 | 115.7 | 100.8 | 102 | 103.4 | 88.3 | 92.6 | 90.5 | 92.3 | 93.4 | 94.2 | 2.0176E-05 |
| O35639 | Anxa3 | 78.2 | 82.7 | 79.7 | 95.3 | 91.9 | 93.4 | 114.1 | 118.8 | 114.2 | 109.7 | 109.3 | 112.7 | 1.70253E-05 |
| P39749 | Fen1 | 104.6 | 109.3 | 108 | 105.4 | 103.5 | 110.4 | 84.6 | 84.8 | 84.1 | 103 | 101.8 | 100.5 | 7.33727E-05 |
| Q8VEH3 | Arl8a | 82.8 | 83.7 | 89.2 | 94.4 | 91.3 | 90.6 | 118.3 | 127.1 | 121 | 99.1 | 98.6 | 103.8 | 7.00366E-05 |
| Q9Z130 | Hnrnpdl | 111.3 | 112.5 | 110.6 | 106.9 | 100.9 | 104.3 | 84.5 | 79 | 80.8 | 104.9 | 102.1 | 102.3 | 3.57173E-05 |
| Q9JJU8 | Sh3bgrl | 108.2 | 109.4 | 109.9 | 104.5 | 104 | 103.4 | 84.6 | 83.3 | 84.2 | 104.3 | 102.2 | 102 | 1.9039E-06 |

| Protein Accession | Gene Name | Control-1 | Control-2 | Control-3 | APAP 100-1 | APAP 100-2 | APAP 100-3 | APAP 500-1 | APAP 500-2 | APAP 500-3 | APAP-HU-1 | APAP-HU-2 | APAP-HU-3 | Adjust pvalue |
|---|---|---|---|---|---|---|---|---|---|---|---|---|---|---|
| P59325 | Eif5 | 110.6 | 113.3 | 112.9 | 93.8 | 92 | 95.9 | 99.9 | 100.2 | 96.5 | 94.7 | 96.2 | 94 | 5.97094E-05 |
| P97377 | Cdk2 | 93.4 | 96.4 | 99.2 | 115.8 | 108.9 | 111.5 | 85.8 | 86.1 | 85.7 | 106.2 | 104.7 | 106.2 | 6.24958E-05 |
| Q00547 | Hmmr | 68.8 | 67.7 | 72.2 | 122.2 | 121.1 | 125.4 | 85.7 | 92.2 | 91.1 | 116.6 | 120.1 | 116.8 | 5.33395E-06 |
| Q920E5 | Fdps | 139.6 | 137.3 | 136.3 | 91.5 | 103 | 106.5 | 73.5 | 72.2 | 68.8 | 89.9 | 94.2 | 87.1 | 2.89431E-05 |
| Q9EQH2 | Erap1 | 91.1 | 92.3 | 92.1 | 93.7 | 97 | 92 | 115.8 | 120.7 | 113.1 | 98.2 | 97.9 | 96.1 | 7.39442E-05 |
| Q8R5A3 | Apbb1ip | 113.5 | 114.6 | 112.2 | 102.2 | 99.5 | 107.4 | 82.7 | 81.5 | 83.5 | 102.2 | 100.7 | 99.9 | 3.57134E-05 |
| Q9D2G2 | Dlst | 86.3 | 86.5 | 87.9 | 98.3 | 93.9 | 95 | 116.9 | 112.4 | 117.2 | 105.2 | 97.7 | 102.8 | 8.45019E-05 |
| P28650 | Adss1 | 119.3 | 115.9 | 122.6 | 93.3 | 95.6 | 97.3 | 91.6 | 94.4 | 89.3 | 92.7 | 95.7 | 92.4 | 5.15026E-05 |
| Q61941 | Nnt | 78.5 | 75.3 | 80.6 | 99.3 | 100.7 | 94.6 | 117.8 | 124.7 | 123 | 101.8 | 98.5 | 105.2 | 4.08553E-05 |
| Q8VEM8 | Slc25a3 | 87 | 83.4 | 86.6 | 89.2 | 92.4 | 88.4 | 127.4 | 132.8 | 124.3 | 96.5 | 95.6 | 96.3 | 1.756E-05 |
| Q922Q4 | Pycr2 | 87.7 | 88.3 | 87.2 | 94 | 93.4 | 94.9 | 115.8 | 123 | 117 | 100.4 | 97.3 | 100.9 | 2.96388E-05 |
| P45952 | Acadm | 85.7 | 82.8 | 87.9 | 90.1 | 91.7 | 92.7 | 120.8 | 129.5 | 123.5 | 99.2 | 95 | 101.2 | 4.06613E-05 |
| P51863 | Atp6v0d1 | 93.5 | 95.6 | 85.3 | 82.9 | 88.1 | 87.3 | 126.1 | 130.5 | 126.1 | 94.1 | 96.6 | 93.8 | 4.19587E-05 |
| O09159 | Man2b1 | 75.1 | 79.9 | 78.7 | 92.1 | 93.4 | 87.6 | 129.7 | 136 | 131 | 100.2 | 97.5 | 98.8 | 9.56192E-06 |
| Q60931 | Vdac3 | 85.4 | 84 | 80.5 | 96 | 88.2 | 90.1 | 121 | 128.9 | 122.4 | 103.9 | 97.7 | 101.9 | 6.20531E-05 |
| Q9CR68 | Uqcrfs1 | 80.8 | 81 | 81.7 | 99.5 | 99.9 | 92.6 | 116.7 | 125.6 | 125.4 | 99.1 | 95.9 | 101.8 | 7.2363E-05 |
| Q80U78 | Pum1 | 110.6 | 107.8 | 110.5 | 104.7 | 102.2 | 103.6 | 87.6 | 88.8 | 92.5 | 96.7 | 98.7 | 96.4 | 6.42423E-05 |
| Q9D1J3 | Sarnp | 108.3 | 104.5 | 108.2 | 114.2 | 109.7 | 114.3 | 81.4 | 70.7 | 80.9 | 101.5 | 103.2 | 103.1 | 9.68381E-05 |
| Q9QYG0 | Ndrg2 | 116.1 | 115.7 | 121.6 | 97.8 | 99 | 99 | 89.1 | 91.8 | 87.7 | 93.1 | 95.9 | 93.2 | 3.47152E-05 |
| Q9DCS9 | Ndufb10 | 79.7 | 82.3 | 81.1 | 102.8 | 94.4 | 95.2 | 119.2 | 117.9 | 118.6 | 105.2 | 98.9 | 104.7 | 5.12469E-05 |
| P35486 | Pdha1 | 91 | 86.4 | 92.8 | 88.4 | 90.9 | 91.5 | 123.2 | 129.3 | 122.4 | 93.1 | 95.7 | 95.3 | 2.83047E-05 |
| Q921G7 | Etfdh | 87.2 | 80.1 | 83.2 | 84.9 | 90.8 | 88.5 | 128.8 | 140.4 | 129.4 | 92.7 | 97.9 | 96.1 | 4.63182E-05 |

| Protein Accession | Gene Name | Control-1 | Control-2 | Control-3 | APAP 100-1 | APAP 100-2 | APAP 100-3 | APAP 500-1 | APAP 500-2 | APAP 500-3 | APAP-HU-1 | APAP-HU-2 | APAP-HU-3 | Adjust pvalue |
|---|---|---|---|---|---|---|---|---|---|---|---|---|---|---|
| Q91WN1 | Dnajc9 | 107.2 | 108.9 | 112.9 | 114.7 | 110.3 | 116.7 | 74.7 | 74.6 | 77.4 | 99.5 | 102.6 | 100.4 | 1.96502E-05 |
| Q6RT24 | Cenpe | 71.5 | 69.9 | 68.8 | 127.4 | 118.5 | 120.4 | 85.5 | 91.7 | 97.9 | 118.5 | 116.1 | 113.7 | 3.49561E-05 |
| P35293 | Rab18 | 90 | 93.4 | 92.4 | 97.1 | 98.1 | 94.9 | 111.7 | 111.4 | 110.9 | 99.3 | 98.1 | 102.6 | 6.42423E-05 |
| Q8JZQ2 | Afg3l2 | 91.4 | 86.1 | 92.4 | 86.9 | 93.4 | 89.6 | 125.2 | 130.2 | 125.2 | 92.5 | 92.8 | 94.2 | 2.53608E-05 |
| P19536 | Cox5b | 77.3 | 78.6 | 80.9 | 98.1 | 98 | 92.5 | 119.3 | 124.8 | 129.2 | 101.8 | 96.8 | 102.9 | 4.54763E-05 |
| P31266 | Rbpj | 129.5 | 130.6 | 131.4 | 90.9 | 97.4 | 95.9 | 83.8 | 83.9 | 80.8 | 90.4 | 93.6 | 91.7 | 5.14414E-06 |
| Q62087 | Pon3 | 89.5 | 88.2 | 90.6 | 95.4 | 95.1 | 94 | 115.7 | 120.6 | 112.5 | 99.7 | 98.2 | 100.6 | 5.12469E-05 |
| Q61102 | Abcb7 | 85.9 | 91.4 | 89.5 | 95.5 | 96.8 | 91.9 | 119.9 | 119.8 | 116.9 | 97.9 | 95.1 | 99.4 | 4.06613E-05 |
| P52293 | Kpna2 | 86.2 | 83.7 | 87.8 | 124.1 | 128.7 | 128 | 73.1 | 75.7 | 75.2 | 109.2 | 115.7 | 112.4 | 4.96362E-06 |
| P97329 | Kif20a | 75.4 | 73.3 | 74.3 | 110.1 | 116.8 | 118.9 | 88.5 | 98.6 | 92 | 113.5 | 121.3 | 117.3 | 5.4329E-05 |
| Q9JKB3 | Ybx3 | 113.7 | 113.4 | 111.1 | 115.9 | 107 | 110.1 | 75.7 | 71.9 | 81.6 | 99.8 | 101.3 | 98.6 | 6.88347E-05 |
| O08756 | Hsd17b10 | 86.7 | 89.8 | 85.9 | 96.4 | 88.4 | 89.7 | 121.7 | 123.7 | 120 | 98.2 | 97.2 | 102.2 | 4.63182E-05 |
| Q9EP89 | Lactb | 82.3 | 75.1 | 82.3 | 87.6 | 92.1 | 88.9 | 134 | 142.9 | 133.7 | 92.1 | 93.7 | 95.4 | 1.96502E-05 |
| Q91ZA3 | Pcca | 93.5 | 88.4 | 94 | 87 | 89.5 | 86.6 | 125 | 132.4 | 123.2 | 94.5 | 93.3 | 92.6 | 3.09919E-05 |
| Q80TM9 | Nisch | 76.9 | 76 | 78.1 | 93.1 | 96.3 | 92.2 | 118 | 129.7 | 127.1 | 101.7 | 103.5 | 107.3 | 4.07834E-05 |
| Q8C7X2 | Emc1 | 82.2 | 82.8 | 86.8 | 95.6 | 95 | 91.7 | 118.5 | 126 | 119 | 103.6 | 96.7 | 102.1 | 6.50838E-05 |
| Q99MN9 | Pccb | 91.9 | 89.4 | 85.2 | 82.9 | 87.8 | 90.1 | 127.9 | 134.7 | 124.8 | 94.2 | 95.2 | 96 | 4.19898E-05 |
| Q3U0V2 | Tradd | 90.9 | 91.4 | 92.3 | 85.9 | 90.2 | 88.4 | 121.6 | 133 | 124.3 | 92.1 | 96 | 94 | 4.65824E-05 |
| Q07417 | Acads | 86.9 | 85.2 | 90.1 | 85.4 | 88.9 | 89.4 | 130.5 | 130.5 | 123.4 | 96.2 | 95.2 | 98.3 | 1.96502E-05 |
| Q922V4 | Plrg1 | 100.9 | 103.1 | 99.1 | 108.5 | 106.7 | 105.4 | 91.3 | 87.5 | 88.4 | 103.6 | 103.4 | 102.1 | 7.93899E-05 |
| Q99LP6 | Grpel1 | 81.9 | 85.9 | 86.6 | 94.1 | 95.1 | 93.8 | 120.7 | 122.1 | 121.9 | 100.2 | 95.7 | 102.2 | 1.76997E-05 |
| P20108 | Prdx3 | 82.2 | 82.9 | 81.4 | 96.5 | 91.5 | 89.4 | 118.5 | 131.4 | 126.7 | 101.7 | 97.2 | 100.7 | 7.39442E-05 |

| Protein Accession | Gene Name | Control-1 | Control-2 | Control-3 | APAP 100-1 | APAP 100-2 | APAP 100-3 | APAP 500-1 | APAP 500-2 | APAP 500-3 | APAP-HU-1 | APAP-HU-2 | APAP-HU-3 | Adjust pvalue |
|---|---|---|---|---|---|---|---|---|---|---|---|---|---|---|
| Q99P31 | Hspbp1 | 120.3 | 122.1 | 116 | 95.6 | 98.7 | 101 | 84.9 | 84.8 | 82.8 | 97 | 100.2 | 96.5 | 2.87326E-05 |
| Q9JLN9 | Mtor | 97.4 | 91.2 | 98.7 | 88.6 | 92.6 | 92 | 117.2 | 119.8 | 118.9 | 92 | 95.8 | 95.7 | 6.85537E-05 |
| Q6ZQM8 | Ugt1a7 | 88.5 | 88 | 91.5 | 91.2 | 88.9 | 87.6 | 123 | 131.8 | 122.9 | 96 | 94.6 | 95.8 | 2.95731E-05 |
| P09671 | Sod2 | 79.3 | 75.8 | 76 | 98.6 | 88.6 | 90.2 | 118.2 | 128.3 | 129 | 107.2 | 103.8 | 105 | 7.00366E-05 |
| P42125 | Eci1 | 87.2 | 84.9 | 92.3 | 91.2 | 94.4 | 90.1 | 120.1 | 126.7 | 126.4 | 94.2 | 94 | 98.7 | 4.92243E-05 |
| Q9DB20 | Atp5po | 82.6 | 85.1 | 84.3 | 98.6 | 96.9 | 93.6 | 120.3 | 116.1 | 118 | 103.9 | 96.2 | 104.3 | 6.46683E-05 |
| P48024 | Eif1 | 89 | 91.4 | 92.4 | 110.6 | 111.8 | 109.5 | 84.6 | 86.5 | 88.9 | 114 | 110.7 | 110.6 | 1.76997E-05 |
| P33174 | Kif4 | 99.6 | 94.2 | 98.7 | 109.9 | 111.6 | 114.4 | 85.7 | 88.1 | 86.9 | 104.4 | 104.6 | 101.8 | 5.67837E-05 |
| Q8K2C9 | Hacd3 | 81.1 | 77.4 | 84.7 | 86.3 | 93.8 | 85.6 | 130.1 | 142 | 135.2 | 93.4 | 95.3 | 95.2 | 3.4792E-05 |
| Q60787 | Lcp2 | 104.7 | 115.7 | 106.6 | 124.6 | 115.5 | 119.3 | 59.2 | 59.1 | 61.6 | 112.2 | 113 | 108.5 | 2.16614E-05 |
| Q9DCE5 | Pak1ip1 | 115.2 | 114.7 | 116.9 | 97.3 | 98.3 | 100.6 | 88.4 | 89.1 | 87.1 | 94.5 | 100.9 | 97.3 | 3.49561E-05 |
| P99029 | Prdx5 | 85.3 | 86.9 | 89.7 | 92.2 | 93.7 | 91.7 | 121.8 | 119.2 | 117.5 | 101 | 99.9 | 101.2 | 1.10006E-05 |
| P38060 | Hmgcl | 95 | 94.9 | 94.7 | 93.3 | 88.1 | 91.1 | 117.9 | 122.6 | 113.8 | 95.3 | 96.1 | 97.1 | 7.31812E-05 |
| Q8CGC6 | Rbm28 | 98.3 | 93.1 | 94.9 | 89.9 | 93.5 | 92.4 | 114.7 | 119 | 116.8 | 94 | 97.1 | 96.5 | 4.47401E-05 |
| D0QMC3 | Mndal | 100.5 | 102 | 103.4 | 116.8 | 112.7 | 114.6 | 83.2 | 81.9 | 80.8 | 96.9 | 106.6 | 100.7 | 7.00366E-05 |
| Q8K268 | Abcf3 | 88.2 | 84.8 | 91.8 | 93.2 | 94.4 | 94.7 | 115.2 | 121 | 118.6 | 98.8 | 96.8 | 102.4 | 7.00366E-05 |
| Q8BGT7 | Smndc1 | 107.8 | 111.2 | 111.2 | 118.3 | 106.2 | 109 | 76.7 | 72.5 | 77.5 | 101.8 | 105.4 | 102.4 | 8.86508E-05 |
| A2AJI0 | Map7d1 | 102 | 100.4 | 102.1 | 118.4 | 113.1 | 113.4 | 78.3 | 74.7 | 85.5 | 102.9 | 105.6 | 103.6 | 8.2169E-05 |
| P10922 | H1-0 | 86.4 | 92.2 | 91.8 | 81.7 | 83.3 | 74.5 | 145.5 | 127.8 | 143.3 | 92.4 | 86.4 | 94.8 | 9.92272E-05 |
| Q8BH95 | Echs1 | 90 | 94 | 94.3 | 88.7 | 87.1 | 93.7 | 121.5 | 118.3 | 116.5 | 100.1 | 95.6 | 100.2 | 7.43152E-05 |
| Q8K4R9 | Dlgap5 | 95.2 | 94.6 | 93.7 | 119.4 | 118.6 | 120.8 | 74.3 | 74.3 | 78.4 | 108.1 | 112.7 | 110 | 3.35696E-06 |
| Q8BH24 | Tm9sf4 | 88.9 | 83.2 | 87.1 | 87 | 93 | 89 | 125.1 | 131.9 | 121.5 | 97 | 98.6 | 97.6 | 4.65824E-05 |
| Q2TBE6 | Pi4k2a | 93.1 | 92.5 | 94.4 | 95 | 94.7 | 93.7 | 114.7 | 115.5 | 112.2 | 96.5 | 98.1 | 99.4 | 1.70253E-05 |

| Protein Accession | Gene Name | Control-1 | Control-2 | Control-3 | APAP 100-1 | APAP 100-2 | APAP 100-3 | APAP 500-1 | APAP 500-2 | APAP 500-3 | APAP-HU-1 | APAP-HU-2 | APAP-HU-3 | Adjust pvalue |
|---|---|---|---|---|---|---|---|---|---|---|---|---|---|---|
| O70503 | Hsd17b12 | 95.4 | 98.7 | 94.4 | 89.6 | 91 | 92.1 | 115.1 | 118.2 | 112.8 | 98.4 | 96.3 | 97.9 | 4.29071E-05 |
| P70227 | Itpr3 | 126.2 | 119.2 | 124.6 | 82.4 | 87.2 | 82.4 | 110.7 | 113 | 112.6 | 78 | 83.4 | 80.2 | 1.6831E-05 |
| O08915 | Aip | 111.7 | 113.7 | 110.5 | 97.3 | 96.3 | 96.8 | 96.8 | 96.4 | 95.5 | 92.9 | 96.2 | 96 | 2.79614E-05 |
| Q9EQ28 | Pold3 | 104.8 | 110.7 | 106 | 107.9 | 107.1 | 106.1 | 85.2 | 83.3 | 85.4 | 102.9 | 103 | 97.5 | 8.46186E-05 |
| P55096 | Abcd3 | 88.1 | 84.9 | 90.3 | 86.2 | 96.5 | 89.6 | 126.3 | 133.8 | 125.9 | 90.2 | 94.8 | 93.4 | 6.20583E-05 |
| Q05117 | Acp5 | 74.9 | 75.6 | 76.1 | 101.3 | 98.6 | 99.3 | 108 | 104.2 | 107.7 | 122.3 | 114.4 | 117.5 | 1.70253E-05 |
| O88783 | F5 | 44.3 | 44.5 | 42.9 | 73.1 | 74.9 | 68.3 | 185.6 | 201.8 | 174.1 | 104.8 | 88.2 | 97.7 | 1.70253E-05 |
| Q9JJ11 | Tacc3 | 76.7 | 79.4 | 78.8 | 131.4 | 113.9 | 128.8 | 76.1 | 77.7 | 81.4 | 121.6 | 117 | 117.2 | 6.46683E-05 |
| Q80W54 | Zmpste24 | 87.5 | 84.6 | 91.2 | 90.6 | 95.6 | 93.1 | 120 | 125.5 | 117.4 | 97.9 | 97.5 | 99.1 | 6.25225E-05 |
| P97494 | Gclc | 95.8 | 92.2 | 94.4 | 82.2 | 86.5 | 86 | 116.3 | 126 | 116.7 | 100.6 | 101.9 | 101.4 | 7.39442E-05 |
| Q80U58 | Pum2 | 111.3 | 112.8 | 107 | 115.2 | 110.1 | 107.1 | 79.3 | 75.2 | 82.5 | 99.7 | 101.6 | 98.2 | 8.2169E-05 |
| Q9JLI6 | Scly | 112.6 | 109.2 | 116 | 100.7 | 103.8 | 103.7 | 87.3 | 89.2 | 86.4 | 97 | 98 | 96.1 | 6.05346E-05 |
| Q922S8 | Kif2c | 94.5 | 90.4 | 93.6 | 120.2 | 116.7 | 117.6 | 81.8 | 84.7 | 84 | 103 | 109.8 | 103.8 | 2.82228E-05 |
| P52633 | Stat6 | 112.3 | 112.6 | 112.8 | 97.4 | 100.8 | 99.8 | 87.2 | 92.6 | 92.2 | 95.5 | 98.8 | 98 | 7.33727E-05 |
| Q6ZPY7 | Kdm3b | 113.9 | 115.3 | 117.9 | 102.8 | 102.2 | 103.4 | 84.5 | 88.9 | 87 | 92.3 | 96.1 | 95.7 | 2.24605E-05 |
| Q62433 | Ndrg1 | 117.3 | 112.9 | 117 | 100.7 | 102.6 | 99.8 | 85.9 | 88.4 | 91.9 | 91.1 | 97 | 95.5 | 8.76837E-05 |
| Q8K078 | Slco4a1 | 90 | 87.5 | 83.4 | 83.9 | 87.3 | 86.9 | 129.4 | 133.3 | 130.5 | 93.7 | 99.3 | 94.7 | 1.2797E-05 |
| Q9D1E6 | Tbcb | 111.2 | 113.2 | 117.2 | 101.4 | 99.8 | 102.4 | 84.2 | 86.1 | 82.1 | 102.2 | 101.2 | 99 | 3.49561E-05 |
| O88531 | Ppt1 | 82.7 | 88.9 | 91 | 94.5 | 92.5 | 94.6 | 120.2 | 122 | 116 | 99.6 | 97.6 | 100.4 | 6.42423E-05 |
| Q8JZN5 | Acad9 | 90.7 | 80.2 | 87.1 | 86.1 | 91.9 | 89.3 | 127.6 | 141.1 | 129.9 | 90.4 | 93.8 | 91.8 | 8.13629E-05 |
| Q8R1V4 | Tmed4 | 74.3 | 74.4 | 76.5 | 98.7 | 97 | 90.1 | 117.5 | 126.4 | 127.1 | 106.3 | 103.2 | 108.4 | 4.61936E-05 |

| Protein Accession | Gene Name | Control-1 | Control-2 | Control-3 | APAP 100-1 | APAP 100-2 | APAP 100-3 | APAP 500-1 | APAP 500-2 | APAP 500-3 | APAP-HU-1 | APAP-HU-2 | APAP-HU-3 | Adjust pvalue |
|---|---|---|---|---|---|---|---|---|---|---|---|---|---|---|
| Q9D6Y9 | Gbe1 | 103.1 | 102.3 | 104.4 | 89.9 | 94.1 | 91.7 | 109.1 | 112.9 | 110.2 | 92.2 | 95.7 | 94.3 | 6.37561E-05 |
| Q8C8U0 | Ppfibp1 | 120 | 117.1 | 122.4 | 105.3 | 106.4 | 109.4 | 78.5 | 79.5 | 80.3 | 90.6 | 97.6 | 92.8 | 2.00362E-05 |
| Q8BFZ9 | Erlin2 | 77.6 | 79.4 | 76.1 | 90.5 | 88.8 | 87.4 | 125.5 | 137.6 | 127.9 | 105.5 | 99.1 | 104.6 | 3.09919E-05 |
| Q9WUM5 | Suclg1 | 93.9 | 94.4 | 89 | 89.2 | 90.4 | 91.1 | 122.5 | 124.8 | 115.8 | 97.6 | 96.7 | 94.7 | 6.46683E-05 |
| O35609 | Scamp3 | 89 | 87.2 | 87.9 | 93.2 | 101.2 | 93.1 | 118 | 117.7 | 115.3 | 98.4 | 98.4 | 100.6 | 7.05721E-05 |
| Q99JR1 | Sfxn1 | 85.1 | 85.5 | 87.2 | 96.2 | 95.8 | 91.1 | 117.4 | 123 | 122.8 | 100.7 | 96.1 | 99.1 | 2.96388E-05 |
| Q61771 | Kif3b | 81.5 | 83.3 | 81 | 99.1 | 94.7 | 91.1 | 117 | 121.8 | 123.7 | 103.7 | 99.2 | 103.8 | 4.47401E-05 |
| Q9CQF9 | Pcyox1 | 75.4 | 77 | 78.6 | 96.1 | 93.6 | 90.1 | 125.6 | 131.1 | 127.5 | 101.8 | 97.7 | 105.6 | 1.76997E-05 |
| Q9Z0J0 | Npc2 | 78.8 | 78.8 | 77.7 | 102.7 | 100.1 | 101.2 | 112.2 | 113.3 | 111.3 | 113.8 | 105.1 | 105.1 | 3.93858E-05 |
| Q8BLH7 | Hirip3 | 132.4 | 126.9 | 128.9 | 111.3 | 111 | 110.7 | 65.8 | 65 | 68.7 | 91.4 | 96.8 | 91.2 | 2.35904E-06 |
| Q9D172 | Gatd3a | 89.2 | 90.6 | 92 | 89 | 89.7 | 89.4 | 125 | 130.7 | 119.6 | 94.9 | 94.8 | 95.1 | 3.25629E-05 |
| Q9D379 | Ephx1 | 75.6 | 74.9 | 77 | 98.9 | 95.2 | 90.8 | 121.8 | 129.7 | 125.1 | 103.5 | 101.5 | 105.9 | 2.24605E-05 |
| Q3TDN2 | Faf2 | 90.3 | 89 | 94.4 | 96.4 | 91.9 | 92.6 | 114 | 121.1 | 118.5 | 95.1 | 97.9 | 98.8 | 9.74953E-05 |
| Q9ESW4 | Agk | 87.5 | 85.3 | 89.2 | 86.9 | 93.9 | 90.3 | 125 | 136.1 | 125.9 | 91.2 | 94.9 | 93.9 | 5.27444E-05 |
| Q9D0S9 | Hint2 | 74.9 | 76.7 | 77.7 | 96 | 96.1 | 87.4 | 126.7 | 130.1 | 127.7 | 102.2 | 99.4 | 105.2 | 1.96502E-05 |
| O08663 | Metap2 | 120.2 | 122.3 | 118.6 | 100.9 | 100.1 | 102.9 | 79.4 | 84.5 | 78.2 | 96.2 | 97.5 | 99.3 | 1.72675E-05 |
| Q64FW2 | Retsat | 81 | 77.9 | 81.5 | 91.2 | 91.7 | 87.2 | 128 | 138.2 | 136.9 | 90.9 | 98.5 | 96.9 | 2.61375E-05 |
| P52825 | Cpt2 | 94.9 | 90.9 | 91.9 | 92.1 | 91 | 94 | 118.7 | 118.9 | 114.6 | 95.4 | 97.9 | 99.6 | 3.5691E-05 |
| Q9DBS5 | Klc4 | 88.2 | 88.3 | 91.3 | 88.3 | 92.2 | 94 | 119.6 | 119.1 | 115.3 | 98.9 | 103.1 | 101.7 | 4.05129E-05 |
| Q8R344 | Ccdc12 | 104.2 | 113.1 | 107.6 | 121.6 | 109.6 | 118.1 | 71.2 | 67 | 75.2 | 104.7 | 104.2 | 103.4 | 8.24174E-05 |
| Q8BLF1 | Nceh1 | 80.5 | 82 | 82.1 | 90.1 | 90.2 | 85.6 | 129.8 | 138.6 | 127.6 | 99.2 | 95.2 | 99.1 | 2.38593E-05 |
| P56391 | Cox6b1 | 76.3 | 74.2 | 66 | 102.4 | 96.6 | 97.6 | 124 | 115.9 | 119.6 | 112.4 | 104.3 | 110.6 | 7.15723E-05 |
| Q08024 | Cbfb | 105.4 | 108.4 | 108.5 | 109.7 | 107.5 | 104.9 | 81.3 | 86.7 | 82.8 | 103.2 | 102.1 | 99.6 | 5.8088E-05 |

| Protein Accession | Gene Name | Control-1 | Control-2 | Control-3 | APAP 100-1 | APAP 100-2 | APAP 100-3 | APAP 500-1 | APAP 500-2 | APAP 500-3 | APAP-HU-1 | APAP-HU-2 | APAP-HU-3 | Adjust pvalue |
|---|---|---|---|---|---|---|---|---|---|---|---|---|---|---|
| A3KGF7 | Plcb2 | 110.6 | 112.6 | 111.2 | 105.9 | 104.8 | 105.2 | 82.9 | 82 | 86.6 | 96 | 102.5 | 99.6 | 4.05129E-05 |
| Q3V300 | Kif22 | 84.2 | 86.8 | 85.1 | 108.5 | 106.6 | 106.2 | 93.9 | 102.3 | 100 | 108.9 | 109.5 | 108 | 8.86508E-05 |
| P10923 | Spp1 | 74 | 75.5 | 72.6 | 135 | 126.9 | 137.7 | 61.3 | 63.7 | 72.3 | 127.5 | 124 | 129.5 | 1.2797E-05 |
| Q7TMF3 | Ndufa12 | 81.7 | 87.9 | 88 | 98.2 | 98 | 95.8 | 113.3 | 114 | 112.4 | 106 | 100.8 | 103.9 | 6.65487E-05 |
| P53395 | Dbt | 84.7 | 83.4 | 84.4 | 92 | 85.8 | 89.2 | 125.1 | 130.6 | 126.3 | 101.4 | 95.9 | 101.2 | 1.76997E-05 |
| Q9D0D4 | Dimt1 | 109.7 | 112.7 | 111.7 | 99.5 | 97.3 | 98.8 | 92.2 | 92.8 | 90.7 | 97.9 | 100.4 | 96.1 | 4.63182E-05 |
| Q9CYR0 | Ssbp1 | 80.7 | 84.4 | 81.3 | 95 | 98 | 95.4 | 121.4 | 113.5 | 121.2 | 105.2 | 98.7 | 105.4 | 7.31812E-05 |
| Q8CDM1 | Atad2 | 80.9 | 75.9 | 80 | 79.5 | 86.8 | 86.4 | 139.9 | 151.1 | 143.2 | 89.9 | 94.1 | 92.4 | 1.63466E-05 |
| Q9CXE7 | Tmed5 | 80.4 | 83.8 | 80.4 | 101.3 | 98.2 | 96.7 | 112.6 | 112 | 114.8 | 109.4 | 103.9 | 106.8 | 2.70786E-05 |
| Q6NXI6 | Rprd2 | 109.2 | 108.7 | 107.3 | 109.5 | 104.8 | 107 | 84.9 | 84.8 | 88.8 | 96.9 | 99.8 | 98.3 | 4.19587E-05 |
| Q80Y14 | Glrx5 | 88 | 90.3 | 89.5 | 91.7 | 93.1 | 92.5 | 125.3 | 118.4 | 118.4 | 99.9 | 97.4 | 95.7 | 2.95731E-05 |
| P09581 | Csf1r | 124.6 | 132.7 | 135.3 | 95 | 100.9 | 99.3 | 84.6 | 87.7 | 87.9 | 82.9 | 84.2 | 84.7 | 2.53608E-05 |
| P30204 | Msr1 | 107.7 | 113.4 | 109.6 | 110.9 | 106.3 | 104.2 | 75.5 | 77.6 | 76.5 | 107.5 | 104.8 | 105.9 | 2.38593E-05 |
| Q9Z0R9 | Fads2 | 134.5 | 136.3 | 145.6 | 118.7 | 117.6 | 117.8 | 68.7 | 72.9 | 72.2 | 71.5 | 70.6 | 73.5 | 4.40366E-06 |
| Q9Z0H4 | Celf2 | 112.4 | 112 | 109.7 | 100.5 | 97.8 | 104 | 87.4 | 88.2 | 87 | 100 | 100.3 | 100.7 | 4.08553E-05 |
| P04441 | Cd74 | 63.9 | 70.5 | 67.6 | 97.6 | 89 | 84.3 | 147 | 138.3 | 144.1 | 101.5 | 95.2 | 100.9 | 2.22534E-05 |
| Q3TVI8 | Pbxip1 | 66 | 69.3 | 68.9 | 98.4 | 92.2 | 99.7 | 140.7 | 124 | 132.9 | 102.7 | 99.6 | 105.5 | 4.61936E-05 |
| Q61425 | Hadh | 83.6 | 84.2 | 86.3 | 89.4 | 91 | 88.4 | 128.3 | 134.4 | 131.7 | 93.6 | 93.6 | 95.6 | 2.59433E-06 |
| Q9CR62 | Slc25a11 | 80.5 | 86.2 | 86.5 | 94.2 | 93.4 | 87.8 | 126.3 | 123 | 125.4 | 99.7 | 94 | 102.9 | 5.15591E-05 |
| Q9CPV5 | Pmf1 | 99.6 | 106.1 | 106 | 113.5 | 109.6 | 113.4 | 78.8 | 80.6 | 85 | 102.3 | 103.6 | 101.4 | 7.15723E-05 |
| O88384 | Vti1b | 78.3 | 79.3 | 81.5 | 104.9 | 99.6 | 97.1 | 110.1 | 112.8 | 117.3 | 103.6 | 105.5 | 110 | 9.40516E-05 |
| Q8R4N0 | Clybl | 87 | 89.3 | 91.6 | 90.5 | 88.9 | 87.6 | 118.5 | 131.2 | 125.9 | 95.2 | 96.2 | 98.2 | 6.85537E-05 |
| Q9D666 | Sun1 | 82 | 77.6 | 83.8 | 92.6 | 94.9 | 88.5 | 129.9 | 136.1 | 126.5 | 93.8 | 97.5 | 96.8 | 2.76658E-05 |

| Protein Accession | Gene Name | Control-1 | Control-2 | Control-3 | APAP 100-1 | APAP 100-2 | APAP 100-3 | APAP 500-1 | APAP 500-2 | APAP 500-3 | APAP-HU-1 | APAP-HU-2 | APAP-HU-3 | Adjust pvalue |
|---|---|---|---|---|---|---|---|---|---|---|---|---|---|---|
| Q9QXE7 | Tbl1x | 114.4 | 117.9 | 116.7 | 99.4 | 94.4 | 98.6 | 93.3 | 92.3 | 88.4 | 92.8 | 97.8 | 94.1 | 8.26329E-05 |
| Q8R059 | Gale | 87.8 | 88.7 | 88.7 | 88.8 | 88.7 | 86.6 | 124.2 | 133.1 | 128.8 | 94.9 | 95.4 | 94.3 | 1.18749E-05 |
| Q9CQC7 | Ndufb4 | 65.6 | 65.3 | 67.4 | 102.1 | 100.2 | 90.2 | 126.2 | 129.9 | 127.5 | 112.4 | 101.4 | 111.9 | 4.01713E-05 |
| P03975 | Iap | 101.4 | 94.1 | 96.8 | 82.7 | 92.8 | 87 | 124.7 | 124.3 | 123.2 | 89 | 92.6 | 91.4 | 5.8088E-05 |
| Q9CQY5 | Magt1 | 89.8 | 90.6 | 94.5 | 98.7 | 96.7 | 94.7 | 111.6 | 112.2 | 115 | 98.1 | 97.4 | 100.8 | 9.43575E-05 |
| Q8CFE6 | Slc38a2 | 110.1 | 116.1 | 119.8 | 108.5 | 106.1 | 106.5 | 75.7 | 81.4 | 77.4 | 99.6 | 98 | 100.8 | 4.67397E-05 |
| Q9JHW4 | Eefsec | 95 | 94.3 | 95.7 | 94.9 | 90.1 | 89.3 | 116.8 | 121.6 | 122 | 90.6 | 94.3 | 95.2 | 4.19587E-05 |
| Q9ERR7 | Selenof | 72.9 | 80.2 | 73.9 | 96.5 | 88.6 | 91.2 | 124.9 | 125.7 | 131.2 | 106.3 | 99.5 | 109.1 | 4.78E-05 |
| Q99KB8 | Hagh | 87 | 89 | 85.2 | 95.3 | 89.2 | 92.9 | 121 | 120.3 | 117.5 | 103 | 98.8 | 100.7 | 2.75949E-05 |
| P47740 | Aldh3a2 | 92.7 | 93.7 | 97.9 | 95.5 | 91.1 | 93.7 | 115.1 | 117.5 | 114.3 | 96.4 | 94.1 | 98 | 7.00366E-05 |
| Q9CZA6 | Nde1 | 89.4 | 87.8 | 89.7 | 118.9 | 114.5 | 113.7 | 82.3 | 84.7 | 92.4 | 108.1 | 108.9 | 109.8 | 7.24382E-05 |
| Q8QZS1 | Hibch | 89.7 | 85 | 88.1 | 80.8 | 83 | 84.3 | 135.2 | 146 | 138.3 | 87.8 | 91.2 | 90.6 | 1.26945E-05 |
| Q9QZS3 | Numb | 120.9 | 122.3 | 121.1 | 110.2 | 102.7 | 109 | 74.1 | 72.7 | 76.2 | 94.5 | 100.1 | 96.3 | 1.70253E-05 |
| Q9CX56 | Psmd8 | 97.9 | 99.4 | 96.8 | 91.9 | 95 | 92.5 | 111.2 | 112.8 | 110.9 | 94.4 | 99.1 | 98.1 | 7.31812E-05 |
| Q9EPK6 | Sil1 | 90.9 | 102.3 | 95.4 | 85.9 | 85.6 | 83.2 | 126.6 | 129 | 123.9 | 92.1 | 92.7 | 92.4 | 3.94161E-05 |
| Q9R0Q4 | Morf4l2 | 99.9 | 106.7 | 102.9 | 127.2 | 121.5 | 120.2 | 60.7 | 59.2 | 63.1 | 115.3 | 113.2 | 110.1 | 7.81516E-06 |
| P22315 | Fech | 92.1 | 88.9 | 95 | 90.5 | 90.3 | 88.1 | 121.9 | 134.9 | 124.2 | 90.2 | 93.8 | 90.1 | 8.7241E-05 |
| Q8K0C4 | Cyp51a1 | 127 | 121.4 | 124.4 | 95.4 | 99.9 | 101.8 | 82.6 | 91.2 | 85.6 | 87.9 | 94.6 | 88.2 | 7.33727E-05 |
| Q9D1C1 | Ube2c | 100 | 104.9 | 101 | 132.2 | 120.7 | 125.2 | 62.8 | 68.3 | 69.4 | 104.9 | 105 | 105.7 | 2.23166E-05 |
| Q3ULF4 | Spg7 | 85.8 | 85.5 | 89.9 | 96.2 | 94.9 | 93.7 | 116.7 | 125.7 | 124.7 | 93 | 97.3 | 96.7 | 5.80601E-05 |
| Q8VEH8 | Erlec1 | 84.4 | 81.2 | 84.7 | 96.6 | 91.6 | 87.6 | 120.1 | 129.5 | 125.1 | 103.4 | 97 | 98.8 | 7.30482E-05 |
| Q9Z1S0 | Bub1b | 104.7 | 104.2 | 110.8 | 117.4 | 118.8 | 119.4 | 70.2 | 72.1 | 76.1 | 99.5 | 107.2 | 99.6 | 3.22317E-05 |
| Q9CQE1 | Nipsnap | 73.7 | 76.1 | 80.9 | 97.1 | 93.7 | 89.3 | 131.4 | 128.6 | 125.9 | 103.6 | 97.4 | 102.3 | 2.61375E-05 |

| Protein Accession | Gene Name | Control-1 | Control-2 | Control-3 | APAP 100-1 | APAP 100-2 | APAP 100-3 | APAP 500-1 | APAP 500-2 | APAP 500-3 | APAP-HU-1 | APAP-HU-2 | APAP-HU-3 | Adjust pvalue |
|---|---|---|---|---|---|---|---|---|---|---|---|---|---|---|
| | 3b | | | | | | | | | | | | | |
| Q8JZK9 | Hmgcs1 | 146.2 | 131.4 | 136.9 | 100.3 | 112 | 114.1 | 60.1 | 67.4 | 65.3 | 85.4 | 93.6 | 87.3 | 4.92243E-05 |
| P30412 | Ppic | 72.6 | 74.8 | 76.3 | 108.1 | 102.2 | 102.9 | 112.8 | 115.5 | 115.3 | 106.4 | 102.9 | 110.3 | 2.53795E-05 |
| P09450 | Junb | 99.9 | 99.1 | 100 | 114 | 110.8 | 108.1 | 83.4 | 80.7 | 86.1 | 104.1 | 105.9 | 107.8 | 4.47401E-05 |
| Q9JHI5 | Ivd | 87.1 | 86.4 | 88.3 | 89.5 | 93.9 | 93.8 | 118.3 | 126.4 | 122.9 | 96.6 | 99.8 | 97.1 | 2.95731E-05 |
| Q91VC9 | Ghitm | 77.8 | 77.3 | 81.4 | 100.2 | 99.4 | 92.7 | 119 | 126.4 | 125.7 | 100.2 | 96.2 | 103.7 | 5.61462E-05 |
| P21126 | Ubl4a | 95.4 | 97.9 | 97.7 | 109.6 | 106.2 | 105.6 | 87.7 | 88.7 | 89.7 | 107 | 105.7 | 108.8 | 4.00294E-05 |
| Q8K009 | Aldh1l2 | 99.6 | 88.1 | 93 | 75 | 86.3 | 81.4 | 136.9 | 143.6 | 132.7 | 84.3 | 89.2 | 89.8 | 5.87966E-05 |
| Q64430 | Atp7a | 90.9 | 88.9 | 95 | 92.8 | 95.4 | 92.8 | 117.8 | 122.9 | 117.5 | 92.8 | 95.4 | 97.6 | 5.85888E-05 |
| E9Q1P8 | Irf2bp2 | 118.1 | 123.8 | 117.3 | 113.5 | 106.3 | 107.9 | 73.4 | 75.2 | 80.1 | 89.5 | 100.5 | 94.4 | 8.64579E-05 |
| Q8BWW4 | Larp4 | 107.7 | 106 | 107.7 | 109.7 | 108.8 | 110.9 | 81.2 | 79.5 | 84.1 | 101 | 100.8 | 102.7 | 9.56192E-06 |
| Q9DB73 | Cyb5r1 | 81 | 80.5 | 76.4 | 96.9 | 96.9 | 91.6 | 121.7 | 129 | 122.2 | 100.3 | 97.9 | 105.5 | 4.47401E-05 |
| P48725 | Pcnt | 96 | 99 | 93.9 | 89.6 | 93.7 | 93.2 | 114.2 | 121.7 | 120.8 | 90.1 | 94.5 | 93.2 | 9.8067E-05 |
| Q99JF5 | Mvd | 120.6 | 116.3 | 123.9 | 95.3 | 98.1 | 98.7 | 88.1 | 91.7 | 86 | 90.9 | 97 | 93.5 | 7.39442E-05 |
| Q9QWF0 | Chaf1a | 122.2 | 112.6 | 118.3 | 107.4 | 111.3 | 112.9 | 73.1 | 80 | 80.2 | 92.8 | 97.2 | 91.9 | 7.07414E-05 |
| Q9CQL7 | Mrfap1 | 90.2 | 92.5 | 91.3 | 132.5 | 123.1 | 122.3 | 71 | 69.7 | 74.3 | 111.3 | 110.1 | 111.7 | 1.70253E-05 |
| Q9D3P8 | Plgrkt | 67.9 | 72.5 | 71.6 | 105.6 | 101 | 95.9 | 120.8 | 115.6 | 120.3 | 111.2 | 102.9 | 114.7 | 6.7414E-05 |
| Q9Z2G9 | Htatip2 | 88.6 | 89.2 | 95 | 93.8 | 94.6 | 91.8 | 122 | 119.4 | 117.6 | 96.9 | 93.4 | 97.7 | 4.61936E-05 |
| P07607 | Tyms | 95.6 | 95.5 | 100.6 | 111.6 | 110.5 | 108 | 85.1 | 88.1 | 87.7 | 105.7 | 105.6 | 106.1 | 4.63182E-05 |
| Q00422 | Gabpa | 111.7 | 113.6 | 117.5 | 99.4 | 98.4 | 98.9 | 94.6 | 94.6 | 92 | 93.2 | 94.3 | 91.8 | 4.19587E-05 |
| Q99M54 | Cdca3 | 84 | 85.2 | 91.7 | 124.3 | 116.8 | 125.2 | 81.8 | 74.3 | 79.3 | 114.6 | 108.8 | 114 | 5.03983E-05 |
| P97477 | Aurka | 79.1 | 80.9 | 83.4 | 118 | 114 | 115.5 | 91.6 | 93.1 | 90.6 | 110.2 | 113.9 | 109.7 | 1.18749E-05 |
| Q9CXX9 | Cuedc2 | 82.9 | 87.9 | 88.9 | 136.5 | 118.9 | 130.2 | 66.6 | 68 | 73.7 | 119.5 | 112.1 | 114.9 | 6.20583E-05 |

| Protein Accession | Gene Name | Control-1 | Control-2 | Control-3 | APAP 100-1 | APAP 100-2 | APAP 100-3 | APAP 500-1 | APAP 500-2 | APAP 500-3 | APAP-HU-1 | APAP-HU-2 | APAP-HU-3 | Adjust pvalue |
|---|---|---|---|---|---|---|---|---|---|---|---|---|---|---|
| Q8R164 | Bphl | 85.4 | 87.4 | 88.3 | 91.5 | 89 | 88.4 | 126.8 | 128.2 | 124.9 | 94 | 96.5 | 99.6 | 7.18133E-06 |
| Q9CQV1 | Pam16 | 70.8 | 74.5 | 71.7 | 105.8 | 100.9 | 94.6 | 123.8 | 118.9 | 126 | 106.8 | 97.4 | 108.9 | 8.79721E-05 |
| Q91XU3 | Pip4k2c | 81.2 | 81.4 | 84.2 | 92.9 | 95.9 | 94.5 | 126.3 | 123.2 | 123.7 | 97.6 | 97.8 | 101.4 | 4.96362E-06 |
| O08603 | Raet1b | 73.5 | 80.5 | 79.5 | 121.4 | 111.8 | 113 | 95.9 | 94.4 | 97.9 | 114.8 | 107.5 | 109.7 | 9.74953E-05 |
| Q9Z1P6 | Ndufa7 | 83.1 | 80.4 | 83.8 | 102.1 | 99.6 | 97.4 | 111.9 | 115.4 | 115.9 | 102.1 | 100.3 | 108 | 6.61856E-05 |
| Q99L13 | Hibadh | 87.9 | 90.1 | 93.5 | 90.8 | 90.1 | 89.6 | 123.1 | 130.4 | 124.4 | 92.6 | 93.5 | 94 | 1.91235E-05 |
| Q9CQR4 | Acot13 | 74 | 75.3 | 78.5 | 90.2 | 88.8 | 86.1 | 137.1 | 139 | 129.3 | 102.6 | 96.4 | 102.7 | 1.76997E-05 |
| Q9D7P6 | Iscu | 91.5 | 90.4 | 88.8 | 100.8 | 97.1 | 96.2 | 110.6 | 110.9 | 111.1 | 101.3 | 101.2 | 100.2 | 3.11077E-05 |
| Q3TFK5 | Gpatch4 | 107.2 | 109.5 | 111.5 | 112.3 | 109.8 | 104.8 | 82.1 | 78 | 84.4 | 100 | 99.8 | 100.7 | 7.14796E-05 |
| P52482 | Ube2e1 | 102 | 103.5 | 100.4 | 105.9 | 107.2 | 104.8 | 89.4 | 88.6 | 90.4 | 102.5 | 102.8 | 102.6 | 2.24605E-05 |
| P49025 | Cit | 91.8 | 85.1 | 87.5 | 119.1 | 118.4 | 120.1 | 84 | 88.9 | 92.3 | 103.2 | 107.5 | 102 | 6.91539E-05 |
| Q8CHY6 | Gatad2a | 102.9 | 107.2 | 105.9 | 116.3 | 108.9 | 109.2 | 82 | 77.9 | 82.2 | 99.6 | 105.4 | 102.6 | 9.63387E-05 |
| Q921J4 | Ube2s | 72.3 | 76.3 | 74.9 | 123.3 | 117 | 116.1 | 85 | 86.9 | 90.7 | 119.5 | 118.5 | 119.4 | 1.09895E-05 |
| Q8K021 | Scamp1 | 86 | 83.4 | 88.9 | 98.1 | 98.1 | 94.7 | 114.1 | 119 | 118.5 | 98.8 | 98.8 | 101.4 | 4.01713E-05 |
| P24288 | Bcat1 | 113.9 | 115.5 | 118.6 | 104.7 | 109.1 | 108.1 | 77.8 | 81.5 | 81.8 | 91.1 | 101.9 | 95.9 | 8.3239E-05 |
| Q9CWG8 | Ndufaf7 | 85.6 | 88.2 | 90.9 | 92.1 | 91.6 | 92.7 | 119.6 | 131.8 | 123.8 | 94.2 | 94.5 | 94.9 | 5.97094E-05 |
| O70126 | Aurkb | 75.7 | 78.5 | 78.1 | 119.9 | 114.6 | 113.3 | 89 | 101.2 | 94.3 | 114.4 | 110.7 | 110.4 | 7.53408E-05 |
| Q80ZV0 | Rnaseh2b | 107.1 | 112.6 | 113.4 | 106.5 | 105.8 | 108.6 | 81.2 | 86.2 | 86.6 | 96 | 98.8 | 97.3 | 7.39442E-05 |
| Q80YP0 | Cdk3 | 47.5 | 44.6 | 43.7 | 69 | 89.5 | 65.7 | 168.5 | 176.7 | 192.2 | 95 | 98.3 | 109.4 | 3.09919E-05 |
| Q99KR7 | Ppif | 81.5 | 77.6 | 85.2 | 94.5 | 93.7 | 88.8 | 126.6 | 130.4 | 128.9 | 98 | 95.6 | 99.2 | 1.76997E-05 |
| Q8C166 | Cpne1 | 96 | 92.2 | 98.9 | 82.6 | 88.2 | 85.3 | 127.1 | 132.4 | 122.2 | 91.1 | 91 | 92.8 | 4.21484E-05 |
| Q91XD7 | Creld1 | 85.3 | 84.9 | 90.2 | 91.9 | 97.8 | 91 | 118 | 125.3 | 121.8 | 100.2 | 94.1 | 99.5 | 9.52528E-05 |

| Protein Accession | Gene Name | Control-1 | Control-2 | Control-3 | APAP 100-1 | APAP 100-2 | APAP 100-3 | APAP 500-1 | APAP 500-2 | APAP 500-3 | APAP-HU-1 | APAP-HU-2 | APAP-HU-3 | Adjust pvalue |
|---|---|---|---|---|---|---|---|---|---|---|---|---|---|---|
| Q8CHT0 | Aldh4a1 | 81.1 | 77 | 79.7 | 87 | 86.7 | 86.4 | 128.5 | 146.4 | 141.4 | 93 | 95.4 | 97.2 | 4.21059E-05 |
| Q99LZ3 | Gins4 | 106.2 | 109.7 | 111.6 | 108.4 | 108.3 | 106.4 | 80.2 | 85 | 87.6 | 100 | 98.5 | 98.1 | 7.46716E-05 |
| Q9CQW9 | Ifitm3 | 88 | 87 | 96.1 | 123.1 | 131.1 | 114.7 | 70.4 | 73.1 | 72.7 | 112 | 113 | 118.9 | 9.2914E-05 |
| Q8VE62 | Paip1 | 112.1 | 114.9 | 116.9 | 101 | 96.8 | 102.3 | 89.2 | 88.5 | 87 | 99.3 | 97.7 | 94.4 | 7.32331E-05 |
| Q9D7B6 | Acad8 | 93.6 | 93.5 | 91.2 | 87.9 | 90.6 | 89 | 120.3 | 125.5 | 117.5 | 93 | 100.1 | 97.8 | 5.97094E-05 |
| Q05920 | Pc | 82.2 | 82.9 | 87.4 | 86.6 | 93.9 | 87.9 | 127.2 | 138 | 140.6 | 88.3 | 91.1 | 94 | 4.8877E-05 |
| O35604 | Npc1 | 92.8 | 89 | 95.4 | 87 | 94.2 | 89.2 | 118.7 | 125.1 | 126.2 | 94.6 | 94.7 | 93.2 | 6.54149E-05 |
| Q91ZE0 | Tmlhe | 96.2 | 87.4 | 96.8 | 84.3 | 90 | 88.6 | 124.1 | 131.6 | 123.7 | 88.9 | 94.3 | 94.2 | 9.95431E-05 |
| Q64442 | Sord | 103.1 | 99.2 | 102.5 | 111.4 | 110.6 | 113.7 | 84.9 | 84.8 | 87.1 | 100.2 | 104.5 | 98 | 6.46683E-05 |
| Q9DCZ4 | Apoo | 81.9 | 85.5 | 84 | 93.4 | 96.7 | 89.4 | 119.7 | 123.4 | 120.7 | 100.9 | 99.4 | 104.9 | 3.04874E-05 |
| Q9CXR1 | Dhrs7 | 92 | 83.6 | 94.7 | 86.8 | 90.7 | 88.1 | 129.9 | 139.5 | 125.9 | 87.1 | 90.4 | 91.3 | 9.52528E-05 |
| Q8JZU2 | Slc25a1 | 98.3 | 92.6 | 91.9 | 85.5 | 88.3 | 88.7 | 123.2 | 131.2 | 121.8 | 91.6 | 92.4 | 94.4 | 4.90078E-05 |
| Q61823 | Pdcd4 | 87.7 | 90 | 89.2 | 83.5 | 77.9 | 84 | 138 | 139.7 | 136.8 | 91 | 91.8 | 90.5 | 1.9039E-06 |
| O35405 | Pld3 | 66.6 | 68.2 | 71.6 | 91.1 | 93.4 | 90 | 131.7 | 136.4 | 132.8 | 105.8 | 104.5 | 107.9 | 1.9039E-06 |
| Q03267 | Ikzf1 | 119.1 | 115.8 | 124.5 | 103.5 | 106.3 | 108.8 | 74.5 | 75.2 | 82.8 | 95.6 | 98.5 | 95.3 | 5.97094E-05 |
| Q62086 | Pon2 | 86.1 | 91.4 | 91.5 | 94.1 | 92.5 | 90.3 | 115.9 | 124.1 | 121.7 | 96.2 | 96.5 | 99.6 | 7.00366E-05 |
| Q9QYR9 | Acot2 | 91.1 | 92.4 | 93.6 | 97.9 | 95.1 | 92.6 | 111.7 | 115.6 | 114.1 | 97 | 98 | 100.8 | 7.31812E-05 |
| Q3UVK0 | Ermp1 | 85.4 | 83.9 | 90.1 | 88.9 | 97.2 | 90.2 | 124.2 | 134.2 | 127.3 | 90.7 | 92.8 | 95 | 6.35329E-05 |
| Q8R080 | Gtse1 | 67.1 | 66.7 | 67.1 | 118.1 | 109.9 | 116.2 | 92 | 95 | 94 | 116.9 | 134.7 | 122.4 | 5.91773E-05 |
| Q9DBL1 | Acadsb | 84.9 | 85.5 | 85.7 | 89.3 | 85.7 | 87.6 | 122.4 | 134.3 | 124.5 | 100.6 | 99.4 | 99.9 | 3.52341E-05 |
| Q8K4F5 | Abhd11 | 83.9 | 86.9 | 87.1 | 96 | 93.3 | 89.1 | 122.2 | 120.3 | 119.8 | 100.6 | 98.3 | 102.7 | 2.4964E-05 |
| Q8BR63 | Fam177a1 | 78.3 | 83.3 | 82.4 | 110.9 | 109.3 | 106.3 | 100.7 | 97.7 | 103.3 | 110.5 | 107.6 | 109.9 | 4.21584E-05 |

| Protein Accession | Gene Name | Control-1 | Control-2 | Control-3 | APAP 100-1 | APAP 100-2 | APAP 100-3 | APAP 500-1 | APAP 500-2 | APAP 500-3 | APAP-HU-1 | APAP-HU-2 | APAP-HU-3 | Adjust pvalue |
|---|---|---|---|---|---|---|---|---|---|---|---|---|---|---|
| P24860 | Ccnb1 | 83.9 | 85.6 | 86.4 | 122.5 | 117.8 | 130.7 | 74.7 | 73.8 | 77.9 | 116.7 | 112.8 | 117.1 | 2.61897E-05 |
| Q9R1Z7 | Pts | 77.9 | 83.7 | 80.3 | 105.2 | 101.8 | 99.6 | 107 | 105.5 | 112.7 | 110.9 | 106.4 | 109.1 | 9.65963E-05 |
| Q05512 | Mark2 | 88.3 | 91.5 | 92 | 100.9 | 102.6 | 99.7 | 106.3 | 107.4 | 106.5 | 100.2 | 102.2 | 102.4 | 7.00366E-05 |
| Q61846 | Melk | 73.9 | 71.3 | 76.5 | 98 | 102.6 | 101.7 | 116.7 | 129.4 | 117.7 | 101.8 | 106.8 | 103.6 | 7.33727E-05 |
| Q8CFE2 | Hpf1 | 129 | 122 | 132.3 | 88.1 | 88.4 | 88 | 98.1 | 102.4 | 96.2 | 81.8 | 89.9 | 83.9 | 4.92243E-05 |
| Q8VBZ0 | Dhrsx | 86.5 | 89.6 | 86.6 | 94.3 | 95.5 | 93.2 | 117.1 | 113.2 | 120.1 | 104.6 | 98.7 | 100.7 | 6.1428E-05 |
| Q60780 | Gas7 | 122.4 | 117.4 | 125.6 | 94.6 | 100.5 | 100.3 | 81.2 | 84.8 | 83.5 | 95.1 | 98.2 | 96.3 | 4.19898E-05 |
| Q8VEB4 | Pla2g15 | 80.6 | 81.4 | 79.8 | 100.6 | 96.1 | 94.9 | 114.7 | 122.1 | 120.5 | 101.8 | 104.9 | 102.4 | 2.95731E-05 |
| Q9ERG0 | Lima1 | 85.2 | 83.7 | 87.2 | 104.8 | 100.8 | 106 | 100.5 | 97.5 | 101.4 | 112.4 | 108.3 | 112.2 | 6.88011E-05 |
| Q9JJ94 | Ssna1 | 114.7 | 123.3 | 122.1 | 118.6 | 112.3 | 116.5 | 62.1 | 62.8 | 64.8 | 102.4 | 102.2 | 98.2 | 1.21966E-05 |
| Q9CQX4 | Pclaf | 104.4 | 106.4 | 108.3 | 130 | 124.4 | 126.3 | 60.7 | 57.7 | 62.7 | 106.1 | 107.4 | 105.6 | 1.9039E-06 |
| Q60929 | Mef2a | 117.4 | 114.6 | 116.2 | 109.5 | 106.3 | 112.7 | 77.6 | 74.3 | 78.4 | 96.4 | 97.6 | 99.1 | 1.3882E-05 |
| Q9D6M3 | Slc25a22 | 86.5 | 85.7 | 84.1 | 96.3 | 88.7 | 94.8 | 121.8 | 127.7 | 119.7 | 101.8 | 97 | 95.9 | 6.65487E-05 |
| Q91XE8 | Tmem205 | 83.8 | 82.2 | 84 | 88.4 | 92.1 | 87.2 | 127.2 | 138.9 | 135.5 | 90.6 | 93.8 | 96.2 | 2.55575E-05 |
| Q921Q3 | Alg1 | 88.2 | 84.8 | 83.4 | 81.5 | 84.9 | 84.9 | 138.8 | 143.6 | 128.1 | 93.9 | 94.1 | 93.7 | 3.42332E-05 |
| Q8BVK9 | Sp110 | 120.8 | 120.7 | 126.8 | 106.5 | 92.9 | 104.4 | 77.5 | 71.6 | 74 | 99.5 | 103.9 | 101.4 | 9.42022E-05 |
| Q8BHG9 | Cggbp1 | 115 | 115.9 | 121.1 | 111.8 | 111.3 | 111 | 74.9 | 74.7 | 77.9 | 91.5 | 99 | 95.9 | 1.96502E-05 |
| Q9JKF6 | Nectin1 | 112.7 | 121.2 | 114 | 116.6 | 110.9 | 107.9 | 74 | 71.2 | 76.8 | 100.6 | 97.4 | 96.7 | 4.92243E-05 |
| P61025 | Cks1b | 102.8 | 96.4 | 90.3 | 140.7 | 127.7 | 134.8 | 54.4 | 50.6 | 55 | 122.5 | 115.2 | 109.6 | 2.88539E-05 |
| Q8BYI6 | Lpcat2 | 97.2 | 95.5 | 95.8 | 91.1 | 92 | 95.3 | 109.6 | 111.1 | 112.3 | 101.1 | 99.1 | 100 | 4.47401E-05 |
| Q9CYK1 | Wars2 | 78.5 | 80.8 | 80.5 | 91.8 | 87.2 | 82.5 | 128.9 | 139.9 | 133.9 | 99.7 | 97.6 | 98.7 | 2.38593E-05 |
| P28741 | Kif3a | 88.8 | 85.2 | 90.1 | 91.1 | 95 | 92.1 | 117 | 124.4 | 123 | 95 | 99.3 | 99.2 | 4.8877E-05 |

| Protein Accession | Gene Name | Control-1 | Control-2 | Control-3 | APAP 100-1 | APAP 100-2 | APAP 100-3 | APAP 500-1 | APAP 500-2 | APAP 500-3 | APAP-HU-1 | APAP-HU-2 | APAP-HU-3 | Adjust pvalue |
|---|---|---|---|---|---|---|---|---|---|---|---|---|---|---|
| P31651 | Slc6a12 | 102.3 | 96.8 | 106.4 | 83.5 | 89.4 | 87.4 | 127.4 | 130.3 | 122.5 | 83.5 | 83.8 | 86.7 | 4.39456E-05 |
| Q9CQE7 | Ergic3 | 80.9 | 90.6 | 85.5 | 96.4 | 93.2 | 89.8 | 122.7 | 124.1 | 120.9 | 98.6 | 95.1 | 102.2 | 8.64579E-05 |
| Q8BZR9 | Ncbp3 | 83.3 | 78.4 | 82.5 | 90.5 | 96.4 | 90.4 | 118.9 | 128.1 | 125.4 | 100.3 | 99.5 | 106.1 | 6.25225E-05 |
| Q8BXN9 | Tmem87a | 80.2 | 84.2 | 86 | 99.8 | 97.3 | 92.7 | 111.2 | 116.7 | 115.1 | 106.4 | 103.2 | 107.3 | 8.86508E-05 |
| Q8BUE4 | Aifm2 | 89.6 | 90.2 | 94.5 | 91.6 | 91.5 | 92.5 | 116.9 | 121.8 | 117.5 | 96.8 | 96.8 | 100.4 | 3.23568E-05 |
| Q64281 | Lilrb4 | 76.4 | 79.6 | 76.6 | 118.4 | 105.7 | 104.7 | 86.9 | 85.8 | 87 | 128.7 | 121.2 | 128.8 | 6.4123E-05 |
| P19221 | F2 | 35.4 | 35.7 | 31.7 | 61.9 | 62.6 | 56.3 | 178.7 | 231 | 188.5 | 112.6 | 93.4 | 112.3 | 6.94212E-05 |
| Q9EQ06 | Hsd17b11 | 90.8 | 93 | 93.4 | 92.3 | 92.5 | 91.7 | 112 | 121 | 118.6 | 97.3 | 97.1 | 100.2 | 8.44185E-05 |
| Q9CQS5 | Riok2 | 111 | 113.8 | 117 | 104.4 | 102.4 | 100 | 88.3 | 89.4 | 90.1 | 91.7 | 96.3 | 95.4 | 7.32331E-05 |
| Q8K1Z0 | Coq9 | 84.7 | 90.8 | 89.9 | 94.1 | 89.4 | 91.4 | 123.6 | 119.5 | 118.7 | 98.5 | 95.9 | 103.4 | 7.3578E-05 |
| Q99N84 | Mrps18b | 93.7 | 93.7 | 89.7 | 95 | 89.8 | 92.3 | 118.5 | 116.3 | 114.2 | 97.8 | 97.4 | 101.6 | 6.91539E-05 |
| Q8CCH2 | Nhlrc3 | 81.2 | 81.2 | 82.7 | 105.2 | 97 | 103 | 111.3 | 109.3 | 114.9 | 107.2 | 103.9 | 103.1 | 8.13629E-05 |
| Q9DAS9 | Gng12 | 88.1 | 87.8 | 87.3 | 100.6 | 100.1 | 98.6 | 109.8 | 106.7 | 110.5 | 105.2 | 99.9 | 105.5 | 7.98552E-05 |
| Q61048 | Wbp4 | 102.2 | 101.1 | 104.7 | 121.7 | 114.7 | 116.2 | 70.9 | 73 | 79.3 | 104.8 | 102.2 | 109.3 | 4.92243E-05 |
| Q8K0Z7 | Taco1 | 88.2 | 91 | 90.3 | 86.9 | 96 | 90.1 | 121.3 | 132.7 | 125.3 | 90.4 | 93.9 | 94 | 8.7241E-05 |
| A2AWP8 | Arhgef10l | 118.6 | 113.9 | 114.5 | 97.5 | 103.3 | 101.5 | 90.3 | 91.8 | 91.4 | 91.4 | 93 | 92.9 | 4.19587E-05 |
| Q3V1H1 | Ckap2 | 67.5 | 70.5 | 70.4 | 136.2 | 121.5 | 122.3 | 84.2 | 84.3 | 88 | 115.2 | 125.1 | 114.8 | 5.15026E-05 |
| Q9WVM1 | Racgap1 | 87.8 | 86.6 | 86.5 | 110.2 | 103.8 | 109.1 | 91.9 | 95.7 | 94.6 | 112.4 | 113.6 | 107.8 | 8.64579E-05 |
| Q31125 | Slc39a7 | 84.9 | 85.7 | 90.6 | 94.7 | 92.4 | 90.4 | 117.7 | 120.9 | 118.8 | 104 | 97.4 | 102.5 | 4.90078E-05 |
| P43883 | Plin2 | 55 | 56.5 | 57.6 | 82 | 79.1 | 79.7 | 163.1 | 169 | 160 | 99.3 | 96.8 | 101.9 | 1.61924E-06 |

| Protein Accession | Gene Name | Control-1 | Control-2 | Control-3 | APAP 100-1 | APAP 100-2 | APAP 100-3 | APAP 500-1 | APAP 500-2 | APAP 500-3 | APAP-HU-1 | APAP-HU-2 | APAP-HU-3 | Adjust pvalue |
|---|---|---|---|---|---|---|---|---|---|---|---|---|---|---|
| Q8R1G6 | Pdlim2 | 114.6 | 111.9 | 112.3 | 103.5 | 102.4 | 105.8 | 84.3 | 83.1 | 82.2 | 97.1 | 100.1 | 102.6 | 2.24605E-05 |
| Q9Z2Z6 | Slc25a20 | 84.3 | 80.7 | 80.3 | 92.8 | 91.6 | 91.1 | 124 | 134.5 | 123.4 | 100.1 | 96.6 | 100.6 | 4.08553E-05 |
| Q3UDF0 | Slc2a6 | 78 | 81.1 | 85.4 | 91.8 | 91.1 | 88.5 | 125.1 | 124.7 | 123.1 | 106.7 | 96.9 | 107.6 | 5.42103E-05 |
| Q9CPQ1 | Cox6c | 69.5 | 69.8 | 67.8 | 95.8 | 104.9 | 90.6 | 133.8 | 126 | 128.3 | 105.8 | 97.7 | 110 | 7.15723E-05 |
| Q80WR5 | | 117.1 | 120.4 | 120.3 | 100.3 | 97.2 | 100.7 | 90.3 | 79.5 | 83 | 97.5 | 97.2 | 96.6 | 7.3578E-05 |
| Q9CQ88 | Tspan31 | 84 | 85.3 | 83.9 | 89.3 | 82.8 | 89.1 | 132.7 | 135.2 | 125 | 97.9 | 97.6 | 97.1 | 2.38593E-05 |
| Q922H2 | Pdk3 | 90.1 | 90 | 92.9 | 89.1 | 91.8 | 94.7 | 118.2 | 125.3 | 122 | 94.1 | 94.6 | 97.1 | 3.50583E-05 |
| Q9WUZ9 | Entpd5 | 88.5 | 87.2 | 92.7 | 94.3 | 91.1 | 88.4 | 122.8 | 127.8 | 123.7 | 93.3 | 94.9 | 95.1 | 2.25608E-05 |
| Q9D600 | Gins2 | 101.6 | 104.1 | 104.6 | 122.4 | 111.5 | 113.5 | 76.1 | 74.7 | 79.9 | 100.6 | 105.9 | 105.2 | 8.3239E-05 |
| Q91WK1 | Spryd4 | 84.3 | 90.4 | 87.4 | 98.6 | 93.3 | 90.9 | 116.8 | 117.3 | 118 | 99.3 | 100 | 103.7 | 7.91683E-05 |
| Q9R1S3 | Pign | 80.8 | 77.4 | 83.3 | 94.6 | 95.6 | 92.4 | 122.5 | 130.3 | 129.7 | 94.7 | 96.5 | 102.3 | 3.46106E-05 |
| O35386 | Phyh | 117.5 | 115.7 | 119.3 | 94.5 | 98.6 | 98.1 | 92.4 | 96.9 | 94.9 | 87.1 | 94.3 | 90.7 | 7.54806E-05 |
| Q9D6K5 | Synj2bp | 77 | 79.5 | 82.1 | 97.1 | 94.7 | 91.5 | 124.8 | 122.2 | 122.6 | 104.1 | 98.4 | 105.9 | 2.47248E-05 |
| Q8BH79 | Ano10 | 84.7 | 75.8 | 86.7 | 80.8 | 89.9 | 80.7 | 139.2 | 154 | 140.7 | 86.4 | 91.5 | 89.6 | 5.15026E-05 |
| Q9CQ18 | Rnaseh2c | 112.8 | 125.2 | 125 | 120.6 | 105.2 | 118.1 | 62.9 | 63.5 | 64.5 | 104 | 98 | 100.2 | 8.64579E-05 |
| O35598 | Adam10 | 95.6 | 89.9 | 91.6 | 91.6 | 94.5 | 90.4 | 121.2 | 128.2 | 121.2 | 90.2 | 94.3 | 91.1 | 4.47401E-05 |
| Q9CRA5 | Golph3 | 111.9 | 112.8 | 117.7 | 100.5 | 99.9 | 98.3 | 92.5 | 91.4 | 89.3 | 94.5 | 96.4 | 94.8 | 4.90078E-05 |
| Q99J27 | Slc33a1 | 85.7 | 88 | 77.5 | 93.6 | 88.4 | 89.8 | 119.6 | 124.8 | 124.4 | 103.4 | 103.6 | 101.2 | 6.1428E-05 |
| Q8C263 | Ska3 | 67.8 | 69.1 | 67.9 | 102.9 | 110 | 109.6 | 100.2 | 113.2 | 113.1 | 118 | 116.6 | 111.7 | 7.39442E-05 |
| P50171 | Hsd17b8 | 78.8 | 80.8 | 79.9 | 90.5 | 87.1 | 85.4 | 141.5 | 134.6 | 127.8 | 101.1 | 95.3 | 97.3 | 2.92872E-05 |
| P0C6B7 | Igsf6 | 110.5 | 109.2 | 119 | 110.7 | 111.1 | 116.4 | 69.6 | 70.2 | 67.5 | 110.4 | 101.9 | 103.5 | 4.61361E-05 |
| Q9D2R6 | Coa3 | 68.5 | 70.9 | 65.8 | 102.1 | 98.2 | 91 | 131.3 | 122.8 | 128.7 | 110.4 | 100 | 110.2 | 5.36108E-05 |

| Protein Accession | Gene Name | Control-1 | Control-2 | Control-3 | APAP 100-1 | APAP 100-2 | APAP 100-3 | APAP 500-1 | APAP 500-2 | APAP 500-3 | APAP-HU-1 | APAP-HU-2 | APAP-HU-3 | Adjust pvalue |
|---|---|---|---|---|---|---|---|---|---|---|---|---|---|---|
| Q9ER41 | Tor1b | 82.1 | 82.7 | 85 | 93.8 | 90.6 | 89.3 | 130.8 | 127.9 | 120.2 | 100.3 | 98 | 99.3 | 3.05793E-05 |
| Q9DCC8 | Tomm20 | 85.6 | 87.8 | 87.1 | 97.3 | 101 | 100 | 113.7 | 110.1 | 113.4 | 102.6 | 98 | 103.5 | 5.99019E-05 |
| P11881 | Itpr1 | 113.8 | 100.5 | 104.2 | 77.7 | 80.8 | 77.2 | 136.6 | 136.8 | 129.4 | 79.6 | 80.9 | 82.5 | 2.76658E-05 |
| Q80TN4 | Dnajc16 | 85.3 | 82.3 | 89.1 | 95.7 | 96.9 | 95 | 119.5 | 123.5 | 122.8 | 95 | 95.2 | 99.8 | 2.70786E-05 |
| Q6PG16 | Hjurp | 71.5 | 65.9 | 71.1 | 125 | 129.5 | 130.8 | 85.5 | 87.3 | 88.8 | 107.1 | 120.3 | 117 | 2.54184E-05 |
| O55126 | Nipsnap 2 | 88.2 | 84 | 88.6 | 92.3 | 92.2 | 89.8 | 122.5 | 127.3 | 122.1 | 98.1 | 92.7 | 102.2 | 4.82617E-05 |
| O55125 | Nipsnap 1 | 86.5 | 85.9 | 87.8 | 93.2 | 90.6 | 90.9 | 120.4 | 128.1 | 125.8 | 97.5 | 94.2 | 99 | 2.07025E-05 |
| Q811J3 | Ireb2 | 121.5 | 126.8 | 127.4 | 101.8 | 103.7 | 105.4 | 76.6 | 79.4 | 83.7 | 88 | 92.3 | 93.5 | 2.38593E-05 |
| P0DOV2 | Ifi204 | 87 | 88.1 | 82.3 | 112.8 | 106.5 | 106.2 | 90.5 | 89.1 | 88 | 116.2 | 114.4 | 118.9 | 4.47401E-05 |
| Q61703 | Itih2 | 46.3 | 48.5 | 46.8 | 59.6 | 60 | 56.2 | 154.2 | 196 | 178.3 | 125.6 | 99.9 | 128.6 | 8.72476E-05 |
| Q9D3B1 | Hacd2 | 68.2 | 69.2 | 73.6 | 86.1 | 99.7 | 83.5 | 134.5 | 145.5 | 141.7 | 95.6 | 98.4 | 103.9 | 5.2417E-05 |
| Q6RUT7 | Ccsmst1 | 70.8 | 76.4 | 77.8 | 95.4 | 93.2 | 89.1 | 127.5 | 127.3 | 130.6 | 102.7 | 98.1 | 111.2 | 4.67397E-05 |
| P61804 | Dad1 | 76.6 | 79.1 | 80.6 | 88.8 | 92.8 | 88.8 | 119.2 | 120.8 | 131.3 | 112.5 | 102.9 | 106.6 | 8.70273E-05 |
| Q91WA1 | Tipin | 104.8 | 115.4 | 110.9 | 132.2 | 117.4 | 133.3 | 54.9 | 51.6 | 61 | 108.2 | 105.8 | 104.5 | 4.61936E-05 |
| Q9D975 | Srxn1 | 74.9 | 80.6 | 75.9 | 95.2 | 94.6 | 88.9 | 129.3 | 125.6 | 122.2 | 107.4 | 100.7 | 104.8 | 2.95731E-05 |
| P12023 | App | 77.7 | 82.7 | 76.3 | 97.1 | 95.9 | 95.5 | 119.1 | 122 | 119 | 109.2 | 99.8 | 105.7 | 4.05129E-05 |
| Q3UA16 | Spc25 | 94.1 | 97 | 98.7 | 117.4 | 111.4 | 112.6 | 81.7 | 86 | 87.7 | 106 | 102.8 | 104.7 | 7.93899E-05 |
| B2RWS6 | Ep300 | 116.8 | 109.9 | 110.2 | 125.6 | 116 | 117.3 | 73.2 | 67.8 | 77.6 | 90 | 96.1 | 99.5 | 9.95431E-05 |
| P56390 | Cks2 | 85.5 | 81.1 | 77.1 | 126.7 | 127.3 | 119.4 | 74.7 | 73.4 | 78.6 | 118.2 | 115.5 | 122.3 | 2.24605E-05 |
| Q8BFY7 | Pimreg | 89.2 | 96.8 | 89 | 121.9 | 118.4 | 119.3 | 75.1 | 73 | 73.5 | 113.2 | 118.3 | 112.3 | 1.76997E-05 |
| Q62313 | Tgoln1 | 60.6 | 66.9 | 59.5 | 101.4 | 91 | 90.9 | 139 | 128.5 | 138.1 | 109.8 | 102.6 | 111.7 | 3.49561E-05 |

| Protein Accession | Gene Name | Control-1 | Control-2 | Control-3 | APAP 100-1 | APAP 100-2 | APAP 100-3 | APAP 500-1 | APAP 500-2 | APAP 500-3 | APAP-HU-1 | APAP-HU-2 | APAP-HU-3 | Adjust pvalue |
|---|---|---|---|---|---|---|---|---|---|---|---|---|---|---|
| Q8C6I2 | Sdhaf2 | 76.8 | 77.7 | 79.6 | 93 | 91.3 | 96.3 | 125.8 | 121.4 | 127.6 | 104.2 | 97.7 | 108.6 | 4.01765E-05 |
| O35955 | Psmb10 | 106.1 | 104 | 107.6 | 109 | 107.3 | 108.3 | 77 | 85.1 | 85.5 | 103.1 | 104.4 | 102.5 | 9.21497E-05 |
| Q3URE1 | Acsf3 | 88.7 | 91.9 | 92.3 | 94.5 | 92.2 | 91.2 | 115.6 | 121.6 | 120.5 | 97.3 | 98.1 | 96.2 | 2.76658E-05 |
| Q9CZL5 | Pcbd2 | 85.9 | 87.7 | 90.8 | 96.5 | 93.6 | 93 | 116.6 | 118 | 117 | 100.5 | 97.8 | 102.4 | 2.58974E-05 |
| Q8R0F8 | Fahd1 | 98.7 | 100.1 | 103.5 | 81.8 | 78.5 | 81 | 126.9 | 131.8 | 125.8 | 94.8 | 85.6 | 91.4 | 2.53608E-05 |
| A1L314 | Mpeg1 | 122.8 | 119.5 | 125.1 | 104.9 | 101.4 | 98.2 | 75.4 | 77.6 | 78.3 | 99.7 | 99.5 | 97.5 | 1.63466E-05 |
| O35316 | Slc6a6 | 146.7 | 142.7 | 150.3 | 96.3 | 100.2 | 103.3 | 65.1 | 67.3 | 69.2 | 84.5 | 86.3 | 88.1 | 2.35904E-06 |
| O55003 | Bnip3 | 156.7 | 158.6 | 164.7 | 105.3 | 101.8 | 102.2 | 50 | 60 | 58.4 | 78.4 | 81.6 | 82.4 | 2.20093E-06 |
| Q8VC04 | Tmem106a | 79.1 | 82 | 84.5 | 111.5 | 113.3 | 111.1 | 88.2 | 96.9 | 95.8 | 110.2 | 114.7 | 112.7 | 6.15936E-05 |
| P15702 | Spn | 76.9 | 77 | 77.9 | 130.6 | 112.9 | 118.1 | 80.2 | 82.9 | 86.6 | 117.3 | 117.3 | 122.2 | 8.44185E-05 |
| Q8K370 | Acad10 | 87.9 | 89.4 | 91 | 96.3 | 98.7 | 91.6 | 112.2 | 117.7 | 115.4 | 100.1 | 100.6 | 98.9 | 8.99377E-05 |
| Q8CIM3 | D2hgdh | 84.7 | 88 | 83.7 | 88.8 | 85.8 | 90.4 | 129.6 | 131.2 | 125.5 | 100.9 | 94.3 | 97.2 | 1.76997E-05 |
| Q08297 | Rad51 | 80.9 | 84 | 81.6 | 106.6 | 103.6 | 102.3 | 110.8 | 109.6 | 106.6 | 106.1 | 103.1 | 104.9 | 2.58974E-05 |
| Q8C0L8 | Cog5 | 89.8 | 82.4 | 91.3 | 84.5 | 89.8 | 87.9 | 127.3 | 130.5 | 131.6 | 89.6 | 96.8 | 98.5 | 4.63182E-05 |
| Q9D6V8 | Paip2 | 85.1 | 83.2 | 81.8 | 132.9 | 120.6 | 130.8 | 76.5 | 67.5 | 72.9 | 120.3 | 116.8 | 111.7 | 3.63605E-05 |
| P43024 | Cox6a1 | 79.1 | 80.1 | 82.5 | 100.1 | 99.5 | 93 | 116.6 | 117.5 | 114.6 | 108 | 102.3 | 106.7 | 4.19587E-05 |
| P36371 | Tap2 | 90.3 | 88.7 | 89.4 | 94.4 | 95.3 | 96.3 | 115.4 | 120.5 | 118.7 | 94.8 | 98.2 | 98.2 | 1.76997E-05 |
| Q9DCA2 | Mrps11 | 93.2 | 93.9 | 91 | 91.1 | 90.9 | 91.8 | 112.4 | 120.7 | 118.6 | 96.4 | 99.3 | 100.6 | 7.3578E-05 |
| E9Q5F9 | Setd2 | 110.3 | 103.7 | 108.3 | 112.3 | 110.3 | 109.9 | 77.7 | 79.1 | 82.8 | 97.9 | 104.1 | 103.6 | 6.33677E-05 |
| Q91ZW2 | Pofut1 | 88.7 | 90.9 | 94.4 | 87.9 | 96.4 | 93.5 | 123.2 | 125.3 | 117.4 | 94.3 | 94.1 | 94 | 9.47877E-05 |
| Q9D9Z1 | Knstrn | 103.1 | 104.3 | 104.6 | 132.4 | 120.2 | 127.9 | 61.5 | 67.2 | 69.5 | 100.2 | 103.7 | 105.5 | 2.58974E-05 |
| Q9CR10 | Oxld1 | 84.4 | 89.4 | 89.9 | 91.2 | 82 | 83.8 | 128.2 | 131.9 | 135.2 | 94.3 | 94.3 | 95.3 | 2.57497E-05 |

| Protein Accession | Gene Name | Control-1 | Control-2 | Control-3 | APAP 100-1 | APAP 100-2 | APAP 100-3 | APAP 500-1 | APAP 500-2 | APAP 500-3 | APAP-HU-1 | APAP-HU-2 | APAP-HU-3 | Adjust pvalue |
|---|---|---|---|---|---|---|---|---|---|---|---|---|---|---|
| Q9CYW4 | Hdhd3 | 75.3 | 81.8 | 81.3 | 95.7 | 86.3 | 82.6 | 125.1 | 137.1 | 136.1 | 100 | 98.4 | 100.4 | 8.29599E-05 |
| Q922B1 | Macrod1 | 94.1 | 90.4 | 99.2 | 84.3 | 82.9 | 82.8 | 127.3 | 133.9 | 126.3 | 89.9 | 94.6 | 94.3 | 2.95731E-05 |
| Q3V1T4 | P3h1 | 77.1 | 79.2 | 78.1 | 86.2 | 89.3 | 93.2 | 126.3 | 139.1 | 135.2 | 96.2 | 99.3 | 100.9 | 2.95731E-05 |
| Q9CR86 | Carhsp1 | 74.2 | 74.8 | 77 | 96.1 | 98.4 | 95 | 122.6 | 127.2 | 131.8 | 103 | 99.8 | 100.2 | 1.50383E-05 |
| Q3UUI3 | Them4 | 86.1 | 88.3 | 87.8 | 92.4 | 87.1 | 90.1 | 127.6 | 128.7 | 132.6 | 91.2 | 94.3 | 93.8 | 6.85351E-06 |
| Q9D023 | Mpc2 | 71.3 | 78.9 | 77.3 | 93.8 | 94.4 | 90 | 125.1 | 129.6 | 131.1 | 99.8 | 98.6 | 110.1 | 4.92042E-05 |
| Q91VE0 | Slc27a4 | 90.9 | 82.6 | 89.9 | 79.9 | 92 | 89.7 | 132.5 | 136.7 | 130.6 | 86.6 | 93 | 95.7 | 8.08272E-05 |
| Q7TMF2 | Eri1 | 130.1 | 126.9 | 138.1 | 100.7 | 94.4 | 102.6 | 82.9 | 81.5 | 79 | 84.3 | 92.5 | 86.9 | 4.8877E-05 |
| P47930 | Fosl2 | 105.3 | 100.2 | 105.6 | 114.6 | 110 | 109.4 | 73.8 | 78 | 81.9 | 104.3 | 108.2 | 108.7 | 7.00366E-05 |
| Q9WTX2 | Prkra | 86.4 | 84.8 | 86.8 | 93 | 91.6 | 91.2 | 126.1 | 124.5 | 123 | 95.8 | 98.3 | 98.6 | 1.9039E-06 |
| O70201 | Birc5 | 82.6 | 85.6 | 88.3 | 125.9 | 119.1 | 123.8 | 76 | 83.2 | 80 | 116.3 | 113.7 | 105.4 | 5.37986E-05 |
| Q3UDW8 | Hgsnat | 82.2 | 69.7 | 80.5 | 79.9 | 90.2 | 81.1 | 146.7 | 170.1 | 150.9 | 80.2 | 82.3 | 86.2 | 7.00366E-05 |
| Q9CZI9 | Aen | 90.1 | 89.9 | 88.2 | 107.7 | 104 | 106.8 | 98.7 | 99.7 | 102.1 | 103.3 | 104.2 | 105.2 | 5.67837E-05 |
| P63030 | Mpc1 | 74.9 | 76.1 | 80.9 | 98 | 92.1 | 89.4 | 127.5 | 130.5 | 131.2 | 99 | 95.4 | 105.1 | 3.49561E-05 |
| Q7TSY8 | Sgo2 | 132.6 | 132.6 | 127.7 | 113 | 107.1 | 115 | 65.9 | 56.8 | 59.3 | 98.3 | 96.6 | 95.2 | 1.09895E-05 |
| P22518 | Clk1 | 65.3 | 69 | 64 | 72.1 | 77.7 | 84.6 | 164.6 | 155.3 | 146.1 | 103.3 | 98.8 | 99 | 2.24372E-05 |
| Q9ERH4 | Nusap1 | 84.1 | 88.8 | 82.7 | 132.9 | 121.1 | 124.1 | 76.5 | 77.6 | 80.6 | 108.4 | 114.3 | 109 | 4.05129E-05 |
| Q3U2A8 | Vars2 | 102.8 | 98.2 | 104.4 | 84.9 | 89.3 | 86.7 | 126.4 | 120.4 | 123.4 | 82.9 | 90.3 | 90.4 | 4.97766E-05 |
| Q9CZN8 | Qrsl1 | 94.5 | 92.2 | 92.5 | 98.9 | 92.6 | 92.7 | 112.1 | 115.9 | 115.5 | 97.2 | 97.4 | 98.6 | 9.2914E-05 |
| Q8CFV9 | Rfk | 73.5 | 77.7 | 84.8 | 92.8 | 99.9 | 93.7 | 125.6 | 127.3 | 132.7 | 96.5 | 97 | 98.5 | 4.82617E-05 |
| Q3UHH1 | Zswim8 | 86.9 | 87 | 85.9 | 86.2 | 94.5 | 90.1 | 119.1 | 125.8 | 121.5 | 99.6 | 99.4 | 103.9 | 4.61936E-05 |
| P97783 | Mllt11 | 57.6 | 67.3 | 56.7 | 83 | 68.1 | 66 | 115.7 | 107.3 | 120.3 | 155.2 | 157.9 | 144.8 | 3.51792E-05 |
| Q9CR46 | Ska2 | 66.2 | 66.8 | 68.9 | 124.8 | 111.8 | 116.6 | 86 | 88.1 | 92.7 | 124.9 | 124.1 | 128.9 | 2.22534E-05 |

| Protein Accession | Gene Name | Control-1 | Control-2 | Control-3 | APAP 100-1 | APAP 100-2 | APAP 100-3 | APAP 500-1 | APAP 500-2 | APAP 500-3 | APAP-HU-1 | APAP-HU-2 | APAP-HU-3 | Adjust pvalue |
|---|---|---|---|---|---|---|---|---|---|---|---|---|---|---|
| Q8C0L0 | Tmx4 | 75.5 | 81.6 | 82 | 90.4 | 87.3 | 91.4 | 129.9 | 133.8 | 133.4 | 99.6 | 96.6 | 98.4 | 7.18133E-06 |
| Q9CXY9 | Pigk | 90 | 81.4 | 88.5 | 93.3 | 91.9 | 91.5 | 124.1 | 132.5 | 120.6 | 96 | 94.2 | 96 | 8.76837E-05 |
| Q8VD57 | Sft2d2 | 73.8 | 77.3 | 78.7 | 92 | 97.5 | 90.9 | 127.3 | 129.2 | 132 | 96 | 100.8 | 104.5 | 2.22534E-05 |
| Q8BH51 | Cox14 | 74.8 | 72.1 | 76.5 | 91.5 | 102 | 88.6 | 144.2 | 137.4 | 139.2 | 90.9 | 89.1 | 93.6 | 2.26247E-05 |
| Q9D0P0 | Ebpl | 76.5 | 74.2 | 77.2 | 86.9 | 90.9 | 89.7 | 136.9 | 138.8 | 129.9 | 100.8 | 94.9 | 103.3 | 1.756E-05 |
| Q00623 | Apoa1 | 45 | 46.3 | 44.5 | 49.4 | 52.3 | 52.8 | 155.8 | 190.1 | 170.8 | 145 | 108.6 | 139.4 | 7.24382E-05 |
| Q99MK9 | Rassf1 | 73.6 | 72.5 | 72.7 | 119.5 | 126.1 | 116.4 | 82.8 | 95.4 | 93.4 | 111.7 | 117.4 | 118.5 | 6.54149E-05 |
| P51943 | Ccna2 | 75.8 | 79.8 | 75 | 126.7 | 124.2 | 126.4 | 76.3 | 80.9 | 87.6 | 116.6 | 121.5 | 109.4 | 4.16232E-05 |
| Q8CAK1 | Iba57 | 67.9 | 66.4 | 72 | 84.7 | 89.7 | 81.1 | 139.6 | 164 | 158.4 | 89.1 | 94.2 | 92.9 | 4.65824E-05 |
| Q8BHD8 | Pcmtd2 | 84.3 | 79.6 | 90.7 | 79.3 | 85.9 | 82.3 | 138.7 | 149.8 | 139.6 | 85.7 | 93.1 | 91 | 3.25629E-05 |
| Q8BJ56 | Pnpla2 | 72.8 | 75.7 | 87.4 | 72 | 80.8 | 75.4 | 154.5 | 173.4 | 158.6 | 74.4 | 86 | 89 | 4.47401E-05 |
| B1B212 | H60b | 65.7 | 66.8 | 69.7 | 119.6 | 108.3 | 110.2 | 93.5 | 91.2 | 91.1 | 136.9 | 117.9 | 129.1 | 8.54099E-05 |
| Q3TC33 | Ccdc127 | 74.1 | 73.7 | 87.8 | 88.6 | 88.8 | 88.1 | 147.2 | 135 | 132.5 | 94.3 | 92.6 | 97.4 | 8.25969E-05 |
| Q9CX66 | D10Wsu102e | 97.4 | 94 | 97.9 | 130.5 | 120.4 | 121.7 | 72.7 | 77.3 | 79.9 | 103.9 | 104.5 | 99.8 | 4.65824E-05 |
| Q9DAC7 | Ttc32 | 109.3 | 111.2 | 111.6 | 118.7 | 107.8 | 116.1 | 72.6 | 69.5 | 75.3 | 103.4 | 100.2 | 104.3 | 4.16232E-05 |
| Q8VDV3 | Rab3il1 | 129.5 | 127.8 | 129.6 | 98.2 | 92.4 | 97.9 | 85.6 | 87.3 | 89.3 | 83.4 | 91.5 | 87.6 | 1.96502E-05 |
| Q9CRY7 | Gdpd1 | 77.9 | 72.7 | 85 | 91.5 | 96.8 | 90.8 | 125.7 | 140.3 | 132.9 | 92.8 | 96.6 | 97.2 | 9.19668E-05 |
| Q3UMR5 | Mcu | 92.3 | 84.7 | 87 | 87.9 | 91.3 | 89.8 | 122.8 | 128.2 | 121.4 | 99.1 | 98.2 | 97.4 | 3.16777E-05 |
| Q61070 | Ei24 | 77 | 73.8 | 81.8 | 88.1 | 89.4 | 86.7 | 144.5 | 137.7 | 131.3 | 95.8 | 96.1 | 97.6 | 2.24605E-05 |
| Q9JKK7 | Tmod2 | 45.5 | 47.2 | 47.1 | 57.1 | 61.3 | 58.2 | 174.9 | 198.5 | 175.3 | 122.7 | 91.5 | 120.6 | 4.06613E-05 |
| Q9Z1X9 | Cdc45 | 61.8 | 60.8 | 59.6 | 67.8 | 69.4 | 61.7 | 140.7 | 157.3 | 162.9 | 131.4 | 107 | 119.6 | 5.24253E-05 |
| Q8BG21 | Cacfd1 | 84.2 | 80.7 | 82.6 | 81.4 | 86.8 | 89.6 | 135.3 | 139.8 | 130.3 | 99.3 | 92.9 | 97 | 2.33456E-05 |

| Protein Accession | Gene Name | Control-1 | Control-2 | Control-3 | APAP 100-1 | APAP 100-2 | APAP 100-3 | APAP 500-1 | APAP 500-2 | APAP 500-3 | APAP-HU-1 | APAP-HU-2 | APAP-HU-3 | Adjust pvalue |
|---|---|---|---|---|---|---|---|---|---|---|---|---|---|---|
| P98197 | Atp11a | 74.9 | 75.9 | 76.5 | 94.9 | 101.4 | 92.2 | 125.4 | 122 | 125.2 | 105.3 | 99.7 | 106.6 | 2.61375E-05 |
| Q91V16 | Etfrf1 | 47.9 | 51.8 | 50 | 86.9 | 82.6 | 81.5 | 160.1 | 146.1 | 159.6 | 114.9 | 101.1 | 117.4 | 1.76997E-05 |
| A2A559 | Pgap3 | 77.3 | 81.8 | 81.4 | 86.6 | 85.4 | 86.1 | 128.9 | 137.1 | 125.9 | 100.9 | 100.7 | 108 | 3.09416E-05 |
| Q9Z1T6 | Pikfyve | 85.5 | 81.2 | 98.2 | 123.4 | 128.6 | 128.8 | 73.9 | 73.5 | 77.7 | 107.4 | 113 | 108.9 | 8.38065E-05 |
| Q9DCI3 | Stard3nl | 75.2 | 75.9 | 82.6 | 101.7 | 95.6 | 94.3 | 127.3 | 122.2 | 119.8 | 103.1 | 99.8 | 102.5 | 4.8877E-05 |
| Q5NCE8 | Mrs2 | 89.5 | 87.2 | 85.7 | 100.7 | 98.8 | 100.1 | 112.9 | 118.3 | 112.4 | 99.1 | 94.9 | 100.4 | 8.23222E-05 |
| O88939 | Zbtb7a | 125.6 | 125.6 | 138.3 | 100.1 | 101.3 | 106.7 | 68.4 | 70.8 | 74.3 | 96.7 | 97.8 | 94.4 | 4.29071E-05 |
| Q9CQ00 | Dmac1 | 70.7 | 80.1 | 76.4 | 90.3 | 91.8 | 83.5 | 140.6 | 136.7 | 138 | 99.1 | 92.7 | 100.1 | 2.05287E-05 |
| Q9D818 | Sapcd2 | 139 | 148.2 | 151.4 | 98.7 | 107.2 | 93.4 | 54.7 | 70.4 | 62.8 | 96.4 | 93.3 | 84.5 | 5.8088E-05 |
| Q14CH7 | Aars2 | 86.9 | 84.7 | 87.8 | 88.7 | 86.6 | 87.2 | 124.9 | 140.4 | 127.8 | 92 | 95.8 | 97.3 | 7.31812E-05 |
| Q60611 | Satb1 | 92.6 | 100.1 | 101.4 | 80.2 | 76.2 | 76.5 | 123.3 | 115.3 | 119.6 | 109 | 101.1 | 104.8 | 8.72476E-05 |
| Q920Q8 | Ivns1abp | 147.9 | 138.7 | 146.3 | 81.4 | 91.1 | 97.6 | 83.4 | 82.7 | 81.4 | 78.4 | 90.4 | 80.8 | 5.12469E-05 |
| Q9DBH0 | Wwp2 | 150.7 | 130 | 148.2 | 89.6 | 95.7 | 94.8 | 79.2 | 81 | 78.6 | 81.2 | 82.6 | 88.3 | 7.3578E-05 |
| O09126 | Sema4d | 138.5 | 143 | 137.1 | 107.7 | 110.1 | 106.3 | 52.9 | 54.3 | 58.7 | 94.5 | 97.1 | 99.9 | 1.9039E-06 |
| Q8BZ36 | Rint1 | 107.4 | 105.4 | 106.7 | 103 | 102.7 | 100.6 | 92.1 | 94.8 | 94.6 | 97.1 | 98.8 | 96.8 | 9.6261E-05 |
| Q99PL7 | Scd3 | 148.5 | 140.7 | 153.5 | 98.5 | 111.2 | 107.5 | 57.9 | 68 | 67.4 | 80.8 | 84.4 | 81.7 | 2.38593E-05 |
| Q01339 | Apoh | 16.6 | 17.1 | 11.6 | 23.8 | 31.7 | 25.9 | 248.7 | 275.8 | 210.1 | 130.9 | 95.5 | 112.3 | 4.05129E-05 |
| P60603 | Romo1 | 76.1 | 75.6 | 81.8 | 89.2 | 95.4 | 89.1 | 131.3 | 133.8 | 131.6 | 102.3 | 96.6 | 97.1 | 1.69427E-05 |
| Q8K2T4 | Uqcc3 | 78.6 | 79.7 | 84 | 93 | 97.7 | 85.9 | 119.7 | 117.6 | 122 | 109.3 | 104.5 | 108 | 7.93899E-05 |
| Q9JKY5 | Hip1r | 76.2 | 83.2 | 71.8 | 133.6 | 140.9 | 124.3 | 77.3 | 69.3 | 83.3 | 114.4 | 111.5 | 114.2 | 8.88647E-05 |

**Table S2 Localization of differentially expressed proteins in macrophages**

| Protein Accession No. | Location | MW [kDa] | calc. pI |
|---|---|---|---|
| Q8BTM8 | cytoplasm；extracellular | 281 | 6.04 |
| E9PVX6 | nucleus | 350.7 | 9.72 |
| P16546 | cytoplasm；cell membrane；cytoskeleton | 284.4 | 5.33 |
| P09103 | cell membrane；endoplasmic reticulum | 57 | 4.88 |
| P23116 | cytoplasm；nucleus | 161.8 | 6.77 |
| Q6P5E4 | endoplasmic reticulum；Golgi apparatus | 176.3 | 5.62 |
| Q8CAQ8 | mitochondria | 83.8 | 6.61 |
| Q8VHX6 | cell membrane；cytoskeleton | 290.9 | 5.95 |
| Q03265 | cell membrane；mitochondria | 59.7 | 9.19 |
| Q01320 | cytoplasm；nucleus | 172.7 | 8.6 |
| Q8BFR5 | mitochondria | 49.5 | 7.56 |
| Q91YQ5 | cytoplasm；endoplasmic reticulum | 68.5 | 6.46 |
| Q3U0V1 | cytoplasm；nucleus | 76.7 | 7.33 |
| Q8CGK3 | cytoplasm；mitochondria | 105.8 | 6.57 |
| P49710 | mitochondria | 54.2 | 4.84 |
| P26443 | mitochondria；endoplasmic reticulum | 61.3 | 8 |
| Q9CQN1 | nucleus；mitochondria | 80.2 | 6.68 |
| Q7TMK9 | cytoplasm；nucleus；endoplasmic reticulum | 69.6 | 8.59 |
| Q922J3 | cytoplasm；nucleus；cytoskeleton | 155.7 | 5.24 |
| Q9QZQ1 | cytoplasm；cell membrane；nucleus | 206.4 | 6.32 |
| P30416 | cytoplasm；nucleus；mitochondria；cytoskeleton | 51.5 | 5.72 |
| P61979 | cytoplasm；nucleus | 50.9 | 5.54 |
| P18242 | extracellular；mitochondria；lysosomes | 44.9 | 7.15 |
| Q8BG05 | cytoplasm；nucleus | 39.6 | 9.01 |
| Q99MR6 | cytoplasm；nucleus | 100.4 | 5.97 |
| P08249 | cytoplasm；mitochondria | 35.6 | 8.68 |
| Q9EQP2 | cell membrane；endoplasmic reticulum | 61.4 | 6.76 |
| Q9Z2I8 | cell membrane；mitochondria | 46.8 | 7.02 |
| O70318 | cytoplasm；cell membrane；cytoskeleton | 109.9 | 5.43 |
| Q8VIJ6 | cytoplasm；nucleus | 75.4 | 9.44 |
| Q8BHN3 | endoplasmic reticulum；Golgi | 106.8 | 6.06 |

| | | | |
|---|---|---|---|
| | apparatus | | |
| Q60597 | nucleus；mitochondria | 116.4 | 6.83 |
| Q04447 | cytoplasm；extracellular；mitochondria | 42.7 | 5.67 |
| Q9Z1Z2 | cytoplasm；nucleus | 38.4 | 5.12 |
| Q6A068 | cytoplasm；nucleus | 92.1 | 8.02 |
| P10605 | cytoplasm；cell membrane；extracellular；lysosomes | 37.3 | 5.91 |
| Q99L45 | cytoplasm | 38.1 | 5.8 |
| P30681 | cytoplasm；extracellular；nucleus | 24.1 | 7.31 |
| P47738 | mitochondria | 56.5 | 7.62 |
| Q99LC5 | cytoplasm；mitochondria | 35 | 8.38 |
| Q8QZT1 | mitochondria | 44.8 | 8.51 |
| Q8BWT1 | mitochondria | 41.8 | 8.09 |
| Q8K1B8 | | 75.6 | 7.05 |
| Q60932 | cell membrane；mitochondria | 32.3 | 8.43 |
| Q6A028 | cytoplasm；cell membrane；nucleus；cytoskeleton | 69 | 6.05 |
| Q9CVB6 | cytoplasm；nucleus；cytoskeleton | 34.3 | 7.36 |
| Q6GQT9 | endoplasmic reticulum | 133.3 | 6.09 |
| Q91WQ3 | cytoplasm；nucleus | 59.1 | 7.01 |
| P63158 | cytoplasm；cell membrane；extracellular；nucleus | 24.9 | 5.74 |
| Q8QZY1 | cytoplasm；nucleus | 66.6 | 6.44 |
| P17918 | nucleus | 28.8 | 4.77 |
| Q7TSC1 | cytoplasm；nucleus | 229.1 | 9.39 |
| P05202 | cell membrane；mitochondria | 47.4 | 9 |
| Q99K85 | cytoplasm | 40.4 | 8.03 |
| P54071 | cytoplasm；mitochondria | 50.9 | 8.69 |
| P67778 | cytoplasm；cell membrane；nucleus；mitochondria | 29.8 | 5.76 |
| P68510 | cytoplasm；cell membrane | 28.2 | 4.89 |
| P62814 | cytoplasm；cell membrane | 56.5 | 5.81 |
| Q9CZU6 | mitochondria | 51.7 | 8.57 |
| P51881 | mitochondria | 32.9 | 9.73 |
| Q9R1T2 | cytoplasm；nucleus | 38.6 | 5.36 |
| P11157 | cytoplasm；nucleus | 45.1 | 5.45 |
| P21550 | cytoplasm；cell membrane | 47 | 7.18 |
| Q8BH59 | mitochondria | 74.5 | 8.25 |
| Q99L47 | cytoplasm | 41.6 | 5.26 |
| O35857 | mitochondria | 51.1 | 8.13 |
| Q8BH04 | cytoplasm；mitochondria | 70.5 | 7.28 |
| P50544 | nucleus；mitochondria | 70.8 | 8.75 |

| | | | |
|---|---|---|---|
| Q8BK64 | cytoplasm；endoplasmic reticulum | 38.1 | 5.53 |
| Q9WVA4 | | 22.4 | 8.24 |
| Q91VH2 | cytoplasm；cell membrane；Golgi apparatus | 66.5 | 5.52 |
| Q922U1 | cytoplasm； nucleus | 77.4 | 9.5 |
| P25976 | nucleus | 89.5 | 5.76 |
| Q8R180 | endoplasmic reticulum | 54.1 | 6.54 |
| Q60668 | cytoplasm； nucleus | 38.3 | 7.81 |
| D3Z7P3 | cytoplasm； mitochondria | 73.9 | 7.99 |
| Q9DCN2 | cytoplasm； mitochondria； endoplasmic reticulum | 34.1 | 8.38 |
| Q8BI84 | mitochondria； endoplasmic reticulum | 213.5 | 4.75 |
| Q6A065 | cytoplasm； cytoskeleton | 174.9 | 7.17 |
| O35841 | cytoplasm； nucleus | 56.8 | 5.92 |
| Q60865 | cytoplasm | 78.1 | 5.25 |
| P48722 | cytoplasm； nucleus | 94.3 | 5.74 |
| Q07076 | cytoplasm； cell membrane； nucleus | 49.9 | 6.18 |
| Q6P9P6 | cytoplasm； nucleus | 118 | 6 |
| Q925I1 | mitochondria | 66.7 | 9.29 |
| P29758 | cytoplasm； nucleus； mitochondria； cytoskeleton | 48.3 | 6.62 |
| Q8K297 | endoplasmic reticulum | 71 | 7.28 |
| Q810A7 | cytoplasm； nucleus | 101.9 | 6.98 |
| P10711 | nucleus | 33.9 | 8.38 |
| Q8C1B7 | cytoplasm； cytoskeleton | 49.7 | 6.68 |
| Q8K298 | cytoplasm；nucleus；cytoskeleton | 122.7 | 6.98 |
| Q91VX2 | cytoplasm； nucleus | 117.9 | 7.72 |
| Q8BNW9 | | 67.9 | 5.39 |
| O08808 | cytoplasm； cell membrane； nucleus； cytoskeleton | 139.3 | 5.57 |
| Q9DCX2 | mitochondria | 18.7 | 5.69 |
| Q99KQ4 | cytoplasm； cell membrane； extracellular； nucleus | 55.4 | 7.15 |
| Q99JY0 | mitochondria； endoplasmic reticulum | 51.4 | 9.38 |
| Q922Q8 | endoplasmic reticulum | 34.9 | 9.52 |
| P46664 | cytoplasm； mitochondria | 50 | 6.38 |
| P11440 | cytoplasm； nucleus； mitochondria； cytoskeleton | 34.1 | 8.43 |
| Q9D051 | cytoplasm； mitochondria | 38.9 | 6.87 |
| Q5SSZ5 | cytoplasm | 155.5 | 6.65 |

| | | | |
|---|---|---|---|
| Q9CQW2 | cytoplasm；lysosomes | 21.5 | 8.43 |
| A2APB8 | cytoplasm；extracellular；nucleus | 85.8 | 9.38 |
| Q5SXY1 | cytoplasm；cell membrane；nucleus | 118 | 6.64 |
| P51174 | mitochondria | 47.9 | 8.31 |
| Q9ESZ8 | cytoplasm；nucleus | 112.2 | 6.55 |
| P23198 | nucleus | 20.8 | 5.22 |
| Q9DCW4 | mitochondria | 27.6 | 8.1 |
| Q9D0F3 | endoplasmic reticulum；Golgi apparatus | 57.8 | 6.34 |
| Q6NZF1 | | 86.4 | 8.13 |
| P10404 | cell membrane | 69.6 | 7.96 |
| O08795 | endoplasmic reticulum | 58.8 | 4.46 |
| Q9Z2I9 | mitochondria | 50.1 | 7.01 |
| Q9DBH5 | extracellular；Golgi apparatus | 40.4 | 6.95 |
| Q3TIV5 | cytoplasm；nucleus | 48.3 | 5.3 |
| P98078 | cytoplasm；cell membrane；nucleus | 82.3 | 6.1 |
| Q8BVE3 | | 55.8 | 6.61 |
| Q9DC61 | mitochondria | 58.2 | 6.83 |
| Q60930 | mitochondria | 31.7 | 7.49 |
| Q9CYN2 | endoplasmic reticulum | 25 | 8.57 |
| P32020 | cytoplasm；mitochondria；endoplasmic reticulum | 59.1 | 7.44 |
| Q8C129 | cytoplasm；cell membrane | 117.2 | 5.96 |
| Q6PGB6 | cytoplasm；nucleus | 19.4 | 8.81 |
| P35979 | cytoplasm；nucleus | 17.8 | 9.42 |
| Q3UKJ7 | cytoplasm；nucleus | 57.5 | 7.18 |
| P59328 | nucleus | 124.2 | 5.63 |
| Q60715 | mitochondria；endoplasmic reticulum | 60.9 | 5.9 |
| O35639 | cytoplasm；cell membrane | 36.4 | 5.76 |
| P39749 | nucleus；mitochondria | 42.3 | 8.34 |
| Q8VEH3 | cytoplasm；cytoskeleton；lysosomes | 21.4 | 7.77 |
| Q9Z130 | cytoplasm；nucleus | 33.5 | 7.31 |
| Q9JJU8 | | 12.8 | 4.92 |
| P59325 | cytoplasm；cell membrane | 48.9 | 5.52 |
| P97377 | cytoplasm；nucleus；cytoskeleton | 39 | 8.54 |
| Q00547 | cytoplasm；cytoskeleton | 91.7 | 5.52 |
| Q920E5 | cytoplasm | 40.6 | 5.66 |
| Q9EQH2 | endoplasmic reticulum | 106.5 | 6.2 |
| Q8R5A3 | cell membrane；cytoskeleton | 74.3 | 5.35 |

| | | | |
|---|---|---|---|
| Q9D2G2 | nucleus；mitochondria | 49 | 8.95 |
| P28650 | cytoplasm | 50.2 | 8.43 |
| Q61941 | mitochondria | 113.8 | 7.64 |
| Q8VEM8 | mitochondria | 39.6 | 9.26 |
| Q922Q4 | cytoplasm；mitochondria | 33.6 | 7.77 |
| P45952 | mitochondria | 46.5 | 8.37 |
| P51863 | lysosomes | 40.3 | 5 |
| O09159 | lysosomes | 114.6 | 8.13 |
| Q60931 | mitochondria | 30.7 | 8.79 |
| Q9CR68 | mitochondria | 29.3 | 8.7 |
| Q80U78 | cytoplasm | 126.5 | 6.86 |
| Q9D1J3 | nucleus | 23.5 | 6.65 |
| Q9QYG0 | cytoplasm | 40.8 | 5.4 |
| Q9DCS9 | mitochondria | 21 | 8.03 |
| P35486 | mitochondria | 43.2 | 8.19 |
| Q921G7 | mitochondria | 68 | 7.58 |
| Q91WN1 | cytoplasm；cell membrane；nucleus | 30 | 5.94 |
| Q6RT24 | cytoplasm；cytoskeleton | 286.3 | 5.31 |
| P35293 | cell membrane | 23 | 5.36 |
| Q8JZQ2 | mitochondria | 89.5 | 8.6 |
| P19536 | mitochondria | 13.8 | 8.38 |
| P31266 | cytoplasm；nucleus | 58.5 | 8.13 |
| Q62087 | extracellular | 39.3 | 5.74 |
| Q61102 | mitochondria | 82.5 | 9.32 |
| P52293 | cytoplasm；nucleus | 57.9 | 5.68 |
| P97329 | cytoplasm；cytoskeleton；Golgi apparatus | 99.8 | 6.99 |
| Q9JKB3 | cytoplasm；nucleus | 38.8 | 9.69 |
| O08756 | nucleus；mitochondria | 27.4 | 8.41 |
| Q9EP89 | cytoplasm；mitochondria | 60.7 | 8.9 |
| Q91ZA3 | mitochondria | 79.9 | 7.25 |
| Q80TM9 | cytoplasm；cell membrane | 174.9 | 5.16 |
| Q8C7X2 | endoplasmic reticulum | 111.5 | 7.43 |
| Q99MN9 | mitochondria | 58.4 | 7.66 |
| Q3U0V2 | cytoplasm；nucleus；cytoskeleton | 34.6 | 5.2 |
| Q07417 | mitochondria | 44.9 | 8.47 |
| Q922V4 | nucleus | 56.9 | 9.17 |
| Q99LP6 | mitochondria | 24.3 | 8.38 |
| P20108 | cytoplasm；mitochondria | 28.1 | 7.58 |
| Q99P31 | endoplasmic reticulum | 39.1 | 5.36 |
| Q9JLN9 | cytoplasm；nucleus；mitochondria；cytoskeleton； | 288.6 | 7.17 |

| | | | |
|---|---|---|---|
| | endoplasmic reticulum；lysosomes；Golgi apparatus | | |
| Q6ZQM8 | endoplasmic reticulum | 59.7 | 8.35 |
| P09671 | cytoplasm；mitochondria | 24.6 | 8.62 |
| P42125 | mitochondria | 32.2 | 8.98 |
| Q9DB20 | mitochondria | 23.3 | 9.99 |
| P48024 | | 12.7 | 7.44 |
| P33174 | cytoplasm；nucleus；cytoskeleton | 139.4 | 6.68 |
| Q8K2C9 | endoplasmic reticulum | 43.1 | 9.13 |
| Q60787 | cytoplasm | 60.2 | 6.21 |
| Q9DCE5 | nucleus | 42.1 | 8.47 |
| P99029 | cytoplasm；nucleus；mitochondria；cytoskeleton | 21.9 | 8.85 |
| P38060 | mitochondria | 34.2 | 8.41 |
| Q8CGC6 | nucleus | 84.2 | 9.54 |
| D0QMC3 | nucleus | 60.5 | 9.28 |
| Q8K268 | | 79.8 | 6.16 |
| Q8BGT7 | nucleus | 26.7 | 7.24 |
| A2AJI0 | cytoplasm；cytoskeleton | 93.2 | 10.15 |
| P10922 | nucleus | 20.8 | 10.9 |
| Q8BH95 | mitochondria | 31.5 | 8.48 |
| Q8K4R9 | cytoplasm；nucleus；cytoskeleton | 90.1 | 9.17 |
| Q8BH24 | cell membrane；Golgi apparatus | 74.6 | 7.23 |
| Q2TBE6 | cytoplasm；cell membrane；mitochondria；Golgi apparatus | 54.2 | 8.05 |
| O70503 | | 34.7 | 9.52 |
| P70227 | endoplasmic reticulum | 304.1 | 6.54 |
| O08915 | cytoplasm | 37.6 | 6.4 |
| Q9EQ28 | cytoplasm；nucleus | 50.8 | 9.32 |
| P55096 | | 75.4 | 9.26 |
| Q05117 | lysosomes | 36.8 | 8.9 |
| O88783 | extracellular | 247.1 | 6.05 |
| Q9JJ11 | cytoplasm；cytoskeleton | 70.6 | 4.61 |
| Q80W54 | nucleus；endoplasmic reticulum | 54.7 | 6.95 |
| P97494 | cytoplasm；mitochondria | 72.5 | 5.9 |
| Q80U58 | cytoplasm | 114.2 | 7.08 |
| Q9JLI6 | cytoplasm；Golgi apparatus | 47.1 | 6.8 |
| Q922S8 | cytoplasm；nucleus；cytoskeleton | 81 | 7.97 |
| P52633 | cytoplasm；nucleus | 93.4 | 6.23 |
| Q6ZPY7 | nucleus | 170.8 | 6.92 |
| Q62433 | cytoplasm；cell membrane；nucleus；cytoskeleton | 43 | 6.1 |
| Q8K078 | cell membrane | 77.6 | 7.85 |

| | | | |
|---|---|---|---|
| Q9D1E6 | cytoplasm； cytoskeleton | 27.4 | 5.24 |
| O88531 | extracellular；lysosomes | 34.5 | 8 |
| Q8JZN5 | mitochondria | 68.7 | 7.46 |
| Q8R1V4 | endoplasmic reticulum； Golgi apparatus | 26 | 8.18 |
| Q9D6Y9 | | 80.3 | 6.43 |
| Q8C8U0 | | 108.5 | 5.49 |
| Q8BFZ9 | cytoplasm；endoplasmic reticulum | 37.8 | 5.5 |
| Q9WUM5 | mitochondria | 36.1 | 9.39 |
| O35609 | Golgi apparatus | 38.4 | 7.64 |
| Q99JR1 | mitochondria | 35.6 | 9.23 |
| Q61771 | cytoplasm； cytoskeleton | 85.2 | 7.69 |
| Q9CQF9 | lysosomes | 56.5 | 6.92 |
| Q9Z0J0 | extracellular； endoplasmic reticulum； lysosomes | 16.4 | 7.68 |
| Q8BLH7 | nucleus | 65.2 | 7.84 |
| Q9D172 | mitochondria | 28.1 | 8.78 |
| Q9D379 | endoplasmic reticulum | 52.5 | 8.35 |
| Q3TDN2 | cytoplasm；endoplasmic reticulum | 52.4 | 5.47 |
| Q9ESW4 | mitochondria | 46.9 | 8.4 |
| Q9D0S9 | cytoplasm； mitochondria | 17.3 | 9.82 |
| O08663 | cytoplasm | 52.9 | 5.82 |
| Q64FW2 | endoplasmic reticulum | 67.3 | 8.95 |
| P52825 | nucleus； mitochondria | 73.9 | 8.37 |
| Q9DBS5 | cytoplasm； cytoskeleton | 68.6 | 6.09 |
| Q8R344 | | 18.9 | 7.21 |
| Q8BLF1 | cell membrane； endoplasmic reticulum | 45.7 | 7.05 |
| P56391 | mitochondria | 10.1 | 8.72 |
| Q08024 | nucleus | 22 | 5.77 |
| A3KGF7 | cytoplasm；cell membrane；Golgi apparatus | 134.5 | 6.01 |
| Q3V300 | cytoplasm；nucleus；cytoskeleton | 73.1 | 9.25 |
| P10923 | extracellular | 32.4 | 4.53 |
| Q7TMF3 | mitochondria | 17.1 | 9.36 |
| P53395 | mitochondria | 53.2 | 8.6 |
| Q9D0D4 | cytoplasm； nucleus； mitochondria； cytoskeleton | 35.3 | 9.99 |
| Q9CYR0 | mitochondria | 17.3 | 9.92 |
| Q8CDM1 | nucleus | 117.9 | 6.89 |
| Q9CXE7 | endoplasmic reticulum； Golgi apparatus | 26.2 | 4.93 |
| Q6NXI6 | nucleus | 156.5 | 7.59 |

| | | | |
|---|---|---|---|
| Q80Y14 | mitochondria | 16.3 | 6.55 |
| P09581 | cell membrane；nucleus | 109.1 | 6.21 |
| P30204 | cytoplasm；cell membrane | 50.1 | 6.54 |
| Q9Z0R9 | endoplasmic reticulum | 52.4 | 8.82 |
| Q9Z0H4 | cytoplasm；nucleus | 54.2 | 8.76 |
| P04441 | cell membrane；extracellular；nucleus；endoplasmic reticulum；lysosomes；Golgi apparatus | 31.5 | 8.34 |
| Q3TVI8 | cytoplasm；nucleus；cytoskeleton | 81.1 | 5.36 |
| Q61425 | cytoplasm；nucleus；mitochondria；cytoskeleton | 34.4 | 8.65 |
| Q9CR62 | mitochondria | 34.1 | 9.94 |
| Q9CPV5 | nucleus | 23.1 | 5.26 |
| O88384 | lysosomes | 26.7 | 8.79 |
| Q8R4N0 | mitochondria | 37.5 | 8.54 |
| Q9D666 | cytoplasm；nucleus | 101.9 | 6.81 |
| Q9QXE7 | nucleus | 56.8 | 5.72 |
| Q8R059 | cytoplasm | 38.2 | 6.74 |
| Q9CQC7 | nucleus；mitochondria | 15.1 | 9.89 |
| P03975 | | 62.7 | 9.31 |
| Q9CQY5 | cell membrane；endoplasmic reticulum | 37.9 | 9.76 |
| Q8CFE6 | cell membrane | 55.5 | 7.94 |
| Q9JHW4 | cytoplasm；nucleus；mitochondria；cytoskeleton | 63.5 | 8.29 |
| Q9ERR7 | endoplasmic reticulum | 17.8 | 5.35 |
| Q99KB8 | cytoplasm；mitochondria | 34.1 | 7.75 |
| P47740 | cytoplasm；endoplasmic reticulum | 53.9 | 8.35 |
| Q9CZA6 | cytoplasm；cytoskeleton | 38.5 | 5.34 |
| Q8QZS1 | mitochondria | 43 | 8.06 |
| Q9QZS3 | cytoplasm；cell membrane；nucleus | 70.8 | 8.65 |
| Q9CX56 | nucleus | 39.9 | 9.58 |
| Q9EPK6 | endoplasmic reticulum | 52.4 | 5.27 |
| Q9R0Q4 | nucleus | 32.2 | 9.72 |
| P22315 | mitochondria | 47.1 | 8.91 |
| Q8K0C4 | cytoplasm | 56.7 | 8.41 |
| Q9D1C1 | cytoplasm；nucleus | 19.6 | 7.33 |
| Q3ULF4 | mitochondria | 85.9 | 9.04 |
| Q8VEH8 | endoplasmic reticulum | 54.9 | 6.25 |
| Q9Z1S0 | cytoplasm；nucleus | 118.3 | 5.43 |
| Q9CQE1 | cytoplasm | 28.3 | 9.48 |
| Q8JZK9 | cytoplasm | 57.5 | 5.99 |

| | | | |
|---|---|---|---|
| P30412 | cytoplasm | 22.8 | 7.5 |
| P09450 | nucleus | 35.7 | 9.22 |
| Q9JHI5 | mitochondria | 46.3 | 8.29 |
| Q91VC9 | mitochondria | 37.3 | 9.8 |
| P21126 | cytoplasm； nucleus | 17.8 | 8.44 |
| Q8K009 | nucleus； mitochondria | 101.5 | 6.29 |
| Q64430 | cytoplasm； nucleus； Golgi apparatus | 161.9 | 6.62 |
| E9Q1P8 | cytoplasm； nucleus | 59.3 | 8.69 |
| Q8BWW4 | cytoplasm | 79.7 | 6.39 |
| Q9DB73 | cell membrane | 34.1 | 8.87 |
| P48725 | cytoplasm； extracellular； cytoskeleton | 329.3 | 5.36 |
| Q99JF5 | cytoplasm | 44 | 6.3 |
| Q9QWF0 | nucleus | 101.9 | 5.4 |
| Q9CQL7 | cytoplasm； nucleus | 14.2 | 4.73 |
| Q9D3P8 | cell membrane； mitochondria | 17.3 | 9.5 |
| Q9Z2G9 | cytoplasm； nucleus | 26.9 | 8.59 |
| P07607 | cytoplasm；nucleus；mitochondria | 34.9 | 6.46 |
| Q00422 | nucleus | 51.3 | 5 |
| Q99M54 | cytoplasm | 28.7 | 5.81 |
| P97477 | cytoplasm； cytoskeleton | 44.7 | 9.38 |
| Q9CXX9 | cytoplasm； nucleus | 31.8 | 4.98 |
| Q8R164 | cytoplasm | 32.8 | 8.94 |
| Q9CQV1 | mitochondria | 13.8 | 9.64 |
| Q91XU3 | cytoplasm；nucleus；endoplasmic reticulum | 47.3 | 6.89 |
| O08603 | cell membrane； extracellular | 28.6 | 9.07 |
| Q9Z1P6 | mitochondria | 12.6 | 10.17 |
| Q99L13 | mitochondria | 35.4 | 8.13 |
| Q9CQR4 | cytoplasm； nucleus； mitochondria； cytoskeleton | 15.2 | 8.82 |
| Q9D7P6 | mitochondria | 18.1 | 9.29 |
| Q3TFK5 | | 46.5 | 9.39 |
| P52482 | nucleus | 21.3 | 8.53 |
| P49025 | cytoplasm； nucleus | 235.2 | 6.54 |
| Q8CHY6 | cytoplasm； nucleus | 67.3 | 9.89 |
| Q921J4 | nucleus | 24.2 | 7.91 |
| Q8K021 | Golgi apparatus | 38 | 7.71 |
| P24288 | cytoplasm； mitochondria | 42.8 | 5.39 |
| Q9CWG8 | mitochondria | 48.4 | 6.95 |
| O70126 | cytoplasm；nucleus；cytoskeleton | 39.4 | 9.44 |
| Q80ZV0 | nucleus | 34.7 | 9.45 |

| | | | |
|---|---|---|---|
| Q80YP0 | nucleus | 33.9 | 9 |
| Q99KR7 | mitochondria | 21.7 | 9.16 |
| Q8C166 | cytoplasm；cell membrane；nucleus | 58.8 | 5.66 |
| Q91XD7 | | 45.7 | 5.02 |
| Q8CHT0 | cytoplasm；mitochondria | 61.8 | 8.24 |
| Q99LZ3 | cytoplasm；nucleus | 25.9 | 5.24 |
| Q9CQW9 | cytoplasm；cell membrane；lysosomes | 14.9 | 7.4 |
| Q8VE62 | cytoplasm | 45.7 | 4.55 |
| Q9D7B6 | mitochondria | 45 | 8.13 |
| Q05920 | mitochondria | 129.6 | 6.71 |
| O35604 | extracellular；nucleus；lysosomes；Golgi apparatus | 142.8 | 5.71 |
| Q91ZE0 | mitochondria | 49.6 | 8.25 |
| Q64442 | mitochondria | 38.2 | 7.02 |
| Q9DCZ4 | mitochondria；endoplasmic reticulum；Golgi apparatus | 22.6 | 9.25 |
| Q9CXR1 | | 38.1 | 8.32 |
| Q8JZU2 | mitochondria | 33.9 | 9.89 |
| Q61823 | cytoplasm；nucleus | 51.7 | 5.16 |
| O35405 | nucleus；endoplasmic reticulum；lysosomes；Golgi apparatus | 54.4 | 6.52 |
| Q03267 | cytoplasm；nucleus | 57.3 | 6.52 |
| Q62086 | extracellular；nucleus；mitochondria；lysosomes | 39.6 | 5.83 |
| Q9QYR9 | mitochondria | 49.6 | 7.36 |
| Q3UVK0 | endoplasmic reticulum | 100.1 | 7.49 |
| Q8R080 | cytoplasm；cytoskeleton | 78.7 | 9.39 |
| Q9DBL1 | mitochondria | 47.8 | 7.87 |
| Q8K4F5 | mitochondria | 33.5 | 9.61 |
| Q8BR63 | | 23.6 | 4.59 |
| P24860 | cytoplasm；nucleus；cytoskeleton | 48 | 7.43 |
| Q9R1Z7 | cytoplasm；mitochondria | 16.2 | 6.52 |
| Q05512 | cytoplasm；cell membrane；cytoskeleton | 86.3 | 9.67 |
| Q61846 | cytoplasm；cell membrane | 72.7 | 8.72 |
| Q8CFE2 | nucleus | 39.3 | 7.69 |
| Q8VBZ0 | extracellular | 35.9 | 8.09 |
| Q60780 | cytoplasm；cell membrane | 48.1 | 7.83 |
| Q8VEB4 | extracellular；mitochondria；lysosomes | 47.3 | 6.47 |
| Q9ERG0 | cytoplasm；cell membrane； | 84 | 6.6 |

| | | | |
|---|---|---|---|
| | cytoskeleton | | |
| Q9JJ94 | cytoplasm；nucleus；cytoskeleton | 13.5 | 5.68 |
| Q9CQX4 | cytoplasm； nucleus | 12 | 9.89 |
| Q60929 | nucleus | 53.5 | 7.11 |
| Q9D6M3 | mitochondria | 34.6 | 9.09 |
| Q91XE8 | cell membrane | 21.2 | 9.42 |
| Q921Q3 | endoplasmic reticulum | 54.4 | 8.37 |
| Q8BVK9 | nucleus | 50.1 | 9.86 |
| Q8BHG9 | nucleus | 18.8 | 8.95 |
| Q9JKF6 | cell membrane | 57 | 6.35 |
| P61025 | | 9.7 | 8.94 |
| Q8BYI6 | cell membrane； endoplasmic reticulum； Golgi apparatus | 60.2 | 5.83 |
| Q9CYK1 | cell membrane； nucleus； mitochondria | 40.1 | 8.82 |
| P28741 | cytoplasm； cytoskeleton | 80.1 | 6.54 |
| P31651 | cell membrane | 69.6 | 7.17 |
| Q9CQE7 | endoplasmic reticulum； Golgi apparatus | 43.2 | 6.47 |
| Q8BZR9 | cytoplasm； nucleus | 70 | 5.8 |
| Q8BXN9 | cytoplasm； Golgi apparatus | 63.3 | 6.67 |
| Q8BUE4 | cytoplasm； cell membrane； nucleus； mitochondria | 40.6 | 8.98 |
| Q64281 | cell membrane | 37.5 | 6.21 |
| P19221 | extracellular | 70.2 | 6.43 |
| Q9EQ06 | cytoplasm；endoplasmic reticulum | 32.9 | 8.66 |
| Q9CQS5 | cytoplasm； nucleus | 62.5 | 6.43 |
| Q8K1Z0 | mitochondria | 35.1 | 5.92 |
| Q99N84 | nucleus； mitochondria | 28.7 | 8.35 |
| Q8CCH2 | | 38.2 | 6.23 |
| Q9DAS9 | cell membrane | 8 | 8.97 |
| Q61048 | nucleus | 42.1 | 7.36 |
| Q8K0Z7 | mitochondria | 32.3 | 8.12 |
| A2AWP8 | cytoplasm | 139.9 | 5.82 |
| Q3V1H1 | cytoplasm； cytoskeleton | 74 | 9.23 |
| Q9WVM1 | cytoplasm； cell membrane； nucleus； cytoskeleton | 70.1 | 8.51 |
| Q31125 | nucleus； endoplasmic reticulum； Golgi apparatus | 50.6 | 6.87 |
| P43883 | cytoplasm； nucleus | 46.6 | 6.87 |
| Q8R1G6 | cytoplasm； cytoskeleton | 37.7 | 8.7 |
| Q9Z2Z6 | mitochondria | 33 | 9.11 |
| Q3UDF0 | lysosomes | 54.4 | 8.32 |

| | | | |
|---|---|---|---|
| Q9CPQ1 | mitochondria | 8.5 | 10.14 |
| Q80WR5 | nucleus | 24.8 | 6.04 |
| Q9CQ88 | cell membrane | 22.7 | 8.44 |
| Q922H2 | nucleus；mitochondria | 47.9 | 8.82 |
| Q9WUZ9 | cell membrane；extracellular；endoplasmic reticulum | 47.1 | 5.29 |
| Q9D600 | nucleus | 21.2 | 5.29 |
| Q91WK1 | nucleus；mitochondria | 23.3 | 9.45 |
| Q9R1S3 | cell membrane；endoplasmic reticulum | 105 | 7.58 |
| O35386 | mitochondria | 38.6 | 7.53 |
| Q9D6K5 | cytoplasm；mitochondria | 15.8 | 6.3 |
| Q8BH79 | cell membrane | 76.1 | 6.7 |
| Q9CQ18 | nucleus | 17.8 | 5.8 |
| O35598 | cytoplasm；cell membrane；nucleus；Golgi apparatus | 83.9 | 7.94 |
| Q9CRA5 | cytoplasm；cell membrane；mitochondria；Golgi apparatus | 33.7 | 6.44 |
| Q99J27 | endoplasmic reticulum | 61 | 8.16 |
| Q8C263 | cytoplasm；cytoskeleton | 45.3 | 5.26 |
| P50171 | mitochondria | 26.6 | 6.54 |
| P0C6B7 | | 26.3 | 8.73 |
| Q9D2R6 | mitochondria | 12 | 9.86 |
| Q9ER41 | nucleus；endoplasmic reticulum | 37.8 | 8.02 |
| Q9DCC8 | mitochondria | 16.3 | 8.6 |
| P11881 | cytoplasm；endoplasmic reticulum | 313 | 6.04 |
| Q80TN4 | | 89.1 | 7.55 |
| Q6PG16 | nucleus；mitochondria | 74.2 | 9.35 |
| O55126 | cytoplasm；mitochondria | 32.9 | 9.26 |
| O55125 | mitochondria | 33.3 | 9.44 |
| Q811J3 | cytoplasm；mitochondria；endoplasmic reticulum；Golgi apparatus | 104.9 | 6.93 |
| P0DOV2 | cytoplasm；nucleus | 69.4 | 8.69 |
| Q61703 | extracellular | 105.9 | 7.27 |
| Q9D3B1 | endoplasmic reticulum | 28.4 | 9.58 |
| Q6RUT7 | | 15.3 | 4.93 |
| P61804 | endoplasmic reticulum | 12.5 | 7.08 |
| Q91WA1 | cytoplasm；nucleus | 31.5 | 4.82 |
| Q9D975 | cytoplasm | 14.1 | 8.02 |
| P12023 | cytoplasm；cell membrane；extracellular；nucleus；endoplasmic reticulum；Golgi | 86.7 | 4.79 |

| | | | |
|---|---|---|---|
| | apparatus | | |
| Q3UA16 | nucleus | 26.4 | 6.95 |
| B2RWS6 | cytoplasm；nucleus | 263.1 | 8.54 |
| P56390 | | 9.9 | 8.46 |
| Q8BFY7 | nucleus | 25.7 | 10.92 |
| Q62313 | cell membrane；nucleus；Golgi apparatus | 37.8 | 5.34 |
| Q8C6I2 | cytoplasm；mitochondria | 19.4 | 6.35 |
| O35955 | cytoplasm;nucleus | 29 | 6.87 |
| Q3URE1 | | 65 | 8.02 |
| Q9CZL5 | nucleus；mitochondria | 14.8 | 9.16 |
| Q8R0F8 | cytoplasm；mitochondria | 25.2 | 7.69 |
| A1L314 | cytoplasm | 78.3 | 5.92 |
| O35316 | cell membrane | 69.8 | 7.23 |
| O55003 | nucleus；mitochondria；endoplasmic reticulum | 21 | 7.14 |
| Q8VC04 | cell membrane | 29.1 | 7.44 |
| P15702 | extracellular;nucleus | 40 | 4.96 |
| Q8K370 | mitochondria | 118.9 | 8.24 |
| Q8CIM3 | mitochondria | 58.5 | 7.37 |
| Q08297 | cytoplasm；mitochondria；cytoskeleton | 36.9 | 5.6 |
| Q8C0L8 | cytoplasm；Golgi apparatus | 91.3 | 6.27 |
| Q9D6V8 | cytoplasm | 14.7 | 4.12 |
| P43024 | mitochondria | 12.3 | 9.98 |
| P36371 | endoplasmic reticulum | 77.4 | 7.24 |
| Q9DCA2 | mitochondria | 20.2 | 10.77 |
| E9Q5F9 | nucleus | 285.5 | 6.3 |
| Q91ZW2 | endoplasmic reticulum | 44.7 | 8.41 |
| Q9D9Z1 | cytoplasm;nucleus；cytoskeleton | 34.7 | 5.2 |
| Q9CR10 | mitochondria | 22.2 | 8.05 |
| Q9CYW4 | nucleus；mitochondria | 28 | 6.8 |
| Q922B1 | nucleus；mitochondria | 35.3 | 8.85 |
| Q3V1T4 | extracellular;nucleus；endoplasmic reticulum | 83.6 | 5.14 |
| Q9CR86 | cytoplasm | 16.1 | 8.21 |
| Q3UUI3 | cytoplasm；cell membrane；mitochondria | 26 | 9.64 |
| Q9D023 | mitochondria | 14.3 | 10.61 |
| Q91VE0 | endoplasmic reticulum | 72.3 | 8.59 |
| Q7TMF2 | cytoplasm;nucleus | 39.5 | 8.31 |
| P47930 | nucleus | 35.3 | 7.49 |
| Q9WTX2 | nucleus | 34.3 | 8.43 |

| | | | |
|---|---|---|---|
| O70201 | cytoplasm;nucleus；cytoskeleton | 16.3 | 5.6 |
| Q3UDW8 | lysosomes | 72.5 | 8.27 |
| Q9CZI9 | nucleus | 37.3 | 8.75 |
| P63030 | mitochondria | 12.4 | 9.61 |
| Q7TSY8 | nucleus | 130.2 | 8.76 |
| P22518 | cytoplasm;nucleus | 57.1 | 8.94 |
| Q9ERH4 | cytoplasm;nucleus；cytoskeleton | 48.5 | 9.89 |
| Q3U2A8 | cytoplasm；mitochondria | 118.4 | 7.14 |
| Q9CZN8 | mitochondria | 56.7 | 5.95 |
| Q8CFV9 | cytoplasm；mitochondria | 17.4 | 7.55 |
| Q3UHH1 | cytoplasm | 196.9 | 6.84 |
| P97783 | cytoplasm;nucleus；cytoskeleton | 10 | 4.41 |
| Q9CR46 | cytoplasm；cytoskeleton | 13.7 | 5.55 |
| Q8C0L0 | nucleus | 37.1 | 4.37 |
| Q9CXY9 | endoplasmic reticulum | 44.9 | 6.54 |
| Q8VD57 | cell membrane | 17.5 | 9.09 |
| Q8BH51 | mitochondria | 6.4 | 9.79 |
| Q9D0P0 | endoplasmic reticulum | 23.3 | 6.89 |
| Q00623 | extracellular | 30.6 | 5.73 |
| Q99MK9 | cytoplasm;nucleus；cytoskeleton | 38.8 | 9.04 |
| P51943 | cytoplasm;nucleus | 47.2 | 6.21 |
| Q8CAK1 | mitochondria | 38.4 | 9.01 |
| Q8BHD8 | cytoplasm | 40.7 | 6.46 |
| Q8BJ56 | cell membrane | 53.6 | 6.46 |
| B1B212 | cell membrane；extracellular | 28.2 | 7.71 |
| Q3TC33 | | 30.5 | 9.13 |
| Q9CX66 | nucleus | 20 | 4.84 |
| Q9DAC7 | | 16.8 | 5.4 |
| Q8VDV3 | | 42.7 | 6.38 |
| Q9CRY7 | cytoplasm；endoplasmic reticulum | 35.8 | 8.31 |
| Q3UMR5 | mitochondria | 39.7 | 8.56 |
| Q61070 | nucleus；endoplasmic reticulum；Golgi apparatus | 38.9 | 9.72 |
| Q9JKK7 | cytoplasm；cytoskeleton | 39.5 | 5.35 |
| Q9Z1X9 | nucleus | 65.3 | 5.43 |
| Q8BG21 | | 18.3 | 5.44 |
| P98197 | cell membrane;nucleus；endoplasmic reticulum | 135.4 | 6.48 |
| Q91V16 | mitochondria | 10.4 | 9.73 |
| A2A559 | endoplasmic reticulum；Golgi apparatus | 36.5 | 7.94 |
| Q9Z1T6 | cytoplasm | 236.7 | 6.68 |
| Q9DCI3 | nucleus | 26.8 | 5.02 |

| | | | |
|---|---|---|---|
| Q5NCE8 | mitochondria | 49.3 | 7.53 |
| O88939 | cytoplasm;nucleus | 60.2 | 5.12 |
| Q9CQ00 | mitochondria | 11.3 | 9.39 |
| Q9D818 | cell membrane;nucleus | 42.8 | 8.63 |
| Q14CH7 | mitochondria | 106.7 | 6.18 |
| Q60611 | nucleus | 85.8 | 6.54 |
| Q920Q8 | cytoplasm;nucleus； cytoskeleton | 71.5 | 5.47 |
| Q9DBH0 | extracellular;nucleus | 98.7 | 7.11 |
| O09126 | cell membrane； extracellular | 95.6 | 7.81 |
| Q8BZ36 | cytoplasm；endoplasmic reticulum | 90 | 5.14 |
| Q99PL7 | endoplasmic reticulum | 41.4 | 8.94 |
| Q01339 | cytoplasm； extracellular | 38.6 | 8.22 |
| P60603 | mitochondria | 8.2 | 9.33 |
| Q8K2T4 | cytoplasm;nucleus； mitochondria | 9.6 | 9.52 |
| Q9JKY5 | cytoplasm； cell membrane； mitochondria | 119.4 | 6.52 |

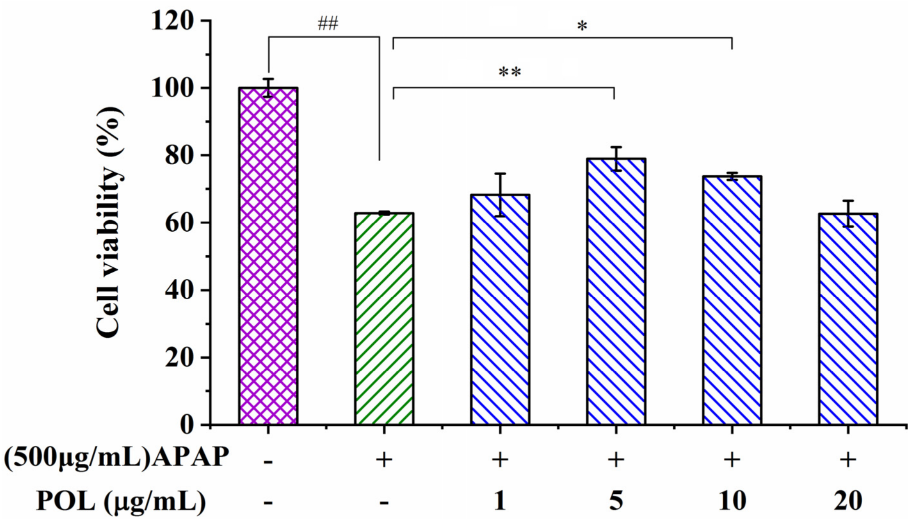


**Figure S1 MTT assay for cell viability.** Effect of acetaminophen (APAP) and polydatin on the survival rate of RAW264.7 cells.

A

B

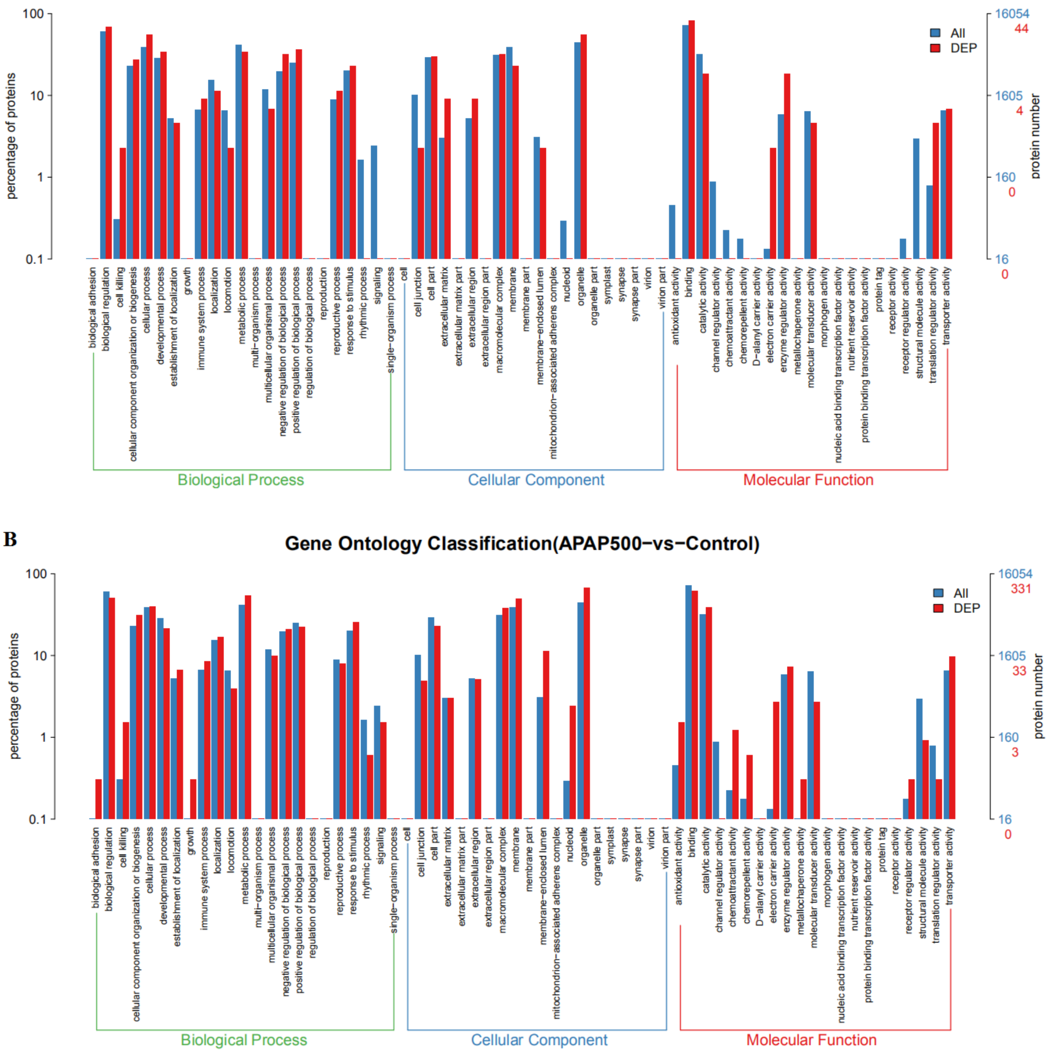


**Figure S2** Gene ontology classification (ANOVA) analysis of differentially expressed proteins of macrophages treated with acetaminophen (APAP). (A) APAP100 vs control group, (B) APAP500 vs control group.
